# Distinct clinical subtypes of genetic frontotemporal dementia identified using disease progression modelling

**DOI:** 10.1093/braincomms/fcag392

**Published:** 2026-10-15

**Authors:** Kiran Samra, Arabella Bouzigues, Phoebe H Foster, Eve Ferry-Bolder, William J Scotton, John C van Swieten, Lize C Jiskoot, Harro Seelaar, Raquel Sanchez-Valle, Robert Laforce, Caroline Graff, Daniela Galimberti, Mario Masellis, Maria Carmela Tartaglia, Rik Vandenberghe, Alexandre de Mendonça, Pietro Tiraboschi, Isabel Santana, Alexander Gerhard, Johannes Levin, Sandro Sorbi, Markus Otto, Thibaud Lebouvier, Maxime Bertoux, Simon Ducharme, Christopher R Butler, Isabelle Le Ber, Elizabeth C Finger, James B Rowe, Matthis Synofzik, Fermin Moreno, Barbara Borroni, Neil P Oxtoby, Lucy L Russell, Jonathan D Rohrer, Alexandra L Young, GENFI, Rhian Convery, Martina Bocchetta, David Cash, Sophie Goldsmith, David L Thomas, Thomas Cope, Timothy Rittman, Antonella Alberici, Enrico Premi, Roberto Gasparotti, Emanuele Buratti, Valentina Cantoni, Andrea Arighi, Chiara Fenoglio, Vittoria Borracci, Maria Serpente, Tiziana Carandini, Emanuela Rotondo, Giacomina Rossi, Giorgio Giaccone, Giuseppe Di Fede, Paola Caroppo, Sara Prioni, Veronica Redaelli, David Tang-Wai, Ekaterina Rogaeva, Miguel Castelo-Branco, Morris Freedman, Ron Keren, Sandra Black, Sara Mitchell, Christen Shoesmith, Robart Bartha, Rosa Rademakers, Jackie Poos, Janne M Papma, Lucia Giannini, Liset de Boer, Julie de Houwer, Rick van Minkelen, Yolande Pijnenburg, Benedetta Nacmias, Camilla Ferrari, Cristina Polito, Gemma Lombardi, Valentina Bessi, Mattias Nilsson, Henrik Viklund, Melissa Taheri Rydell, Vesna Jelic, Linn Öijerstedt, Tobias Langheinrich, Albert Lladó, Anna Antonell, Jaume Olives, Mircea Balasa, Nuria Bargalló, Sergi Borrego-Ecija, Ana Verdelho, Carolina Maruta, Tiago Coelho, Gabriel Miltenberger, Frederico Simões do Couto, Alazne Gabilondo, Ioana Croitoru, Mikel Tainta, Myriam Barandiaran, Patricia Alves, Benjamin Bender, David Mengel, Lisa Graf, Annick Vogels, Mathieu Vandenbulcke, Philip Van Damme, Rose Bruffaerts, Koen Poesen, Pedro Rosa-Neto, Maxime Montembault, Agnès Camuzat, Alexis Brice, Anne Bertrand, Aurélie Funkiewiez, Daisy Rinaldi, Dario Saracino, Olivier Colliot, Sabrina Sayah, Catharina Prix, Elisabeth Wlasich, Olivia Wagemann, Sonja Schönecker, Alexander Maximilian Bernhardt, Anna Stockbauer, Jolina Lombardi, Sarah Anderl-Straub, Adeline Rollin, Gregory Kuchcinski, Maxime Bertoux, Thibaud Lebouvier, Vincent Deramecourt, João Durães, Marisa Lima, Maria João Leitão, Maria Rosario Almeida, Miguel Tábuas-Pereira, Sónia Afonso, João Lemos

**Affiliations:** Dementia Research Centre, Department of Neurodegenerative Disease, UCL Queen Square Institute of Neurology, London WC1N 3AR, UK; Dementia Research Centre, Department of Neurodegenerative Disease, UCL Queen Square Institute of Neurology, London WC1N 3AR, UK; Dementia Research Centre, Department of Neurodegenerative Disease, UCL Queen Square Institute of Neurology, London WC1N 3AR, UK; Dementia Research Centre, Department of Neurodegenerative Disease, UCL Queen Square Institute of Neurology, London WC1N 3AR, UK; Dementia Research Centre, Department of Neurodegenerative Disease, UCL Queen Square Institute of Neurology, London WC1N 3AR, UK; Department of Neurology, Erasmus Medical Center, 3000 CA Rotterdam, The Netherlands; Department of Neurology, Erasmus Medical Center, 3000 CA Rotterdam, The Netherlands; Department of Neurology, Erasmus Medical Center, 3000 CA Rotterdam, The Netherlands; Alzheimer’s Disease and Other Cognitive Disorders Unit, Neurology Service, Hospital Clínic, Institut D’Investigacións Biomèdiques August Pi I Sunyer, University of Barcelona, 08036 Barcelona, Spain; Clinique Interdisciplinaire de Mémoire, Département des Sciences Neurologiques, CHU de Québec, and Faculté de Médecine, Université Laval, Quebec City, QC G1J 1Z4, Canada; Department of Neurobiology, Care Sciences and Society, Center for Alzheimer Research, Division of Neurogeriatrics, Bioclinicum, Karolinska Institutet, 171 64 Solna, Sweden; Unit for Hereditary Dementias, Theme Inflammation and Aging, Karolinska University Hospital, 171 64 Solna, Sweden; Fondazione Ca’ Granda, IRCCS Ospedale Policlinico, 20122 Milan, Italy; University of Milan, Centro Dino Ferrari, 20122 Milan, Italy; Hurvitz Brain Sciences Research Program, Sunnybrook Research Institute, University of Toronto, Toronto, ON M4N 3M5, Canada; L. C. Campbell Cognitive Neurology Research Unit, Sunnybrook Health Sciences Centre, Toronto, ON M4N 3M5, Canada; Tanz Centre for Research in Neurodegenerative Diseases, University of Toronto, Toronto, ON M5T 0S8, Canada; Laboratory for Cognitive Neurology, Department of Neurosciences, KU Leuven, 3000 Leuven, Belgium; Neurology Service, University Hospitals Leuven, 3000 Leuven, Belgium; Faculty of Medicine, University of Lisbon, 1649-028 Lisbon, Portugal; Fondazione IRCCS Istituto Neurologico Carlo Besta, 20133 Milano, Italy; University Hospital of Coimbra (HUC), Neurology Service, Faculty of Medicine, University of Coimbra, 3004-561 Coimbra, Portugal; Center for Neuroscience and Cell Biology, Faculty of Medicine, University of Coimbra, 3004-504 Coimbra, Portugal; Division of Psychology Communication and Human Neuroscience, Wolfson Molecular Imaging Centre, University of Manchester, Manchester M20 3JJ, UK; Department of Nuclear Medicine, Center for Translational Neuro- and Behavioral Sciences, University Medicine Essen, 45147 Essen, Germany; Department of Geriatric Medicine, Klinikum Hochsauerland, 59759 Arnsberg, Germany; Department of Neurology, Ludwig-Maximilians Universität München, 80336 Munich, Germany; German Center for Neurodegenerative Diseases (DZNE), 81377 Munich, Germany; Department of Neurofarba, University of Florence, 50139 Florence, Italy; IRCCS Fondazione Don Carlo Gnocchi, 50143 Florence, Italy; Department of Neurology, University of Ulm, 89081 Ulm, Germany; Univ Lille, Lille 59000, France; Inserm 1172, Lille 59000, France; Univ Lille, Lille 59000, France; Inserm 1172, Lille 59000, France; Department of Psychiatry, McGill University Health Centre, McGill University, Montreal, QC H4A3J1, Canada; McConnell Brain Imaging Centre, Montreal Neurological Institute, McGill University, Montreal, QC H3A 2B4, Canada; Nuffield Department of Clinical Neurosciences, Medical Sciences Division, University of Oxford, Oxford OX3 9DU, UK; Department of Brain Sciences, Imperial College London, London W12 0NN, UK; Sorbonne Université, Paris Brain Institute – Institut du Cerveau – ICM, Inserm U1127, CNRS UMR 7225, AP-HP - Hôpital Pitié-Salpêtrière, Paris 75013, France; Centre de référence des démences rares ou précoces, IM2A, Département de Neurologie, AP-HP - Hôpital Pitié-Salpêtrière, Paris 75013, France; Department of Clinical Neurological Sciences, University of Western Ontario, London, ON N6A 5A5, Canada; Department of Clinical Neurosciences and Cambridge University Hospitals NHS Trust, University of Cambridge, Cambridge CB2 0QQ, UK; Department of Neurodegenerative Diseases, Hertie-Institute for Clinical Brain Research and Center of Neurology, University of Tübingen, Tübingen 72076, Germany; Center for Neurodegenerative Diseases (DZNE), Tübingen 72076, Germany; Cognitive Disorders Unit, Department of Neurology, Hospital Universitario Donostia, San Sebastian 20014, Spain; Center for Biomedical Research in Neurodegenerative Disease (CIBERNED), Carlos III Health Institute, Madrid 28031, Spain; Neurology Unit, Department of Clinical and Experimental Sciences, University of Brescia, Brescia 25121, Italy; Molecular Markers Laboratory, IRCC Istituto Centro San Giovanni di Dio Fatebenefratelli, Brescia 25125, Italy; UCL Hawkes Institute and Department of Computer Science, University College London, London WC1V 6LJ, UK; Dementia Research Centre, Department of Neurodegenerative Disease, UCL Queen Square Institute of Neurology, London WC1N 3AR, UK; Dementia Research Centre, Department of Neurodegenerative Disease, UCL Queen Square Institute of Neurology, London WC1N 3AR, UK; UCL Hawkes Institute and Department of Computer Science, University College London, London WC1V 6LJ, UK

**Keywords:** subtyping, staging, c9orf72, GRN, MAPT

## Abstract

Understanding the order in which symptoms occur in genetic frontotemporal dementia (FTD) may provide valuable insights into disease progression. Here we use a methodology called Ordinal Subtype and Stage Inference (SuStaIn) in the GENetic FTD Initiative (GENFI) cohort to estimate a probable order in which symptoms occur and identify different subtypes based on the pattern of symptom progression within the main genetic FTD mutation groups. A total of 968 participants were included in the cross-sectional study: 280 *C9orf72*, 242 *GRN* and 101 *MAPT* mutation carriers, and 345 mutation-negative controls. A total of 379 participants underwent longitudinal analysis: 162 *C9orf72*, 146 *GRN* and 71 *MAPT* mutation carriers. The standardized GENFI clinical assessment provided severity scores for all FTD-relevant symptoms ranging from 0, where symptoms are absent, to 3, where symptoms are severe. Ordinal SuStaIn was run in each mutation group to generate symptom progression profiles for mutation carriers from each group. In the *C9orf72* cohort, Ordinal SuStaIn identified three subtypes: subtype 1 with early behavioural and cognitive deficits, subtype 2 with impaired speech and swallowing and subtype 3 with limb impairment. In the *GRN* cohort, SuStaIn identified two subtypes: subtype 1 with behavioural and cognitive deficits as well as limb motor involvement, and subtype 2 with early language impairment. The *MAPT* cohort was best described as a single subtype with a combination of cognitive (mainly semantic) and behavioural impairment with parkinsonism. SuStaIn stage significantly correlated with clinical severity scores for all the cohorts. The subtypes were somewhat longitudinally consistent, with a trend towards increased SuStaIn stage in each subtype over time. Identifying within-genotype data-driven subtypes of FTD is a novel approach for untangling the heterogeneity of this disorder, in turn drawing us closer to individualized care.

## Graphical Abstract

**Graphical Abstract:**
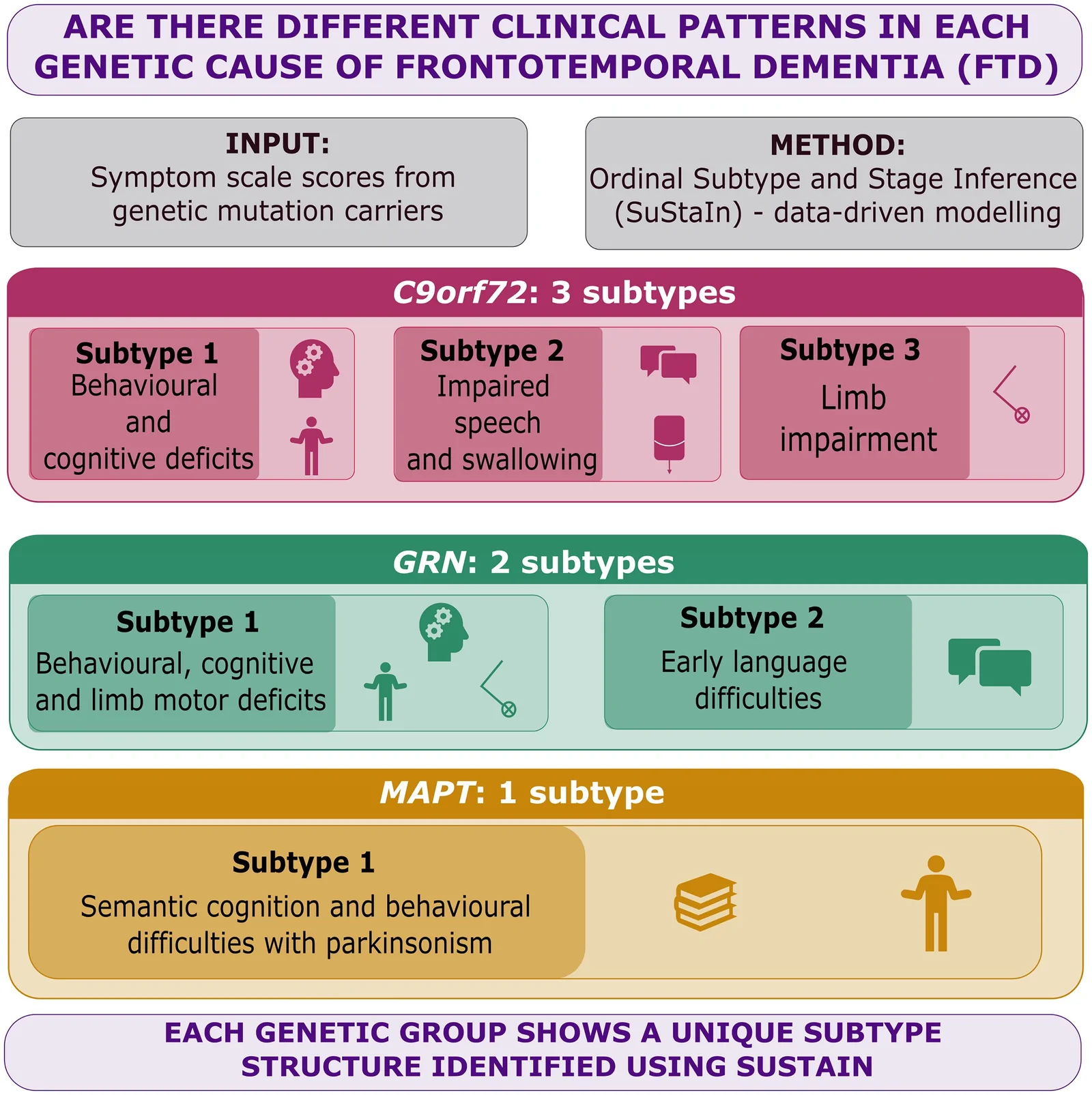

## Teaser

Samra *et al.* use novel disease progression modelling, ordinal Subtype and Stage Inference, applied to data from the international Genetic Frontotemporal dementia Initiative to understand the order in which symptoms occur in genetic frontotemporal dementia, in an effort to untangle the heterogeneity of frontotemporal dementia.

## Introduction

Frontotemporal dementia (FTD) is a progressive neurodegenerative disorder of behavioural, neuropsychiatric, linguistic, cognitive, motor and functional deficits. There is phenotypic heterogeneity amongst individuals, even within single families who inherit an FTD-related genetic mutation.^1,2^ Variants in three genes account for the majority of genetic FTD*—C9orf72*, *GRN* and *MAPT—*and the symptomatology, neuropsychological and brain imaging findings of each of these genetic groups have been well-studied.^1-8^ The temporal ordering of such measures has been investigated now in a number of reports,^9-11^ although the majority have focused on imaging markers rather than clinical symptoms. Understanding the ordering of such symptoms, and identifying subtypes that form within a mutation group will allow us to observe the earliest changes that affect a person. This is important as it may influence inclusion criteria, the targeting of disease-modifying therapies and the focus of clinical trial outcomes.

The Ordinal Subtype and Stage Inference (SuStaIn) algorithm is a scored-events model of disease progression applied to ordinal data with the aim of identifying groups with unique disease progression patterns.^10-12^ SuStaIn has previously been applied to quantitative data, and this is the first application of this type of model specifically to ordinal data. Ordinal SuStaIn can be applied to clinical symptom rating scale scores in FTD, such as those investigated in the GENetic FTD Initiative (GENFI) study, which are ordinal data that describe how severely affected an individual is with a particular symptom. As such data are collected prior to clinical diagnosis, i.e. meeting diagnostic criteria for an FTD spectrum disorder, ordering clinical symptom rating scale data in GENFI allows us to observe the earliest changes that affect a person and the probable disease trajectory.

This study therefore aims to apply Ordinal SuStaIn to the GENFI cohort in order to bring us closer to understanding the entire trajectory of clinical progression in genetic FTD, with the ultimate goal of allowing targeting of specific therapies to certain subtypes early enough to make a difference to the lives of individuals with FTD.

## Materials and methods

### Participants

Study participants were obtained from the sixth data freeze of GENFI, a cohort study that recruits carriers of FTD-causing genetic mutations (the affected group) and mutation-negative first-degree relatives (the control group). Clinical diagnoses of the baseline visit cohort (made by a clinician at or prior to entry into GENFI) include: behavioural variant frontotemporal dementia (bvFTD) (138 individuals), primary progressive aphasia (PPA) (32), amyotrophic lateral sclerosis (ALS) or FTD–ALS (27), corticobasal syndrome (CBS) (2), progressive supranuclear palsy (PSP) (2) or other dementia diagnoses (7). In the cross-sectional study, we included all individuals with a mutation in *C9orf72* (280), *GRN* (242) and *MAPT* (101) who had completed the GENFI symptom scales, and 345 controls. The CDR® plus NACC FTLD is a scale often used in clinical trials to stratify patients based on severity of behavioural, cognitive and language symptoms: 0 = absent, 0.5 = questionable/very mild, 1 = mild, 2 = moderate, 3 = severe symptoms. Criteria used to assess severity in this study are as per the CDR® plus NACC FTLD, with the addition of neuropsychiatric and motor domains (CDR® plus NACC FTLD-NM).^5,8,13^ The assessment consists of a semi-structured interview with inclusion of both the participant and an informant to generate an overall clinician-judged score for each symptom. All controls in the study had a CDR® plus NACC FTLD global score of <1. In the longitudinal study, we included all *C9orf72* (162), *GRN* (146) and *MAPT* (71) mutation carriers with up to three follow-up visits in which the GENFI symptom scales were completed.

### Clinical data

All individuals completed the standardized GENFI assessment that includes a clinical history and examination, and cognitive assessment. The GENFI symptom scales consist of severity scores for each cognitive, behaviour, neuropsychiatric and motor domain relevant to FTD, with 45 symptoms included within this study based on previous work^5,13^ (see Supplementary Appendix, Supplementary Table 7 for list of symptoms). Ordinal SuStaIn was used to identify subgroups of the three main FTD mutation carrier groups (*C9orf72*, *GRN*, *MAPT*) with distinct progression patterns based on cross-sectional data. SuStaIn groups individuals into subtypes and infers the disease progression pattern within each subtype using disease progression modelling.^11^ Each disease progression pattern in Ordinal SuStaIn is described by a set of stages, where each stage corresponds to a particular symptom reaching a particular score. We visualize the uncertainty in the maximum likelihood progression pattern using positional variance diagrams (PVDs).^14^ In a PVD, symptom variables are listed in the order in which they are inputted into the programme and outputs are colour-coded such that red represents progression from a severity score of 0 (absent) to 0.5 (possible/very mild) at a particular stage for that symptom; magenta for a score increase from 0.5 to 1 (mild); blue for a score increase from 1 to 2 (moderate); and black for a score increase from 2 to 3 (severe). The lighter the shading, the more uncertain the position of this score transition in the sequence. More specifically, on one end of the scale, red/magenta/blue/black indicate a probability of 1 that a particular score appears at that position in the sequence, and at the opposite end of the scale, white indicates a probability of 0 that a particular score appears at that position in the sequence (Figs 1–6, Supplementary Figs 5, and 7–9).

**Figure 1:**
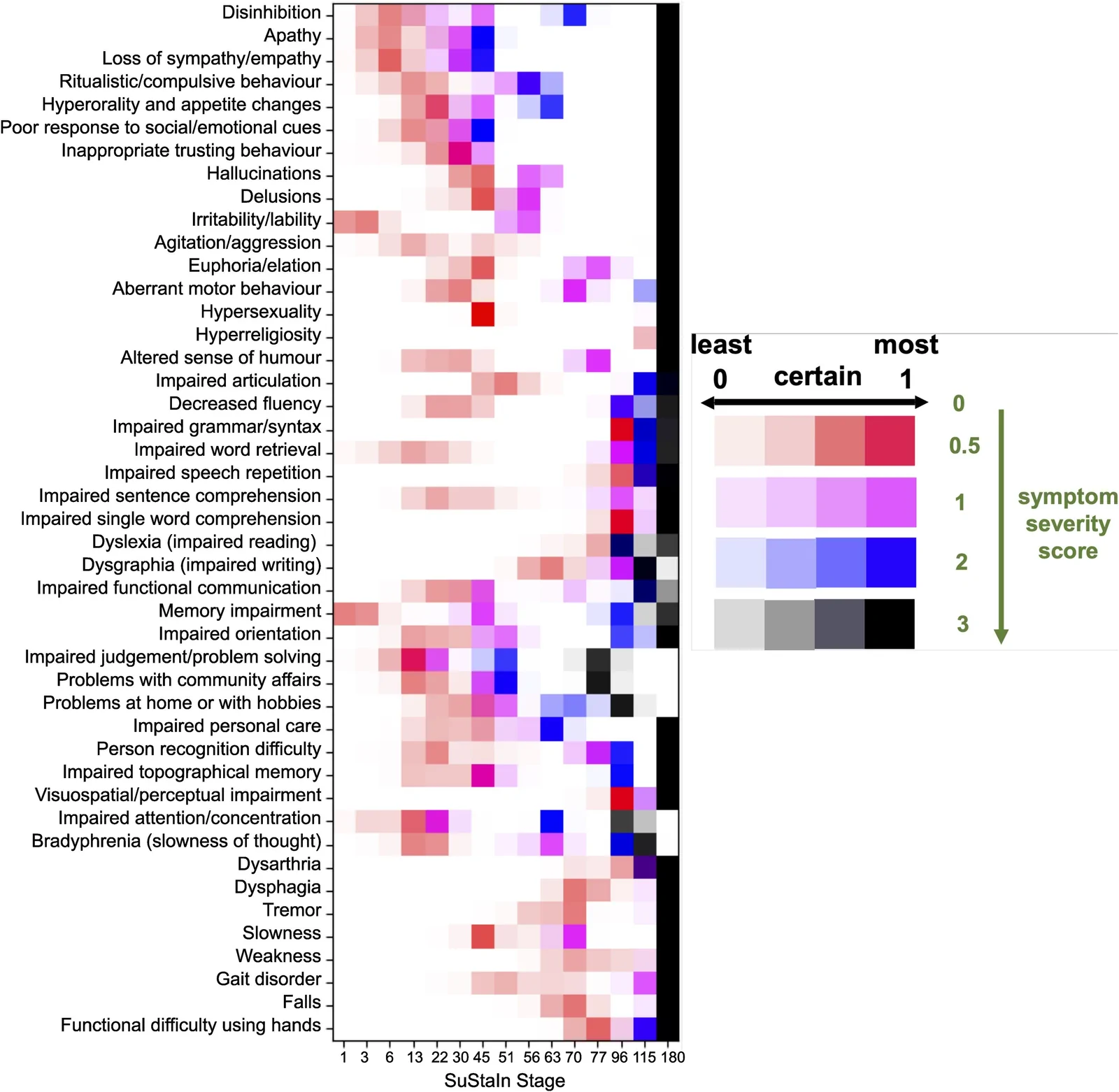
**Ordinal SuStaIn positional variance diagram for *C9orf72* subtype 1, the bvFTD-like group (*N* = 88), as identified by cross-validation.** SuStaIn stages are compressed to show at least five individuals per stage. Full outputs are displayed in Supplementary Fig. 5. A heatmap scale bar is displayed to show what each square colour represents. Each coloured square represents a change in a symptom severity score in the individuals within the group. The range includes: 0 = white, indicating no change; a score change from 0 to 0.5 = red; from 0.5 to 1 = magenta; from 1 to 2 = blue; and from 2 to 3 = black. The darker the square is shaded, the more certain the algorithm is that this output should be positioned at that particular stage in the sequence.

**Figure 2:**
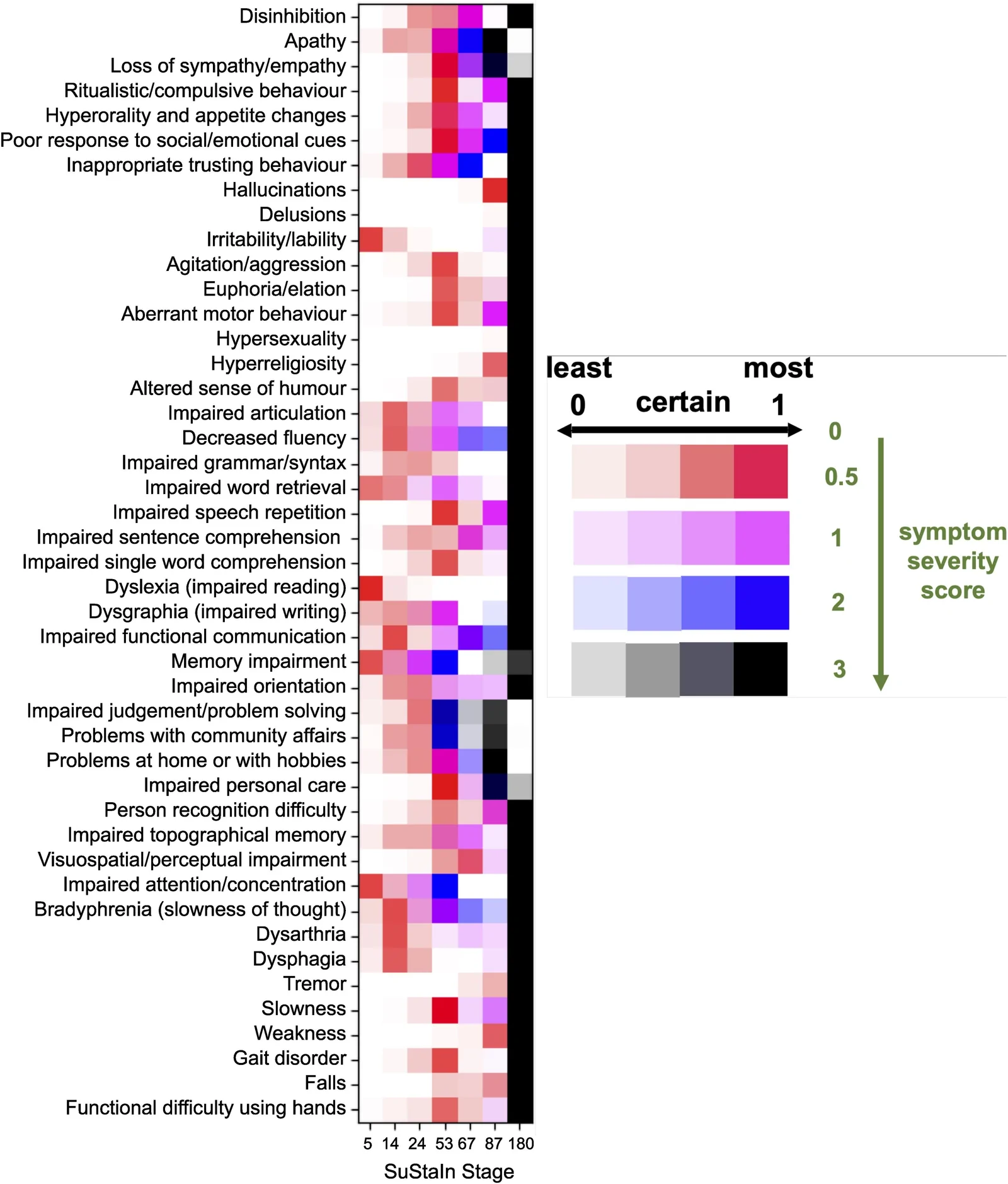
**Ordinal SuStaIn positional variance diagram for *C9orf72* subtype 2, the bulbar ALS-like group (*N* = 35), as identified by cross-validation.** SuStaIn stages are compressed to show at least five individuals per stage. Full outputs are displayed in Supplementary Fig. 5. A heatmap scale bar is displayed to show what each square colour represents. Each coloured square represents a change in a symptom severity score in the individuals within the group.

**Figure 3:**
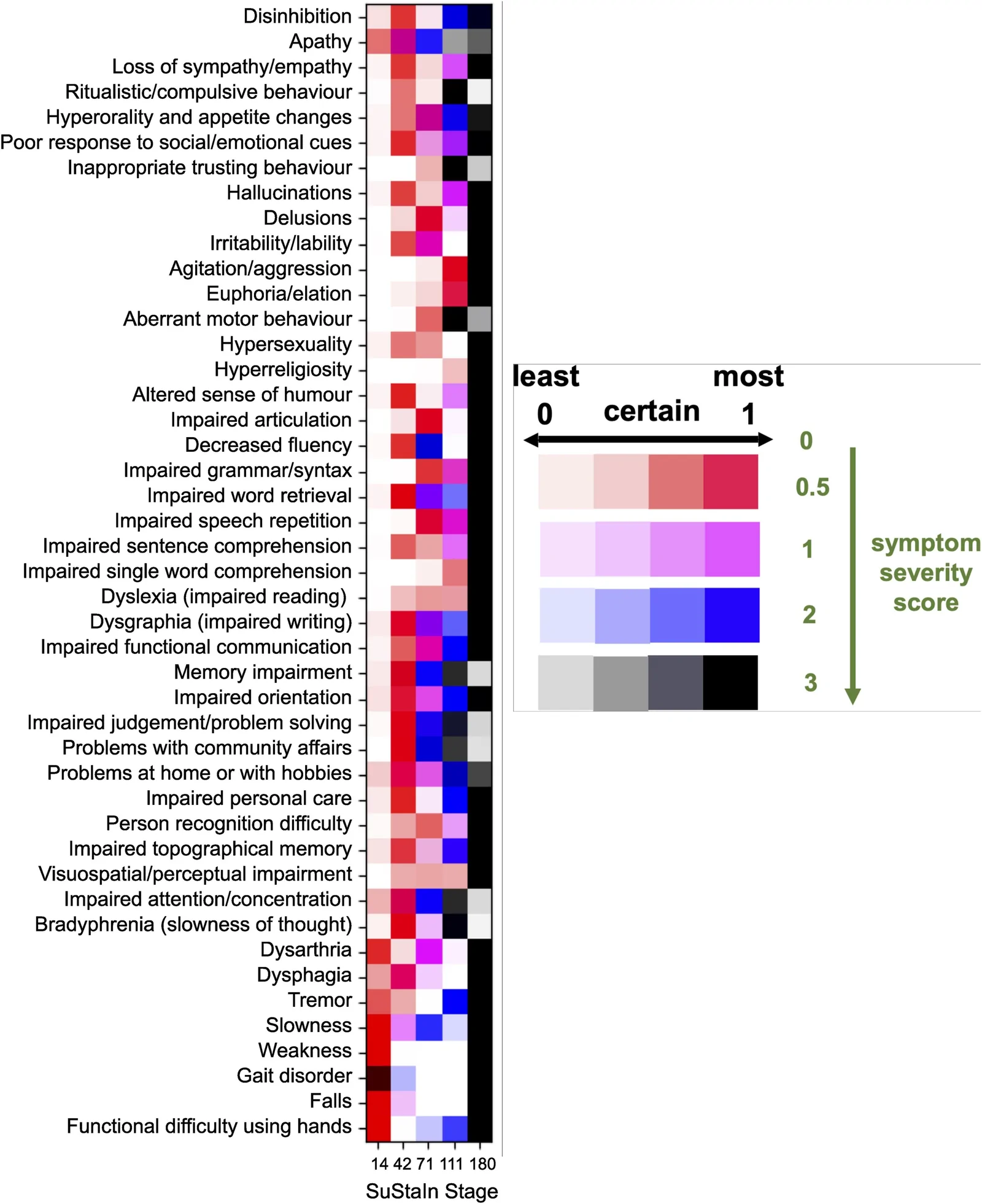
**Ordinal SuStaIn positional variance diagram for *C9orf72* subtype 3, the limb ALS-like group (*N* = 22), as identified by cross-validation.** SuStaIn stages are compressed to show at least five individuals per stage. Full outputs are displayed in Supplementary Fig. 5. A heatmap scale bar is displayed to show what each square colour represents. Each coloured square represents a change in a symptom severity score in the individuals within the group.

**Figure 4:**
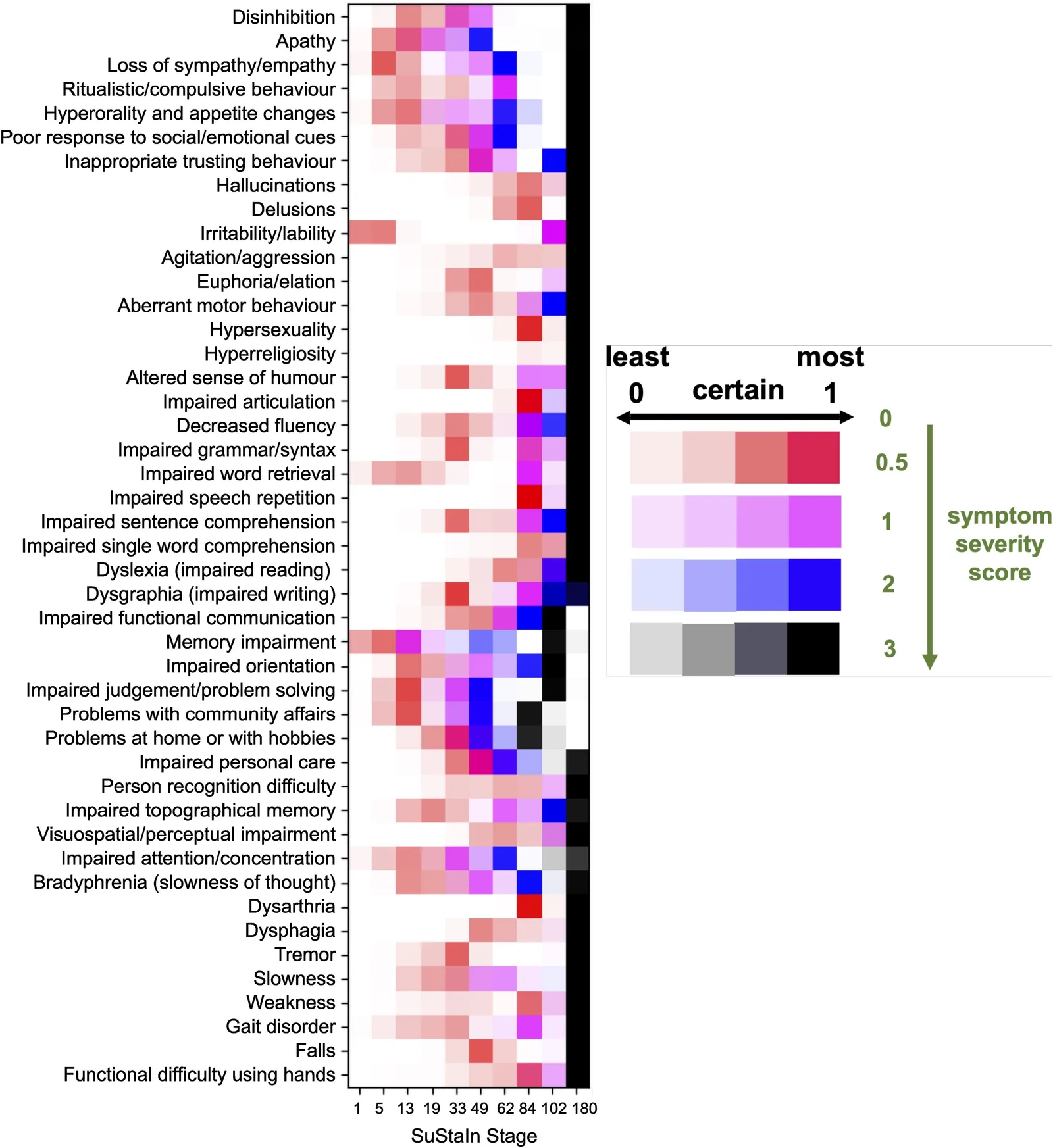
**Ordinal SuStaIn positional variance diagram for *GRN* subtype 1, the bvFTD–parkinsonism-like group (*N* = 52), as identified by cross-validation.** SuStaIn stages are compressed to show at least five individuals per stage. Full outputs are displayed in Supplementary Fig. 5. A heatmap scale bar is displayed to show what each square colour represents. Each coloured square represents a change in a symptom severity score in the individuals within the group.

**Figure 5:**
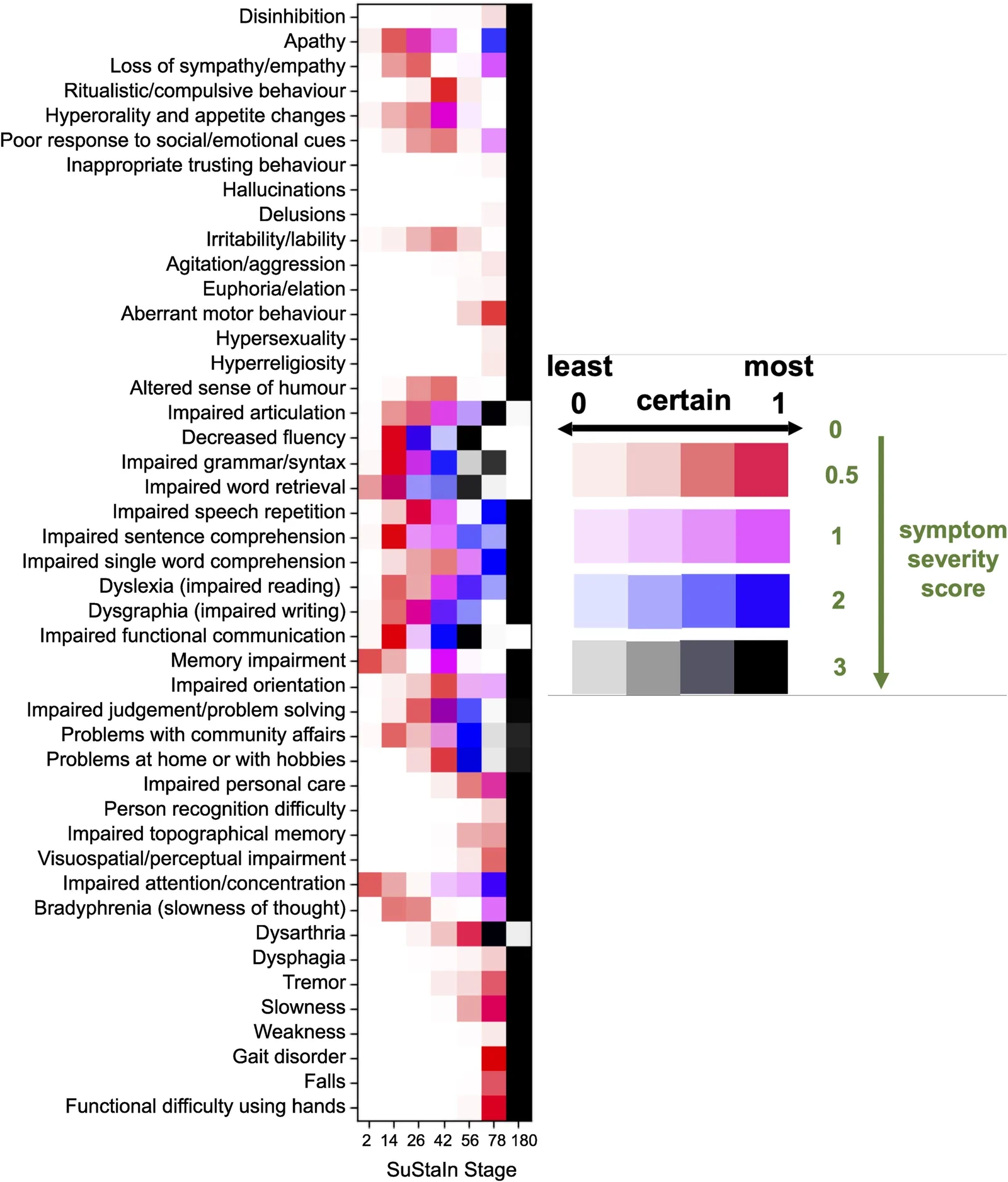
**Ordinal SuStaIn positional variance diagram for *GRN* subtype 2, the PPA-like group (*N* = 37), as identified by cross-validation.** SuStaIn stages are compressed to show at least five individuals per stage. Full outputs are displayed in Supplementary Fig. 5. A heatmap scale bar is displayed to show what each square colour represents. Each coloured square represents a change in a symptom severity score in the individuals within the group.

**Figure 6:**
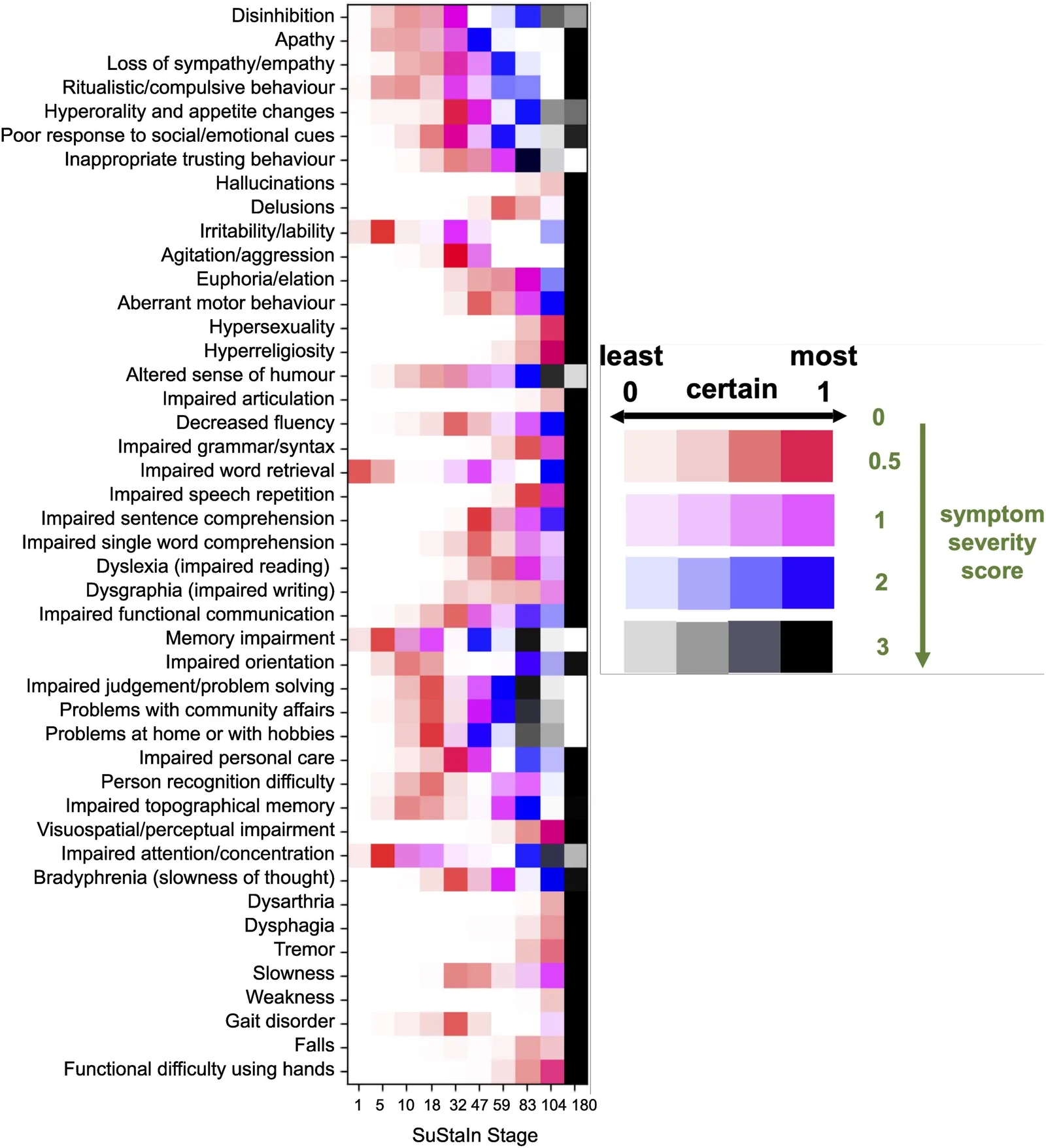
**Ordinal SuStaIn positional variance diagram for the *MAPT* semantic-behavioural-parkinsonism group (*N* = 49), as identified by cross-validation.** SuStaIn stages are compressed to show at least five individuals per stage. Full outputs are displayed in Supplementary Fig. 5. A heatmap scale bar is displayed to show what each square colour represents. Each coloured square represents a change in a symptom severity score in the individuals within the group.

### Assigning participants to subtypes and stages

Ordinal SuStaIn was run on each mutation group separately, initially with a single subtype to determine the dominant progression pattern within each mutation group. The overlap between symptom progression patterns was explored by comparing the model-assigned probability that individuals from each mutation group matched the subtype progression pattern for their own mutation group versus each of the other two mutation groups.

Ordinal SuStaIn was further used to infer subtypes with distinct progression patterns within each mutation group to determine the heterogeneity in symptom progression patterns within each mutation group. Cross-validation was performed to inform the number of subtypes present in each mutation group.^15^ This showed that the heterogeneity in symptom progression patterns is best described by a three-subtype model in *C9orf72* mutation carriers, a two-subtype model in *GRN* mutation carriers and a single symptom progression profile in *MAPT* mutation carriers (Supplementary Figs 1 and 2). Individuals were assigned to subtypes and stages based on their maximum likelihood subtype and stage. Those with a stage of 0 (no or minimal abnormality on any clinical symptom score) were assigned to a separate subtype 0 group. We refer to individuals with a stage greater than 0 as ‘subtypable’.

### Statistical analysis

All statistical analyses were performed using Stata/MP 16.1. Statistical tests of normality were performed using the Shapiro–Wilk test. Sex differences between groups were compared using a chi-squared test. Age, education years and clinical scores were compared between mutation groups and controls using linear regression (95% bias-corrected bootstrapped confidence intervals with 2000 repetitions due to non-normal distribution), and between mutation groups using ordinal logistic regression, adjusting for age and sex where applicable.

The Ordinal SuStaIn staging outputs were correlated with the following measures of clinical severity: CDR® plus NACC FTLD-Sum of Boxes (SOB),^16-18^ the CDR® plus NACC FTLD-NM-SOB that added neuropsychiatric and motor features to the scales and was shown to improve detection of the appropriate FTD stage in previous work,^13^ the FTD Rating Scale (FRS),^19-21^ which focuses on functional, cognitive and behavioural impairment and the ALS-Functional Rating Scale-Revised (ALSFRS-R),^22,23^ a validated scale for ALS symptoms.

R version 4.1.2^24^ was used to fit a linear model score comparing mutation-specific subtypes, SuStaIn stage, age, sex and educational attainment level for each symptom severity score, including data from all subtypable mutation carriers with more than one subtype within a mutation group. This included *C9orf72* subtypes 1, 2 and 3, and in a separate comparison, *GRN* subtypes 1 and 2. Statistical significance was reported at a level of *P* < 0.05. We reported which subtype had the worst performance on each symptom score based on the subtype with the maximum *t*-value, however if there was <0.5 difference in *t*-values between a subset of subtypes, we denoted each of the subtypes within the subset as having poor performance on that symptom score.

### Longitudinal validation

The SuStaIn model fitted on the cross-sectional baseline visit data was used to assign the maximum likelihood stage and subtype to all follow-up symptom severity score data. Data from up to three follow-up visits were used to validate the stability of subtypes and to assess stage progression. Greater stability is achieved if most individuals remain in the same subtype yet advance to higher stages over time (or remain at the same stage). To measure subtype stability, the proportion of individuals assigned to the same subtype within a mutation group at follow-up(s) or who progressed from stage 0 (subtype 0; defined as having ‘no/minimal symptoms’) into a subtype was compared to the total number of individuals within that mutation group. To measure stage progression, the SuStaIn stage at baseline and follow-up visit for all individuals within a mutation group were compared by calculating the proportion of individuals that progressed or stayed at the same stage for symptom severity scoring at the follow-up visit.

## Results

### Demographics

There were no significant sex differences apart from fewer males with *GRN* mutations compared with the *C9orf72* group (*Chi^2^* = 6.88, *P* = 0.009). *C9orf72* and *GRN* groups were significantly older than controls and *MAPT* mutation carriers (all *P* < 0.001). *C9orf72* mutation carriers had significantly fewer years in education than controls (*P* = 0.001). There were significantly fewer Expected Years to Onset (EYO) in the genetic groups compared with controls (all *P* < 0.001), with the least EYO in *MAPT* compared with the most in *GRN* mutation carriers (Table 1).

**Table 1:** Demographics and clinical scores for the mutation groups and controls

	Controls	C9orf72	GRN	MAPT
Number of participants	345	280	242	101
% Male	44.1	50.7	39.3^a^	45.5
Age in years	45.6 (12.9)	**51.4** (**13.8)^b^**	**51.0** (**13.4)^b^**	45.3 (13.1)
Expected years to onset	−12.9 (13.5) or N/A	**−7.0** (**12.9)^c^**	**−9.9** (**13.6)**	**−6.9 (12.8)^a,c^**
Education in years	14.6 (3.3)	**13.7** (**3.3)**	14.1 (3.8)	14.1 (3.3)
CDR® plus NACC FTLD-Global Score	0.1 (0.2)	**0.8** (**1.0)^c^**	**0.6** (**0.9)**	**0.7** (**1.0)^c^**
CDR® plus NACC FTLD-Sum of Boxes	0.1 (0.4)	**4.0 (6.1)^b,c^**	**2.7** (**5.2)**	**3.4** (**5.6)^c^**
CDR® plus NACC FTLD-NM-Global Score	0.1 (0.2)	**0.8** (**1.0)^c^**	**0.6** (**0.9)**	**0.7** (**1.0)^c^**
CDR® plus NACC FTLD-NM-Sum of Boxes	0.2 (0.5)	**4.5** (**6.8)^c^**	**2.9** (**5.7)**	**3.5** (**5.8)^c^**
FTD Rating Scale	96.9 (6.5)	**71.7** (**33.5)^c^**	**81.7** (**28.3)**	**76.5** (**28.9)^c^**

Data are shown as mean (standard deviation). Bold items are significantly different to controls.

^a^Significantly fewer males/expected years to onset (EYO) compared to *C9orf72* mutation carriers.

^b^Significantly older/severe compared to *MAPT* mutation carriers.

^c^Significantly severe/fewer EYO compared to *GRN* mutation carriers. FTD Rating Scale (FRS) was performed on 274 controls and 521 mutation carriers (233 *C9orf72*, 202 *GRN*, 86 *MAPT* mutation carriers). In the cross-sectional data at the baseline visit, of those with a *C9orf72* mutation, 47.9% had a CDR 0, 17.9% a CDR 0.5, 9.3% a CDR 1, 13.6% a CDR 2 and 11.8% a CDR 3. Of those with a *GRN* mutation, 59.5% had a CDR 0, 14.5% a CDR 0.5, 10.3% a CDR 1, 7.9% a CDR 2 and 7.9% a CDR 3. Of those with a *MAPT* mutation, 49.5% had a CDR 0, 19.8% a CDR 0.5, 9.9% a CDR 1, 11.9% a CDR 2 and 8.9% a CDR 3.

### Disease severity

The CDR® plus NACC FTLD, CDR® plus NACC FTLD-NM and FRS scores were indicative of worse disease severity in all mutation groups compared to controls (all *P* < 0.001) with *C9orf72* and *MAPT* groups generally scoring worse than *GRN* mutation carriers in most measures (Table 1). Supplementary Table 1 displays results for the other measures of FTD severity (MMSE, PASS SOB, ALSFRS-R and CBI-R).

### Association between mutation group and subtype assignment

Single progression patterns and the probability comparisons between them are shown in the Supplementary Appendix (Supplementary Figs 7–9, Supplementary Tables 8 and 9). Results show that 60–80% of each of the mutation groups best align with the symptom progression pattern for their own mutation group supporting mutation-specific analyses. This is further supported by Supplementary Figs 3 and 4, which show the subtypes are well-differentiated, as the probability of belonging to the maximum likelihood subtype for individuals at each stage is high and increases with increasing SuStaIn stage.

### C9orf72

The data supported three subtypes under cross-validation in this genetic group (see ‘Materials and methods’ section and Supplementary Figs 1 and 2). The Ordinal SuStaIn PVD for subtype 1 (Fig. 1, Supplementary Fig. 5) shows early deficits in behaviour (irritability/lability, disinhibition, apathy, loss of sympathy/empathy) and cognition (memory impairment, impaired attention/concentration, impaired judgement/problem-solving). This is followed by worsening of behaviour and cognition/function domain symptoms at a similar stage, particularly of executive tasks such as judgement and problem-solving and problems with community affairs, which are the first symptoms to reach a severe level at stage 77 (in black on PVD in Fig. 1). Hallucinations and delusions also develop early on causing mild symptoms when many behaviour symptoms have become moderately severe. A motor domain cluster emerges once behaviour, neuropsychiatric and cognition/function domains have progressed. This combination most fits with symptoms that meet diagnostic criteria initially for bvFTD; hence, this subtype is best described as a bvFTD variant of *C9orf72*.

PVD for subtype 2 (Fig. 2, Supplementary Fig. 5) shows early bulbar motor symptoms (dysarthria and dysphagia) as well as other speech deficits (impaired articulation, decreased fluency) along with impaired word retrieval and dyslexia in the language domain, irritability/lability in the behaviour domain and impaired attention/concentration and memory difficulties in the cognition/function domain. Cognitive problems worsen with executive symptoms becoming severely affected first along with apathy by stage 87, where hallucinations emerge. This subtype is most in keeping with bulbar ALS, with later cognitive and behavioural difficulties and so this subtype is referred to as the bulbar ALS (with FTD) subtype of *C9orf72*.

PVD for subtype 3 (Fig. 3, Supplementary Fig. 5) shows a predominantly motor limb deficit, with early apathy. The plot shows early gait disorder severity, with most certainty around slowness, weakness, falls and functional difficulties using hands. There is also early irritability/lability and memory problems. This subtype describes symptoms most consistent with the classic limb variant of FTD–ALS, and so this subtype fits best as the limb ALS (with FTD) subtype of *C9orf72*.

A review of known clinical diagnoses for symptomatic cases in these subtypes showed 88% of *C9orf72* subtype 1 had bvFTD; within subtype 2 with bulbar ALS predominance, 63% had bvFTD, 11% FTD–ALS, 11% ALS and 11% PPA; and subtype 3 with mostly limb ALS features had 36% with a diagnosis of ALS, 27% FTD–ALS, 27% bvFTD and 5% PPA. Demographic and clinical score data are highlighted in Supplementary Table 3.

### GRN

The data supported two *GRN* subtypes under cross-validation (Supplementary Fig. 1). PVD for subtype 1 (Fig. 4, Supplementary Fig. 5) shows early behaviour (irritability/lability, apathy, loss of sympathy/empathy, ritualistic/compulsive behaviour, hyperorality and appetite changes) and cognition/function (memory impairment, impaired judgement/problem-solving, problems with community affairs, impaired attention/concentration), and word retrieval impairment. Neuropsychiatric features emerge once most behaviour symptoms reach moderate severity at stage 62 (in blue on PVD in Fig. 4). Problems with community affairs and problems at home or with hobbies are the first symptoms to reach a severe level at stage 84, when mild motor limb involvement (slowness, gait disorder) emerges. This pattern of symptoms is most in keeping with a behavioural phenotype with later parkinsonian features, and so this subtype is most identifiable as the bvFTD–parkinsonism subtype of *GRN*.

PVD for subtype 2 (Fig. 5, Supplementary Fig. 5) shows early language domain predominance (impaired word retrieval, impaired articulation, decreased fluency, impaired grammar/syntax, impaired sentence comprehension, dyslexia, dysgraphia, impaired functional communication) with the first symptoms to become severe in this domain (decreased fluency, impaired word retrieval, impaired functional communication) from stage 56. There is also early impaired memory and attention/concentration, apathy and altered sense of humour. Motor involvement occurs later starting with dysarthria when language domain symptoms become moderately severe. This subtype includes predominantly linguistic symptoms and is best described as the PPA variant of *GRN*.

Clinical diagnoses of *GRN* subtype 1 with bvFTD–parkinsonism predominance included 75% with bvFTD, 3% PD, 3% PSP, 3% CBS, 6% PPA, 6% bipolar and 3% Ad. About 83% of subtype 2 had a diagnosis of PPA, and 17% were diagnosed with bvFTD.

### MAPT

The data supported one symptom progression profile under cross-validation in this group [Fig. 6, Supplementary Figs 3 and 5, Supplementary Appendix (Supplementary Fig. 9)]. This group has early semantic and memory deficits (where memory is the first symptom to become severely affected) with behavioural impairment and parkinsonism (bradyphrenia, slowness, gait disorder), features consistent with previously described symptoms in *MAPT* mutation carriers. This semantic-behavioural-parkinsonism symptom profile had 87% with a diagnosis of bvFTD, 3% PSP and 3% PPA.

A summary of the subtypes discovered is listed in Supplementary Table 2 with demographic and clinical score data of the multiple genetic subtypes shown in Supplementary Table 3. Supplementary Table 4 displays the number of individuals within a genetic subtype, at a particular SuStaIn stage.

### Correlational analysis

SuStaIn Stage significantly positively correlated with the CDR® plus NACC FTLD-SOB and CDR® plus NACC FTLD-NM-SOB in the mutation carriers (all with *P* < 0.001) as a whole (*rho* = 0.944, *rho* = 0.956), within the individual genetic groups: *C9orf72* (*rho* = 0.944, *rho* = 0.963), *GRN* (*rho* = 0.937, *rho* = 0.942) and *MAPT* (*rho* = 0.960, *rho* = 0.957), and within the multiple subtypes: *C9orf72* subtype 1 (*rho* = 0.959, *rho* = 0.967), 2 (*rho* = 0.961, *rho* = 0.966), 3 (*rho* = 0.906, *rho* = 0.928); *GRN* subtype 1 (*rho* = 0.956, *rho* = 0.960), 2 (*rho* = 0.925, *rho* = 0.933) (Supplementary Table 5).

SuStaIn Stage significantly negatively correlated with FRS (all *P* < 0.001 apart from *C9orf72* subtype 3 with *P* = 0.012) in all mutation carriers (*rho* = −0.795), within the mutation groups: *C9orf72* (*rho* = −0.741), *GRN* (*rho* = −0.820) and *MAPT* (*rho* = −0.846), and within the multiple subtypes: *C9orf72* subtype 1 (*rho* = −0.787), 2 (*rho* = −0.781), 3 (*rho* = −0.562); *GRN* subtype 1 (*rho* = −0.863), 2 (*rho* = −0.764).

SuStaIn Stage significantly negatively correlated with ALSFRS-R in all mutation carriers (*rho* = −0.515, *P* < 0.001), in the *C9orf72* (*rho* = −0.589, *P* < 0.001) group, and within the multiple subtypes *C9orf72* subtype 1 (*rho* = −0.794, *P* < 0.001) and 3 (*rho* = −0.710, *P* = 0.010). There was no significant correlation in *GRN* (*rho* = 0.084, *P* = 0.795), *MAPT* (*rho* = −0.612, *P* = 0.144), *C9orf72* subtype 2 (*rho* = −0.180, *P* = 0.521), *GRN* subtype 1 (*rho* = 0.377, *P* = 0.462) and subtype 2 (*rho* = −0.371, *P* = 0.469) groups, although numbers were small in these groups (*n* = 12, 7, 15, 6, 6 and 7, respectively).

SuStaIn Stage significantly positively correlated with EYO (all *P* < 0.001 apart from *C9orf72* subtype 3 with *P* = 0.068 and *GRN* subtype 1 with *P* = 0.004) in all mutation carriers (*rho* = 0.523), within the mutation groups: *C9orf72* (*rho* = 0.488), *GRN* (*rho* = 0.429) and *MAPT* (*rho* = 0.728), and within the multiple subtypes: *C9orf72* subtype 1 (*rho* = 0.517), subtype 2 (*rho* = 0.393); *GRN* subtype 1 (*rho* = 0.395), subtype 2 (*rho* = 0.522), although not in the *C9orf72* limb ALS subtype (*rho* = 0.397). This is displayed graphically in Supplementary Fig. 6.

### Comparison between disease severity, stage and subtype

Supplementary Table 6 shows the relationship between symptom severity scores and SuStaIn subtype and stage across all subtypable mutation carriers, within a genetic group (including *C9orf72* and *GRN* groups which had more than one subtype to allow for a comparison), accounting for age, sex and education level. All symptoms significantly worsened with SuStaIn stage apart from hypersexuality in the *C9orf72* subtypes (*P* = 0.336), with borderline significance for weakness (*P* = 0.055), in the *GRN* subtypes, irritability/lability (*P* = 0.082) and hyperreligiosity (*P* = 0.086). This suggests the severity of these symptoms is unrelated to SuStaIn stage and that they do not show decline with disease progression in these groups.

Compared to *C9orf72* subtypes 2 and 3, subtype 1 with bvFTD features displayed the most affected score for all the behaviour and neuropsychiatric domain symptoms, impaired sentence and single-word comprehension within the language domain and impaired personal care, topographical memory and person recognition difficulty in the cognition/function domain. Compared to *C9orf72* subtypes 1 and 3, subtype 2 with bulbar ALS features displayed the most affected score for all the remaining language symptoms, and within the cognitive domain, impaired memory, judgement and problem solving, problems with community affairs, problems at home or with hobbies, visuospatial or perceptual impairment, impaired attention/concentration and bradyphrenia. Compared to *C9orf72* subtypes 1 and 2, subtype3 with limb ALS features displayed the most affected score for all the motor symptoms and impaired orientation within the cognitive domain.

Compared to *GRN* subtype 2, subtype 1 with bvFTD–parkinsonism displayed the most affected score for all the behaviour and neuropsychiatric domain symptoms apart from altered sense of humour, all the cognition/function symptoms and dysphagia, tremor, slowness, weakness and functional difficulties using hands in the motor domain. Compared to *GRN* subtype 1, subtype2 most in keeping with a PPA variant displayed the most affected score for all the language symptoms, altered sense of humour, dysarthria, gait disorder and falls.

### Longitudinal validation

A total of 379 (61%) mutation carriers within the cross-sectional cohort had follow-up. There was a total of 799 follow-up visits, including: 162 *C9orf72* (87 had two, 36 had three, 16 had four and 7 had five follow-up visits), 146 *GRN* (94 had two, 57 had three, 28 had four and 7 had five follow-up visits) and 71 *MAPT* (53 had two, 22 had three, 11 had four and 2 had five follow-up visits) mutation carriers. Within the mutation subtypes, at the baseline visit, 86 *C9orf72* mutation carriers were not subtypable, i.e. subtype 0 with no or few symptoms, 54 were *C9orf72* subtype 1, 13 were *C9orf72* subtype 2 and 9 were *C9orf72* subtype 3. A total of 106 *GRN* mutation carriers were not subtypable, 23 were *GRN* subtype 1 and 17 were *GRN* subtype 2 at the baseline visit. Figure 7 highlights the relationship between the subtypes and SuStaIn stage longitudinally, for up to three visits, in all groups displaying an overall trend for the SuStaIn stage to increase at follow-up.

**Figure 7:**
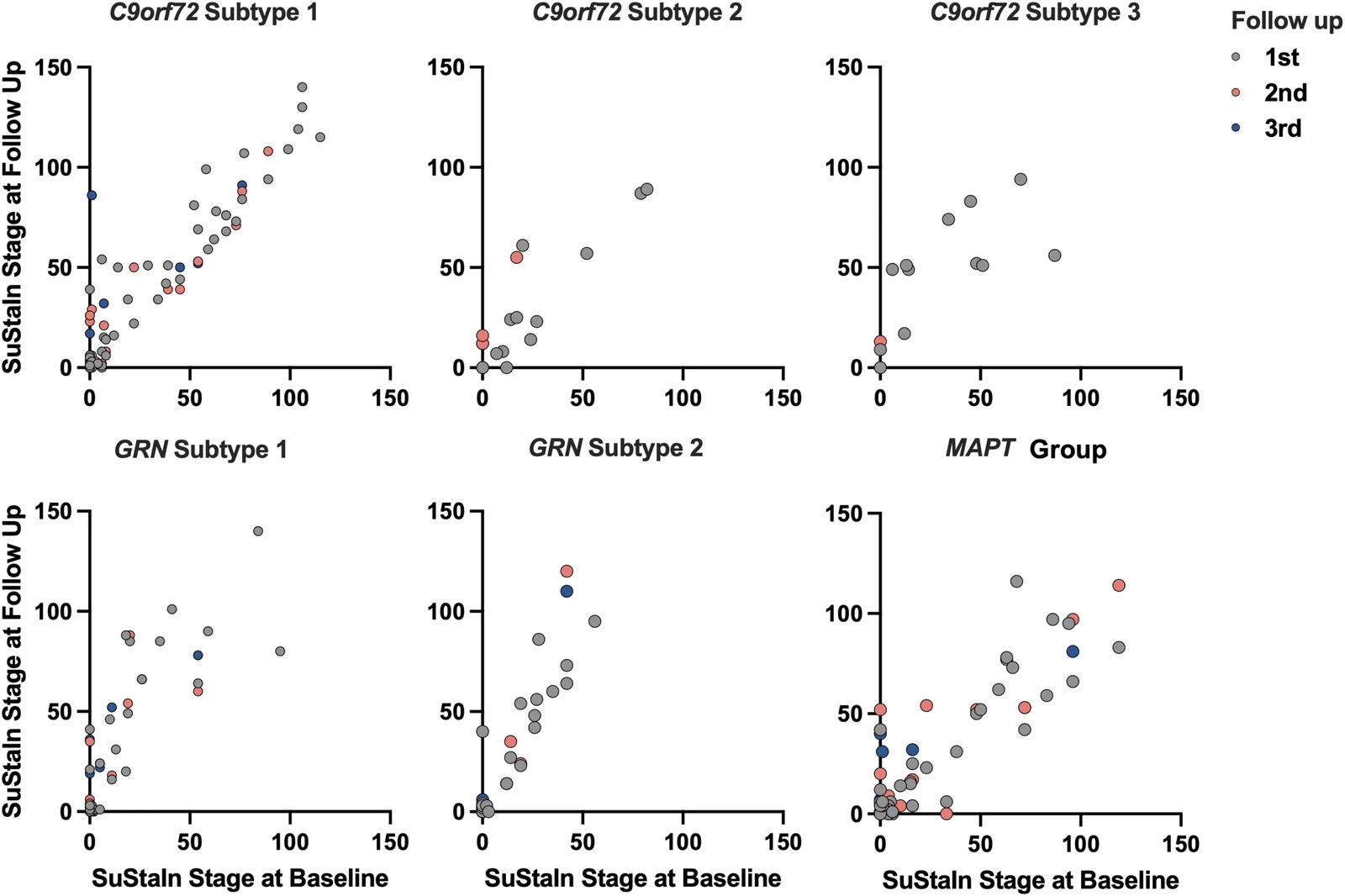
Comparison between SuStaIn stage at the baseline visit and at up to three follow-up visits in each of the genetic subtypes. *C9orf72* subtype 1—bvFTD-like (*N* = 88); *C9orf72* subtype 2—bulbar ALS-like (*N* = 35); *C9orf72* subtype 3—limb ALS-like (*N* = 22); *GRN* subtype 1—bvFTD–parkinsonism-like (*N* = 52); *GRN* subtype 2—PPA-like (*N* = 37); *MAPT* group—semantic-behavioural-parkinsonism (*N* = 49). The SuStaIn stage after an individual switches subtype is not plotted, although the SuStaIn stage for a subtype an individual switched to is plotted i.e. for a subtype's plot, an individual's allocated stage at the point they switched to this subtype from another (non-zero) subtype or their stage before switching to another subtype, is recorded.

Table 2 shows the percentage of individuals within a particular subtype that switched at a single follow-up visit for *C9orf72* and *GRN* subtypes. For *C9orf72* subtype 0, 81% were not subtypable at the baseline or follow-up visit, 17% progressed to subtype 1% and 1% progressed to subtype 3. For those who were *C9orf72* subtype 1 with bvFTD predominance at the baseline visit, 65% remained as subtype 1, 11% became subtype 0, 19% switched to the bulbar ALS subtype 2 and 6% switched to limb ALS subtype 3. For *C9orf72* subtype 2 individuals at the baseline visit, 46% remained as the same subtype, 8% became subtype 0, 39% switched to subtype 1% and 8% became subtype 3. For *C9orf72* subtype 3 individuals with mostly limb ALS features at baseline, 89% remained as this subtype at follow-up and 11% switched to subtype 1 with mostly bvFTD features. For *GRN* subtype 0, 85% were not subtypable at the baseline or follow-up visit, 8% progressed to subtype 1 characterized by bvFTD–parkinsonism and 8% progressed to subtype 2. For those who were *GRN* subtype 1 at the baseline visit, 78% remained as this subtype, 13% became subtype 0% and 9% switched to a subtype 2. For individuals who were *GRN* subtype 2 at the baseline visit, 77% remained as this subtype, 6% became subtype 0% and 18% switched to subtype 1.

**Table 2:** Longitudinal consistency of multiple genetic mutation subtype assignments over a single visit in the *C9orf72* subtypes and the *GRN* subtypes. Subtype 0 represents *C9orf72* or *GRN* mutation carriers with no or few symptoms who were therefore not subtypable

	Subtype at baseline visit	Subtype at follow-up visit			
		Subtype 0	Subtype 1 (bvFTD-like))	Subtype 2 (bulbar ALS-like)	Subtype 3 (limb ALS-like)
C9orf72	Subtype 0	70 (81.4%)	15 (17.4%)	0 (0%)	1 (1.2%)
	Subtype 1	6 (11.1%)	35 (64.8%)	10 (18.5%)	3 (5.6%)
	Subtype 2	1 (7.7%)	5 (38.5%)	6 (46.2%)	1 (7.7%)
	Subtype 3	0 (0%)	1 (11.1%)	0 (0%)	8 (88.9%)

	Subtype at baseline visit	Subtype at follow-up visit		
		Subtype 0	Subtype 1 (bvFTD–parkinsonism-like)	Subtype 2 (PPA-like)
GRN	Subtype 1	90 (84.9%)	8 (7.5%)	8 (7.5%)
	Subtype 2	3 (13.0%)	18 (78.3%)	2 (8.7%)
	Subtype 3	1 (5.9%)	3 (17.6%)	13 (76.5%)

The number of visits is shown with the percentage of the total participants within a particular subtype at the baseline visit that were in a particular subtype at the follow-up visit. For instance, *C9orf72* mutation carriers at subtype 0 at the baseline visit can remain as subtype 0 or become subtype 1, 2 or 3 at the follow-up visit, with the percentage of participants from the original subtype 0 group that remained the same or switched to one of these subtypes shown in parentheses.

## Discussion

In this study, the Ordinal SuStaIn algorithm identified FTD mutation-specific phenotypes including bvFTD, bulbar ALS with FTD and limb ALS with FTD variants of *C9orf72*; bvFTD–parkinsonism and PPA variants of *GRN*; and a semantic-behavioural-parkinsonism variant of *MAPT*. Improved understanding of early symptomatology and its expected trajectory in mutation groups aims to streamline diagnostic assessment and to optimize the targeting of therapeutic interventions to maximize clinical benefit, as well as inform trial outcomes. Developing clinical subtypes guides the prediction of disease progression making it easier to stratify individuals in clinical trials.

### C9orf72

Most *C9orf72* mutation carriers present with bvFTD or ALS,^25,26^ as exemplified by the subtypes shown in this study. Early, severe executive dysfunction was a common feature of two of the subtypes (subtype 1 with bvFTD and subtype 2 with bulbar ALS features) though occurred earlier in subtype 1. This is in line with recent work on the temporal ordering of deficits in genetic FTD^9^ and findings in asymptomatic and prodromal *C9orf72* groups within the GENFI cohort.^6^ Furthermore, executive dysfunction is known to be the main cognitive deficit that occurs in bvFTD and is described as such in consensus criteria.^27^ The identification of bulbar and limb ALS subtype within *C9orf72* mutation carriers coincides with it being the commonest genetic cause of familial and sporadic ALS.^25,28-30^ Moreover, of the three main mutations, *C9orf72* has been found to harbour the most ALS-like features,^8^ and many people develop an FTD–ALS overlap syndrome.^30-35^

#### Subtype 1

This phenotype showed worse scores in behaviour, neuropsychiatric and cognitive domains expected to be affected in bvFTD, with additional language comprehension impairment. This is supported by previous work that suggests secondary language deficits occur in those with bvFTD.^7^ BvFTD is also the most frequent phenotype observed in *C9orf72* mutation carriers.^30,36-39^ The development of hallucinations and delusions when behaviour becomes moderately affected fits with findings in other studies that these symptoms become severe in intermediate and late phases of disease in *C9orf72* mutation carriers.^40,41^ Early memory and attentional deficits have also been shown in *C9orf72* bvFTD groups.^7^ Prior studies found nearly 20% of its bvFTD cohort eventually developed definite ALS^42,43^; *C9orf72* subtype 1 also develops motor features later on.

#### Subtype 2

Language was particularly affected in this group, namely early difficulties with articulation, dysfluency and dyslexia likely related to motor speech deficits, with bulbar symptoms, dysarthria and dysphagia also affected. Bulbar ALS occurs in around a quarter of all Motor Neuron Disease (MND) and is associated with cognitive and behavioural disturbance seen in FTD.^44^ Individuals with language-dominant FTD–ALS, predominantly of the nonfluent type, are shown to have bulbar rather than limb-onset FTD–ALS.^35,45,46^ Around half of individuals with ALS develop behavioural (in this subtype, early irritability/lability) and cognitive impairment (here, memory, executive function and attentional/speed of processing deficits).^47^ It is also known that cognitive and behavioural issues occur more frequently (nearly half of cases in some reports) in *C9orf72* mutation carriers with ALS than in those with sporadic or other genetic causes of ALS.^48,49^ Hallucinations developed when executive function became severely affected. A study has shown neuropsychiatric features are more common in *C9orf72* mutation carriers with FTD–ALS, who tend to have more bulbar presentations than individuals with FTD–ALS without this mutation.^50^

#### Subtype 3

This group had worse scores for all the motor symptoms. Cohort studies suggest limb onset FTD-ALS in *C9orf72* mutation carriers is more common than the bulbar form.^33,35^ However other studies suggest the inverse is true.^32,34,50-52^ This subtype is characterized by early apathy and early progression of limb motor symptoms, supported by a similar phenotype described in the literature.^53^ One large cohort study showed apathy in 80% of its ALS patients,^54^ whilst another showed early apathy as more a feature of non-*C9orf72* mutation carriers with FTD–ALS, although found apathy does occur in *C9orf72* as the disease progresses. However, this study was poorly powered and included only 10 participants with *C9orf72* mutations and bvFTD diagnoses.^55^

### GRN

This study found two separate subtypes for *GRN* mutation carriers, one of language-dominant symptoms, and the other of behavioural-parkinsonian features, at least in the initial stages.

#### Subtype 1

This phenotype had worse scores in the behaviour, neuropsychiatric and cognitive/function domain, supporting the ‘behavioural’ aspect of the subtype, whilst also scoring worse in the motor domain in support of the ‘parkinsonian’ part of the subtype. This group had early tremor, slowness, dysphagia, falls and functional difficulties using hands, as well as bradyphrenia, all features of parkinsonism. Interestingly, CBS, a parkinsonian disorder with limb rigidity and apraxia, alien limb phenomenon, cortical sensory loss and myoclonus, can occur in people with *GRN* mutations,^56,57^ although is rarely identified as the presenting symptom.^58-62^

Although limb motor symptoms occur early, other domains, including behaviour and cognition/function, are first affected. CBS overlap with bvFTD and nonfluent variant (nfv) PPA described in the literature^63,64^ helps explain the early behaviour component and impaired word retrieval noted in this subtype. Moreover, impaired cognition (shown in this subtype by early memory, executive and attentional deficits) and nfvPPA tend to be seen in CBS caused by *GRN* mutations.^65^ Behavioural symptoms in this subtype included early apathy and loss of sympathy/empathy. BvFTD diagnoses in *GRN* mutation carriers with CBS features have previously been described as dominated by apathy and social withdrawal,^66,67^ with apathy as the most common behavioural change in *GRN* mutation carriers in general.^68^ Furthermore, in this subtype, neuropsychiatric symptoms develop once behaviour is moderately affected. Hallucinations and delusions have been reported in a significant minority of individuals with *GRN* mutations.^69^

#### Subtype 2

The PPA variant of *GRN* is affected by an array of language domain symptoms: impaired word retrieval, decreased fluency and impaired functional communication are the first symptoms in this subtype to become severe. When language symptoms become moderately affected, dysarthria, which tends to be reported along with difficulties in articulation and dysfluency, becomes affected. Previous reports describe *GRN* mutations as the commonest genetic cause of nfvPPA or PPA–not-otherwise-specified (PPA–NOS) when language symptoms do not fit within a specific PPA group.^70-76^ A genetic PPA study^77^ found almost all had *GRN* mutations, including nfvPPA and PPA–NOS characterized by impaired grammar/syntax, decreased fluency, impaired word retrieval and sentence comprehension, features highlighted in this subtype. This prior study highlighted a further commonality: apathy, which was the most common behavioural symptom in all the *GRN–*PPA diagnoses, an early feature in this subtype, and consistent with other reports of frequent apathy in *GRN* mutation carriers.^61^

Early memory and attentional impairment in this subtype coincide with a study that showed episodic memory impairment on neuropsychometry with hippocampal atrophy on imaging for the PPA–NOS *GRN* subgroup, and for all the *GRN–*PPA individuals studied, neuropsychometry highlighted attentional deficits.^77^ Parietal cortex and basal ganglia atrophy in *GRN–*FTD offers an anatomical explanation for memory impairment as well as CBS-related parkinsonism seen in *GRN* mutation carriers as a whole.^58,66,78,79^

### MAPT

Although previous studies have described two major *MAPT* phenotypes: a ‘dementia-dominant’ bvFTD type^80^; and a ‘parkinsonism-dominant’ type, occasionally with features specifically of PSP or CBS,^81,82^ this study identified only a single symptom progression profile, with semantic, behavioural and parkinsonian predominant features.

Prior GENFI studies have shown *MAPT* mutation carriers perform worse than other mutation groups on neuropsychometry that assesses memory, semantic knowledge (on a naming task) and social cognition.^3,83^ Semantic impairment is known to occur in *MAPT* mutation carriers and is highlighted in previous studies,^84-87^ although often a bvFTD diagnosis with secondary semantic impairment.^88-90^ Semantic impairment is known in *MAPT* mutation carriers,^84^ and impaired memory has led to misdiagnosis with Ad.^91^ Neuroanatomical studies showing anteromedial temporal volume loss also support semantic-memory deficits in *MAPT* groups.^3,4,92-95^ Focal temporal atrophy, an area known to cause memory loss when affected, has been shown specifically in presymptomatic and symptomatic *MAPT* mutation carriers,^6,7^ where one study indicates medial-temporal lobe volume loss occurs around 15 years prior to disease onset.^9^ Impaired word retrieval in this group, with an emergence of sentence comprehension difficulty, both of which are semantic knowledge functions, become more severely affected at later stages, while behaviour and cognition/function domains including memory appear to deteriorate sooner.

Parkinsonian features in *MAPT* mutation carriers are well-described^81,82,96^ and may be consistent with PSP and CBS.^80,81,97-99^

### Symptoms with no significant change with SuStaIn stage

Overall, subtypes matched expected characteristics extracted from symptom severity scores as discussed. The symptoms that did not show disease progression with SuStaIn stage (hypersexuality and weakness in *C9orf72*, and irritability/lability and hyperreligiosity in *GRN*) may relate to small numbers affected so results are less interpretable. It may be that certain symptoms (hypersexuality, hyperreligiosity and irritability/lability), once present, are difficult for individuals to quantify as worsening. It is also possible that as the disease progresses, patients lose insight or become less concerned by psychosis-type symptoms, and so report less impairment as it bears less significance to their and/or their caregiver’s lives. Severity scores for these symptoms, therefore, have a less clear disease progression pattern.

### Correlational analysis

CDR scales measure disease severity, so an advanced stage is suggestive of FTD severity. Significant correlations were found in all the mutation groups for SuStaIn stage and CDR scales, with a nominal increase in significance in the CDR® plus NACC FTLD-NM compared with the original CDR® plus NACC FTLD for *C9orf72* and *GRN* groups. This suggests FTD disease staging is improved with the CDR® plus NACC FTLD-NM scales as neuropsychiatric and motor elements of the disease are accounted for that are not in the original CDR® plus NACC FTLD scale.^13^

The FRS also showed significant correlations with SuStaIn Stage within all the genetic groups. Non-significant correlations of the ALSFRS-R with SuStaIn stage occurred in the *GRN* and *MAPT* groups, suggesting this scale does better to assess deficits that occur in *C9orf72* mutation carriers where the correlation was significant, and so better predicts disease staging overall.

### Longitudinal validation

Longitudinal analysis of the change in disease severity scores and phenotypic patterns within each of the subtypes allows for greater understanding of how the subtypes progress. Longitudinal consistency is achieved when the subtype allocated at the baseline visit remains unchanged, or when a subtype 0 mutation carrier, that initially had no or minimal symptoms, progresses to that subtype.

For the *C9orf72* groups, at a single follow-up visit, subtype 3 (with limb ALS predominance) displayed the most longitudinal consistency, followed by subtype 1 (with bvFTD predominance), with the least stability seen in the bulbar ALS predominant subtype 2. However, when observing the relationship with SuStaIn stage over a longer period of up to three visits, subtype 2 shows a clearer trend towards SuStaIn stage increasing over time than subtype 3. This, along with the scoring comparisons in relation to subtype and stage between the bulbar and limb ALS predominant groups that suggest earlier, more widespread impairment in the limb ALS predominant group, indicates more instability in subtype allocation in the early stages of FTD for subtype 2 (with bulbar ALS predominance).

Both the *GRN* groups showed similar reasonable longitudinal stability after a single visit and showed a trend towards increased SuStaIn staging at follow-up visits, supporting the presence of these two subtypes in *GRN* mutation carriers.

Overall, there was limited longitudinal stability observed in all subtypes, most likely related to the nature of symptom severity scoring which is open to subjectivity and thus vulnerable to inter- and intra-rater variability, and which requires a more objective measure to allow for more confident interpretation of the data.

### Limitations

Although this work used data from one of the largest genetic FTD cohorts, the GENFI study, once stratified into the multiple genetic subtypes, numbers were small. Though this study sheds light on the ordering of symptoms within each subtype and how they compare, it cannot tell us the duration from the development of one symptom or set of symptoms to the next. This limits the use of this information as a prognostication tool, important to individuals with FTD and their families. Use of newer disease progression models, e.g. temporal event-based^100^ or temporal SuStaIn modelling,^101^ should be a consideration for future development of this work to address this limitation. Combining this symptom rating scale data with biomarkers such as intracranial volumes and neuropsychology scores would also allow for improved output of an individualized profile and thus better targeting of therapies in trials and eventually the clinic setting.

Contrary to what is known, SuStaIn did not identify a ‘pure ALS’ subtype in the *C9orf72* group. It is possible this relates to a selection bias in the GENFI study. Efforts to work more closely with the MND research community aim to address this in future studies. We also recognize that the scales used in this study are imperfect, particularly as the questionnaire only includes reported symptoms and not signs identified on physical examination.^8,13^ Subtypes were labelled numerically and described by their predominant phenotype, as putting a single name to a progressive set of symptoms has its limitations. Although the accuracy of individual assignments to subtypes will be lower than a more robust measurement, as was shown by the longitudinal consistency, the scales should still enable the delineation of group-level patterns and therefore clinical subtypes. In the longitudinal study, subtypes were somewhat longitudinally consistent, suggesting validity of the group-level subtype patterns; however, consistency was lower than imaging models,^10^ probably due to the difficulty in establishing robust clinical measures, and so the subtype assignments may be less accurate at the individual level.

### Summary

Identifying subtypes within each of the mutation groups using Ordinal SuStaIn provides a novel approach for untangling the heterogeneity of FTD that has historically posed a barrier to recruiting suitable trial candidates. Awareness of specific subsets of FTD groups brings us closer to an individualized approach to care for patients, with individualized endpoints in therapeutic trials. Disease progression modelling such as used in this study can help to identify the likely progression of further symptoms within a particular phenotype but at present we are not able to identify the exact timeline; future studies using temporal-based disease progression modelling will further advance this knowledge.

## Supplementary Material

fcag392_Supplementary_Data

## Data Availability

Data are available upon reasonable request.

## Appendix List of GENFI consortium members

Rhian Convery, Martina Bocchetta, David Cash, Sophie Goldsmith, David L. Thomas, Thomas Cope, Timothy Rittman, Maura Malpetti, Antonella Alberici, Enrico Premi, Roberto Gasparotti, Emanuele Buratti, Valentina Cantoni, Andrea Arighi, Chiara Fenoglio, Vittoria Borracci, Maria Serpente, Tiziana Carandini, Emanuela Rotondo, Giacomina Rossi, Giorgio Giaccone, Giuseppe Di Fede, Paola Caroppo, Sara Prioni, Veronica Redaelli, David Tang-Wai, Ekaterina Rogaeva, Miguel Castelo-Branco, Morris Freedman, Ron Keren, Sandra Black, Sara Mitchell, Christen Shoesmith, Robart Bartha, Rosa Rademakers, Jackie Poos, Janne M. Papma, Lucia Giannini, Liset de Boer, Julie de Houwer, Rick van Minkelen, Yolande Pijnenburg, Benedetta Nacmias, Camilla Ferrari, Cristina Polito, Gemma Lombardi, Valentina Bessi, Mattias Nilsson, Henrik Viklund, Melissa Taheri Rydell, Vesna Jelic, Linn Öijerstedt, Tobias Langheinrich, Albert Lladó, Anna Antonell, Jaume Olives, Mircea Balasa, Nuria Bargalló, Sergi Borrego-Ecija, Ana Verdelho, Carolina Maruta, Tiago Coelho, Gabriel Miltenberger, Frederico Simões do Couto, Alazne Gabilondo, Ioana Croitoru, Mikel Tainta, Myriam Barandiaran, Patricia Alves, Benjamin Bender, David Mengel, Lisa Graf, Annick Vogels, Mathieu Vandenbulcke, Philip Van Damme, Rose Bruffaerts, Koen Poesen, Pedro Rosa-Neto, Maxime Montembault, Agnès Camuzat, Alexis Brice, Anne Bertrand, Aurélie Funkiewiez, Daisy Rinaldi, Dario Saracino, Olivier Colliot, Sabrina Sayah, Catharina Prix, Elisabeth Wlasich, Olivia Wagemann, Sonja Schönecker, Alexander Maximilian Bernhardt, Anna Stockbauer, Jolina Lombardi, Sarah Anderl-Straub, Adeline Rollin, Gregory Kuchcinski, Maxime Bertoux, Thibaud Lebouvier, Vincent Deramecourt, João Durães, Marisa Lima, Maria João Leitão, Maria Rosario Almeida, Miguel Tábuas-Pereira, Sónia Afonso and João Lemos.
