# Supplementary material for: Distinct clinical subtypes of genetic frontotemporal dementia identified using disease progression modelling
Source: Brain Commun. 2026 Oct 15;8(6):fcag392. doi: 10.1093/braincomms/fcag392 (PMC13766679; doi:10.1093/braincomms/fcag392)
Supplement: Supplementary file 1 [file fcag392_supplementary_data.pdf]

Supplementary Table 1 Other measures of FTD severity for the mutation groups and controls. Data are shown as mean (standard deviation). Bold items are significantly different to controls; [a]significantly severe compared to *MAPT* mutation carriers; [b]significantly severe compared to *GRN* mutation carriers. Mini-Mental State Examination (MMSE), Progressive Aphasia Severity Scale Sum of Boxes (PASS SOB, 0=no impairment), Amyotrophic lateral sclerosis Functional Rating Scale-Revised (ALSFRS-R, 48=no impairment), Cambridge Behavioural Inventory-Revised (CBI-R, 0=no impairment). MMSE was performed on 339 controls and 605 mutation carriers (including 275 *C9orf72*, 233 *GRN*, 97 *MAPT* mutation carriers). ALSFRS-R was performed on 57 controls and 154 mutation carriers (101 *C9orf72*, 31 *GRN*, 22 *MAPT* mutation carriers). CBI-R was performed on 297 controls and 548 mutation carriers (242 *C9orf72*, 219 *GRN*, 87 *MAPT* mutation carriers).

	**Controls**	***C9orf72***	***GRN***	***MAPT***
**MMSE**	29.3 (1.0)	**27.0 (4.9)**	**26.7 (6.3)**	**27.4 (4.9)**
**PASS SOB**	0.1 (0.4)	**1.5 (3.6)**	**2.1 (5.3)[a]**	**1.2 (2.9)**
**ALSFRS-R**	47.8 (0.5)	**40.7 (13.1)[a]**	**41.8 (12.9)[a]**	47.6 (1.7)
**CBI-R**	4.8 (6.9)	**29.5 (35.0)[b]**	**19.1 (29.9)**	**25.9 (32.4)[b]**

Supplementary Table 2 Summary of mutation-specific symptom progression profiles within the study

		Predominant diagnosis	Description
	Subtype 0	**Not subtypable**	Mutation carriers at stage 0 with no/few symptoms, therefore not subtypable.
C9orf72	Subtype 1	**Behavioural variant (bv) FTD**	Early behaviour and cognition/function deficits. Early hallucinations and delusions. Later motor deficits.
	Subtype 2	**Bulbar ALS**	Early bulbar and language deficits. Irritability/lability and cognition/function deficits. Later apathy and hallucinations.
	Subtype 3	**Limb ALS**	Early motor limb deficits and apathy.
GRN	Subtype 1	**BvFTD-parkinsonism**	Early behaviour and cognition/function deficits. Early motor limb and word retrieval impairment. Later neuropsychiatric symptoms.
	Subtype 2	**Primary Progressive Aphasia (PPA)**	Early language impairment. Early cognition/function deficits and apathy. Later dysarthria followed by other motor deficits.
MAPT		**Semantic-behavioural-parkinsonism**	Early semantic and memory deficits. Behaviour, bradyphrenia and motor limb deficits.

Supplementary Table 3 Demographics and clinical scores for the multiple genetic subtypes. Data are shown as mean (standard deviation). Bold items are significantly different to controls; [a]significantly more males/severe/ fewer 'Expected Years to Onset' (EYO) compared to *GRN* subtype 2; [b]significantly young/severe/fewer education years/fewer EYO compared to *C9orf72* subtype 3; [c]significantly old/severe/fewer EYO compared to *MAPT*; [d]significantly severe/more EYO compared to *C9orf72* subtype 1; [e]significantly severe compared to *GRN* subtype 1; [f]significantly severe compared to *C9orf72* subtype 2 mutation carriers.

	C9orf72			***GRN***		***MAPT***
**Subtype:**	**1**	**2**	**3**	**1**	**2**	**-**
**Number of participants**	88	35	22	52	37	49
**% Male**	**62.5[a]**	54.3	59.1	48.1	37.8	51.0
**Age in years**	**57.3 (12.4)[bc]**	**59.1 (12.9)[c]**	**62.1 (7.8)[c]**	**59.1 (12.1)[c]**	**59.1 (12.2)[c]**	**50.2 (13.2)**
**Expected years to onset**	**-0.7 (11.3)[c]**	**-0.3 (10.3)[c]**	**2.6 (5.7)**	**-1.8 (11.3)[d]**	**-2.4 (12.1)[d]**	**-1.8 (11.4)[abd]**
**Education in years**	**13.4 (3.0)**	**12.0 (4.0)[b]**	13.5 (3.6)	**12.5 (3.5)**	**12.4 (3.7)**	13.9 (3.2)
**Mini-Mental State Examination**	**26.2 (4.8)**	**22.2 (7.2)[d]**	**23.8 (6.7)**	**23.7 (6.3)**	**19.0 (10.1)[bcde]**	**25.5 (6.3)[d]**
**CDR® plus NACC FTLD-Global Score**	**1.5 (1.0)**	**1.6 (1.0)**	**1.6 (1.2)**	**1.5 (1.0)[c]**	**1.4 (1.0)**	**1.4 (1.0)**
**CDR® plus NACC FTLD-Sum of Boxes**	**7.3 (6.4)**	**8.2 (6.6)**	**8.7 (7.7)**	**7.6 (6.7)[c]**	**6.8 (6.2)**	**6.9 (6.3)**
**CDR® plus NACC FTLD-NM-Global Score**	**1.5 (1.0)**	**1.6 (1.0)**	**1.9 (0.9)**	**1.5 (1.0)[c]**	**1.4 (1.0)**	**1.4 (1.0)**
**CDR® plus NACC FTLD-NM-Sum of Boxes**	**8.0 (7.1)**	**8.9 (7.0)**	**10.7 (8.3)**	**8.2 (7.2)[c]**	**7.3 (6.8)**	**7.2 (6.5)**
**FTD Rating Scale**	**50.7 (32.0)[a]**	**49.1 (31.5)**	**46.6 (38.1)**	**56.0 (34.0)**	**58.6 (29.1)**	**58.0 (31.1)**
**Progressive Aphasia Severity Scale Sum of Boxes**	**2.3 (4.7)**	**3.6 (4.0)[de]**	**3.8 (5.5)**	**2.4 (4.0)**	**10.0 (9.0)[bcdef]**	**2.6 (3.7)[de]**
**ALS Functional Rating Scale-Revised**	**39.6 (13.0)**	**31.6 (19.6)[c]**	**29.8 (13.7)[cd]**	34.7 (18.5)	**32.2 (19.0)[cd]**	46.9 (3.0)
**Cambridge Behavioural Inventory-Revised**	**50.8 (34.6)[a]**	**50.2 (32.3)**	**61.8 (42.8)**	**48.9 (38.2)**	**41.2 (29.8)**	**44.6 (36.5)**

Supplementary Table 4 displays the number of individuals within a genetic subtype (i) *C9orf72* subtype1, 2 and 3, (ii) *GRN* subtype 1 and 2, and (iii) the *MAPT* subtype, at a particular SuStain stage. Subtype 0 are a group that remained at SuStaIn stage 0.

(i)

**Stage**	***C9orf72* subtype**			
	**0**	**1**	**2**	**3**
0	135	0	0	0
1	0	16	0	0
2	0	2	1	0
3	0	4	0	0
4	0	1	3	0
5	0	1	1	0
6	0	5	0	1
7	0	1	2	0
8	0	2	0	0
10	0	0	1	1
12	0	1	1	1
13	0	1	0	1
14	0	1	2	1
15	0	0	0	2
17	0	1	1	0
19	0	2	0	0
20	0	0	1	1
22	0	1	2	0
24	0	0	1	0
26	0	1	0	0
27	0	0	1	0
29	0	3	0	0
30	0	1	0	0
33	0	1	0	0
34	0	1	0	1
38	0	1	1	0
39	0	1	0	0
41	0	0	1	0
42	0	0	0	1
45	0	2	0	1
48	0	1	0	0
50	0	2	0	0
51	0	2	0	1

52	0	1	1	0
53	0	1	1	0
54	0	1	0	0
56	0	2	0	1
57	0	0	1	0
58	0	1	0	0
59	0	1	1	0
62	0	2	0	0
63	0	2	1	0
64	0	0	1	0
65	0	1	0	0
67	0	0	2	0
68	0	2	0	1
69	0	1	1	0
70	0	1	0	0
71	0	0	0	1
72	0	0	1	0
73	0	1	0	0
75	0	0	0	1
76	0	2	0	0
77	0	2	0	0
79	0	0	1	0
81	0	0	0	1
82	0	1	1	0
87	0	1	2	1
89	0	1	0	0
93	0	1	0	0
96	0	1	0	0
99	0	1	0	0
101	0	0	0	1
104	0	1	0	0
106	0	2	0	0
109	0	0	1	0
111	0	0	0	1
115	0	1	0	0
123	0	1	0	0
124	0	0	0	1
128	0	0	1	0

141	0	1	0	0
143	0	0	0	1

(ii)

**Stage**	***GRN* subtype**		
	**0**	**1**	**2**
0	153	0	0
1	0	7	0
2	0	3	5
3	0	0	2
5	0	4	0
10	0	2	0
11	0	2	1
12	0	0	1
13	0	1	0
14	0	1	2
15	0	1	0
17	0	0	1
18	0	2	0
19	0	1	2
20	0	1	0
21	0	0	1
22	0	1	0
26	0	1	2
27	0	0	1
28	0	0	1
31	0	1	0
33	0	2	0
35	0	2	1
40	0	0	1
41	0	1	0
42	0	0	2
43	0	0	1
46	0	1	0
47	0	0	1
49	0	1	0
51	0	1	1

53	0	0	1
54	0	1	0
56	0	0	1
59	0	1	0
61	0	0	1
62	0	2	1
63	0	0	1
65	0	1	0
68	0	1	0
69	0	1	1
77	0	1	0
78	0	0	1
84	0	1	0
85	0	1	0
89	0	0	1
90	0	1	0
95	0	1	0
96	0	0	1
97	0	1	0
102	0	1	0
108	0	0	1
110	0	1	0
137	0	1	0
138	0	0	1

(iii)

**Stage**	***MAPT* subtype**	
	**0**	**1**
0	52	0
1	0	8
4	0	3
5	0	2
6	0	4
10	0	1
15	0	1
16	0	2
17	0	1
18	0	1

20	0	1
23	0	1
27	0	2
32	0	1
33	0	1
38	0	1
39	0	2
47	0	1
48	0	1
50	0	1
53	0	1
57	0	1
59	0	1
63	0	1
66	0	1
68	0	1
72	0	1
83	0	1
86	0	1
90	0	1
94	0	1
96	0	1
104	0	1
119	0	1

Supplementary Table 5 Correlations between SuStaIn Stage and FTD severity markers in the combined mutation carrier group, within genetic groups, and within the multiple genetic subtypes (*C9orf72* subtype 1 – bvFTD-like; *C9orf72* subtype 2 - bulbar ALS-like; *C9orf72* subtype 3 - limb ALS-like; *GRN* subtype 1 - bvFTD-parkinsonian-like; *GRN* subtype 2 – PPA-like; *MAPT* - semantic-behavioural-parkinsonian-like). Rho is shown for each correlation, with significant values shown in bold.

	All mutation carriers	*C9orf72*				*GRN*			*MAPT*
		All	Subtype 1	Subtype 2	Subtype 3	All	Subtype 1	Subtype 2	All
**CDR® plus NACC FTLD-SOB**	**0.944**	**0.944**	**0.959**	**0.961**	**0.906**	**0.937**	**0.956**	**0.925**	**0.960**
**CDR® plus NACC FTLD-NM-SOB**	**0.956**	**0.963**	**0.967**	**0.966**	**0.928**	**0.942**	**0.960**	**0.933**	**0.957**
**FTD Rating Scale**	**-0.795**	**-0.741**	**-0.787**	**-0.781**	**-0.562**	**-0.820**	**-0.863**	**-0.764**	**-0.846**
**Progressive Aphasia Severity Scale SOB**	**0.490**	**0.498**	**0.575**	0.296	**0.541**	**0.553**	**0.539**	**0.734**	**0.465**
**ALS FTD Rating Scale**	**-0.515**	**-0.589**	**-0.794**	-0.180	**-0.710**	0.084	0.377	-0.371	-0.612
**Cambridge Behavioural Inventory-Revised**	**0.844**	**0.828**	**0.870**	**0.844**	**0.674**	**0.801**	**0.852**	**0.770**	**0.905**
**Expected years to onset**	**0.523**	**0.488**	**0.517**	**0.393**	0.397	**0.429**	**0.395**	**0.522**	**0.728**

Supplementary Table 6 Comparison of symptom severity scores of (a) *C9orf72* and (b) *GRN* mutation carriers assigned to each Subtype and Stage Inference (SuStaIn) subtype and stage. Age, sex and education level were included as additional covariates. *C9orf72* subtype 1 (bvFTD-like), 2 (bulbar ALS-like) and 3 (limb ALS-like) are shown in (a), and *GRN* subtype 1 (bvFTD-parkinsonian-like) and 2 (PPA-like) are shown in (b). The subtype with the highest *t* value between the subtypes within a mutation group were classified as most affected by the symptom but if there was <0.5 difference in *t* value between subtypes more than one option was possible as the group with the worse score. The change with SuStaIn stage was statistically significant at *p*<0.05.

(a)

**Symptom**	**Subtype**	**SuStaIn subtype**		**Group with worse score**	**SuStaIn stage**		**Change with SuStaIn stage**
		***t* value**	***p* value**		***t* value**	***p* value**	
**Behavioural**							
**Disinhibition**	1	-0.14	0.887	1 (bvFTD-like)	7.42	<0.001	Worsens
	2	-3.05	0.003				
	3	-1.93	0.056				
**Apathy**	1	0.79	0.434	1 (bvFTD-like)	11.06	<0.001	Worsens
	2	-1.77	0.079				
	3	-1.51	0.134				
**Loss of sympathy/empathy**	1	0.77	0.445	1 (bvFTD-like)	9.76	<0.001	Worsens
	2	-3.52	0.001				
	3	-5.46	<0.001				
**Ritualistic/compulsive behaviour**	1	2.28	0.024	1 (bvFTD-like)	8.14	<0.001	Worsens
	2	-3.73	<0.001				
	3	-3.04	0.003				
**Hyperorality and appetite changes**	1	1.68	0.096	1 (bvFTD-like)	9.53	<0.001	Worsens
	2	-3.05	0.003				
	3	-1.82	0.071				
**Poor response to social/emotional cues**	1	0.34	0.736	1 (bvFTD-like)	10.23	<0.001	Worsens
	2	-3.00	0.003				
	3	-4.05	<0.001				
**Inappropriate trusting behaviour**	1	1.43	0.154	1 (bvFTD-like)	7.11	<0.001	Worsens
	2	0.91	0.366				

	**3**	-1.58	0.117				
**Neuropsychiatric**							
**Hallucinations**	**1**	2.15	0.034	1 (bvFTD-like)	3.85	<0.001	Worsens
	**2**	-2.54	0.012				
	**3**	-0.76	0.447				
**Delusions**	**1**	0.44	0.658	1 (bvFTD-like)	5.23	<0.001	Worsens
	**2**	-2.44	0.016				
	**3**	-0.38	0.701				
**Irritability/lability**	**1**	1.77	0.079	1 (bvFTD-like)	3.69	<0.001	Worsens
	**2**	0.2	0.843				
	**3**	-0.01	0.989				
**Agitation/aggression**	**1**	2.49	0.014	1 (bvFTD-like)	4.31	<0.001	Worsens
	**2**	-0.82	0.411				
	**3**	-0.81	0.419				
**Euphoria/elation**	**1**	1.31	0.191	1 (bvFTD-like)	4.22	<0.001	Worsens
	**2**	-1.42	0.159				
	**3**	-1.39	0.166				
**Aberrant motor behaviour**	**1**	2.06	0.042	1 (bvFTD-like)	7.23	<0.001	Worsens
	**2**	-2.26	0.025				
	**3**	-0.78	0.438				
**Hypersexuality**	**1**	-0.04	0.972	1 (bvFTD-like)	0.97	0.336	No significant change
	**2**	-1.78	0.077				
	**3**	-0.46	0.644				
**Hyperreligiosity**	**1**	0.40	0.691	1 (bvFTD-like)2 (bulbar ALS-like)	2.12	0.036	Worsens
	**2**	0.06	0.954				
	**3**	-1.35	0.179				
**Altered sense of humour**	**1**	0.43	0.667	1 (bvFTD-like)	5.4	<0.001	Worsens
	**2**	-1.93	0.056				
	**3**	-0.47	0.642				

**Language**							
**Impaired articulation**	**1**	-1.59	0.113	2 (bulbar ALS-like)	7.38	<0.001	Worsens
	**2**	3.84	<0.001				
	**3**	1.17	0.243				
**Decreased fluency**	**1**	-2.01	0.047	2 (bulbar ALS-like)	9.99	<0.001	Worsens
	**2**	3.05	0.003				
	**3**	2.16	0.033				
**Impaired grammar/syntax**	**1**	0.00	0.999	2 (bulbar ALS-like)	6.9	<0.001	Worsens
	**2**	1.62	0.107				
	**3**	0.02	0.984				
**Impaired word retrieval**	**1**	-0.23	0.819	2 (bulbar ALS-like) 3 (limb ALS-like)	8.44	<0.001	Worsens
	**2**	1.8	0.074				
	**3**	1.39	0.168				
**Impaired speech repetition**	**1**	0.08	0.938	2 (bulbar ALS-like) 3 (limb ALS-like)	7.2	<0.001	Worsens
	**2**	1.23	0.222				
	**3**	1.18	0.24				
**Impaired sentence comprehension**	**1**	1.31	0.192	1 (bvFTD-like)	8.21	<0.001	Worsens
	**2**	0.50	0.618				
	**3**	-0.60	0.552				
**Impaired single word comprehension**	**1**	1.25	0.214	1 (bvFTD-like)	7.02	<0.001	Worsens
	**2**	-0.17	0.866				
	**3**	-2.06	0.041				
**Dyslexia**	**1**	1.31	0.191	2 (bulbar ALS-like) 1 (bvFTD-like)	6.04	<0.001	Worsens
	**2**	1.67	0.097				
	**3**	-1.13	0.261				
**Dysgraphia**	**1**	1.18	0.240	2 (bulbar ALS-like)	8.61	<0.001	Worsens
	**2**	2.32	0.022				
	**3**	1.34	0.184				
**Impaired functional communication**	**1**	-0.65	0.515	2 (bulbar ALS-like)	9.67	<0.001	Worsens

	**2**	1.8	0.074				
	**3**	-0.91	0.364				
**Cognitive**							
**Memory impairment**	**1**	-0.64	0.524	2 (bulbar ALS-like)	11.71	<0.001	Worsens
	**2**	1.34	0.183				
	**3**	0.27	0.784				
**Impaired orientation**	**1**	-0.25	0.802	3 (limb ALS-like)2 (bulbar ALS-like)	9.95	<0.001	Worsens
	**2**	-0.42	0.676				
	**3**	-0.07	0.941				
**Impaired judgement and problem solving**	**1**	-1.35	0.178	2 (bulbar ALS-like)	18.46	<0.001	Worsens
	**2**	-0.65	0.518				
	**3**	-4.03	<0.001				
**Problems with community affairs**	**1**	-0.89	0.378	2 (bulbar ALS-like)	21.13	<0.001	Worsens
	**2**	0.31	0.758				
	**3**	-1.79	0.076				
**Problems at homes or with hobbies**	**1**	0.28	0.779	2 (bulbar ALS-like) 1 (bvFTD-like)	16.50	<0.001	Worsens
	**2**	0.66	0.512				
	**3**	-2.17	0.032				
**Impaired personal care**	**1**	0.96	0.338	1 (bvFTD-like)	12.65	<0.001	Worsens
	**2**	-1.73	0.085				
	**3**	-1.07	0.285				
**Person recognition difficulty**	**1**	-0.86	0.392	1 (bvFTD-like) 3 (limb ALS-like)	7.12	<0.001	Worsens
	**2**	-1.41	0.16				
	**3**	-1.14	0.258				
**Impaired topographical memory**	**1**	0.10	0.921	1 (bvFTD-like)	10.03	<0.001	Worsens
	**2**	-0.80	0.424				
	**3**	-1.75	0.082				
**Visuospatial or perceptual impairment**	**1**	-0.20	0.844	2 (bulbar ALS-like)	4.82	<0.001	Worsens
	**2**	2.35	0.020				

	3	-1.21	0.227				
**Impaired attention/concentration**	1	0.34	0.732	2 (bulbar ALS-like)	11.77	<0.001	Worsens
	2	1.22	0.226				
	3	0.14	0.889				
**Bradyphrenia**	1	-0.15	0.878	2 (bulbar ALS-like)	9.93	<0.001	Worsens
	2	1.68	0.095				
	3	0.26	0.792				
**Motor**							
**Dysarthria**	1	-1.42	0.158	3 (limb ALS-like) 2 (bulbar ALS-like)	4.03	<0.001	Worsens
	2	3.71	<0.001				
	3	3.95	<0.001				
**Dysphagia**	1	-0.84	0.400	3 (limb ALS-like) 2 (bulbar ALS-like)	2.47	0.015	Worsens
	2	2.54	0.012				
	3	2.94	0.004				
**Tremor**	1	-1.09	0.278	3 (limb ALS-like)	2.85	0.005	Worsens
	2	-0.76	0.446				
	3	3.63	<0.001				
**Slowness**	1	-2.00	0.047	3 (limb ALS-like)	6.81	<0.001	Worsens
	2	1.60	0.112				
	3	6.02	<0.001				
**Weakness**	1	0.69	0.489	3 (limb ALS-like)	1.93	0.055	No significant change
	2	0.60	0.553				
	3	8.90	<0.001				
**Gait disorder**	1	-0.67	0.504	3 (limb ALS-like)	5.86	<0.001	Worsens
	2	0.08	0.935				
	3	10.48	<0.001				
**Falls**	1	0.55	0.581	3 (limb ALS-like)	3.82	<0.001	Worsens
	2	0.80	0.424				
	3	6.39	<0.001				

**Functional difficulties using hands**	**1**	0.10	0.924	3 (limb ALS-like)	5.43	<0.001	Worsens
	**2**	0.99	0.323				
	**3**	7.5	<0.001				

(b)

**Symptom**	**Subtype**	**SuStaIn subtype**		**Group with worse score**	**SuStaIn stage**		**Change with SuStaIn stage**
		***t* value**	***p* value**		***t* value**	***p* value**	
**Behavioural**							
**Disinhibition**	**1**	0.27	0.788	1 (bvFTD-parkinsonian-like)	4.03	<0.001	Worsens
	**2**	-4.37	<0.001				
**Apathy**	**1**	0.60	0.549	1 (bvFTD-parkinsonian-like)	9.43	<0.001	Worsens
	**2**	-2.25	0.027				
**Loss of sympathy/empathy**	**1**	0.30	0.767	1 (bvFTD-parkinsonian-like)	8.72	<0.001	Worsens
	**2**	-2.79	0.006				
**Ritualistic/compulsive behaviour**	**1**	0.21	0.835	1 (bvFTD-parkinsonian-like)	3.58	0.001	Worsens
	**2**	-2.98	0.004				
**Hyperorality and appetite changes**	**1**	-0.65	0.518	1 (bvFTD-parkinsonian-like)	4.9	<0.001	Worsens
	**2**	-2.4	0.019				
**Poor response to social/emotional cues**	**1**	-0.75	0.454	1 (bvFTD-parkinsonian-like)	10.13	<0.001	Worsens
	**2**	-3.44	0.001				
**Inappropriate trusting behaviour**	**1**	-0.41	0.684	1 (bvFTD-parkinsonian-like)	5.46	<0.001	Worsens
	**2**	-4.65	<0.001				
**Neuropsychiatric**							
**Hallucinations**	**1**	0.00	0.998	1 (bvFTD-parkinsonian-like)	4.22	<0.001	Worsens
	**2**	-2.43	0.017				
**Delusions**	**1**	0.48	0.631	1 (bvFTD-parkinsonian-like)	2.37	0.020	Worsens
	**2**	-1.74	0.085				
**Irritability/lability**	**1**	0.55	0.586	1 (bvFTD-parkinsonian-like)	1.76	0.082	No significant change
	**2**	-2.95	0.004				

**Agitation/aggression**	**1**	0.11	0.912	1 (bvFTD-parkinsonian-like)	2.10	0.039	Worsens
	**2**	-1.90	0.061				
**Euphoria/elation**	**1**	0.62	0.539	1 (bvFTD-parkinsonian-like)	3.04	0.003	Worsens
	**2**	-1.16	0.250				
**Aberrant motor behaviour**	**1**	0.96	0.339	1 (bvFTD-parkinsonian-like)	5.9	<0.001	Worsens
	**2**	-2.57	0.012				
**Hypersexuality**	**1**	1.41	0.164	1 (bvFTD-parkinsonian-like)	3.7	<0.001	Worsens
	**2**	-2.25	0.027				
**Hyperreligiosity**	**1**	0.26	0.799	1 (bvFTD-parkinsonian-like)	1.74	0.086	No significant change
	**2**	-1.27	0.209				
**Altered sense of humour**	**1**	-1.27	0.206	2 (PPA-like)	4.18	<0.001	Worsens
	**2**	-1.25	0.216	1 (bvFTD-parkinsonian-like)			
**Language**							
**Impaired articulation**	**1**	-0.31	0.759	2 (PPA-like)	8.15	<0.001	Worsens
	**2**	5.44	<0.001				
**Decreased fluency**	**1**	-1.18	0.243	2 (PPA-like)	8.05	<0.001	Worsens
	**2**	7.44	<0.001				
**Impaired grammar/syntax**	**1**	-2.36	0.021	2 (PPA-like)	6.72	<0.001	Worsens
	**2**	7.78	<0.001				
**Impaired word retrieval**	**1**	-0.52	0.606	2 (PPA-like)	4.62	<0.001	Worsens
	**2**	7.18	<0.001				
**Impaired speech repetition**	**1**	-1.01	0.314	2 (PPA-like)	7.23	<0.001	Worsens
	**2**	4.93	<0.001				
**Impaired sentence comprehension**	**1**	0.19	0.846	2 (PPA-like)	7.58	<0.001	Worsens
	**2**	3.93	<0.001				
**Impaired single word comprehension**	**1**	0.13	0.899	2 (PPA-like)	4.68	<0.001	Worsens
	**2**	3.7	<0.001				
**Dyslexia**	**1**	-0.78	0.436	2 (PPA-like)	8.44	<0.001	Worsens
	**2**	4.29	<0.001				

**Dysgraphia**	**1**	-1.25	0.214	2 (PPA-like)	8.85	<0.001	Worsens
	**2**	4.77	<0.001				
**Impaired functional communication**	**1**	-0.39	0.699	2 (PPA-like)	11.71	<0.001	Worsens
	**2**	7.34	<0.001				
**Cognitive**							
**Memory impairment**	**1**	0.91	0.364	1 (bvFTD-parkinsonian-like)	6.63	<0.001	Worsens
	**2**	-2.64	0.010				
**Impaired orientation**	**1**	1.14	0.256	1 (bvFTD-parkinsonian-like)	9.78	<0.001	Worsens
	**2**	-3.21	0.002				
**Impaired judgement and problem solving**	**1**	0.27	0.785	1 (bvFTD-parkinsonian-like)	14.59	<0.001	Worsens
	**2**	-2.20	0.031				
**Problems with community affairs**	**1**	1.24	0.218	1 (bvFTD-parkinsonian-like)	13.39	<0.001	Worsens
	**2**	-0.93	0.354				
**Problems at homes or with hobbies**	**1**	2.51	0.014	1 (bvFTD-parkinsonian-like)	16.66	<0.001	Worsens
	**2**	-2.35	0.021				
**Impaired personal care**	**1**	1.38	0.173	1 (bvFTD-parkinsonian-like)	12.89	<0.001	Worsens
	**2**	-3.92	<0.001				
**Person recognition difficulty**	**1**	-0.08	0.936	1 (bvFTD-parkinsonian-like)	5.47	<0.001	Worsens
	**2**	-1.54	0.129				
**Impaired topographical memory**	**1**	0.21	0.832	1 (bvFTD-parkinsonian-like)	7.86	<0.001	Worsens
	**2**	-2.59	0.012				
**Visuospatial or perceptual impairment**	**1**	0.62	0.539	1 (bvFTD-parkinsonian-like)	6.02	<0.001	Worsens
	**2**	-0.83	0.411				
**Impaired attention/concentration**	**1**	1.66	0.100	1 (bvFTD-parkinsonian-like)	10.40	<0.001	Worsens
	**2**	-0.61	0.545				
**Bradyphrenia**	**1**	2.31	0.023	1 (bvFTD-parkinsonian-like)	9.64	<0.001	Worsens
	**2**	-1.94	0.056				
**Motor**							
**Dysarthria**	**1**	0.62	0.540	2 (PPA-like)	7.1	<0.001	Worsens

	**2**	3.42	0.001				
**Dysphagia**	**1**	-0.06	0.949	1 (bvFTD-parkinsonian-like)	5.53	<0.001	Worsens
	**2**	-0.93	0.353				
**Tremor**	**1**	0.24	0.807	1 (bvFTD-parkinsonian-like)	3.73	<0.001	Worsens
	**2**	-1.05	0.296				
**Slowness**	**1**	-0.54	0.589	1 (bvFTD-parkinsonian-like)	8.23	<0.001	Worsens
	**2**	-1.38	0.172				
**Weakness**	**1**	0.24	0.812	1 (bvFTD-parkinsonian-like)	4.83	<0.001	Worsens
	**2**	-1.39	0.168				
**Gait disorder**	**1**	-1.23	0.221	2 (PPA-like)	5.02	<0.001	Worsens
	**2**	-0.66	0.513				
**Falls**	**1**	-0.14	0.886	2 (PPA-like)1 (bvFTD-parkinsonian-like)	3.49	<0.001	Worsens
	**2**	0.22	0.823				
**Functional difficulties using hands**	**1**	-0.05	0.962	1 (bvFTD-parkinsonian-like) 2 (PPA-like)	7.00	<0.001	Worsens
	**2**	-0.11	0.911				

Supplementary Figure 1 Cross-validation for three subtypes in (A, B) *C9orf72*, (C, D) *GRN*, and (E, F) *MAPT* mutation carriers. (A, C, and E) Each data-point represents the Cross-Validation Information Criterion for a particular number of subtypes, which are indexed from 0 in python, i.e. ‘0 Subtypes model’ corresponds to a model with one subtype, ‘1 Subtypes model’ corresponds to a model with two subtypes, and ‘2 Subtypes model’ corresponds to a model with three subtypes. (B, D, and F) Each bar represents the test set log-likelihood across the ten different folds for a particular number of subtypes which are indexed from 0 in python, i.e. ‘s_0 Subtypes model’ corresponds to a model with one subtype, ‘s_1 Subtypes model’ corresponds to a model with two subtypes, and ‘s_2 Subtypes model’ corresponds to a model with three subtypes. For *C9orf72* (N=280), three distinct subtypes are shown, the maximum likelihood distributions of which do not overlap on the graph. Although there is a plateau forming on the Cross-Validation Information Criterion (CVIC) plot, there is a downward trend that indicates cross-validation should be run on four subtypes to ensure there is no additional *C9orf72* sub-phenotype to account for. For *GRN* (N=242), two distinct subtypes are shown, after which the CVIC graph plateaus. For *MAPT* (N=101), similar box plots are shown across the folds despite a down-sloping CVIC graph, supporting only a single symptom progression profile.

A

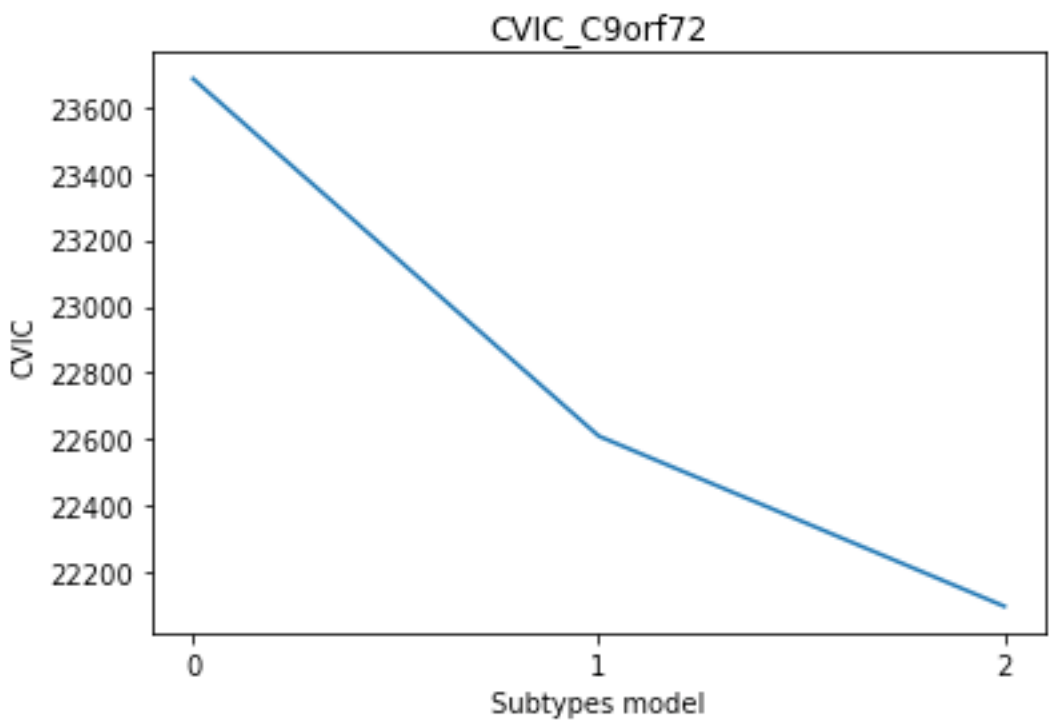

B

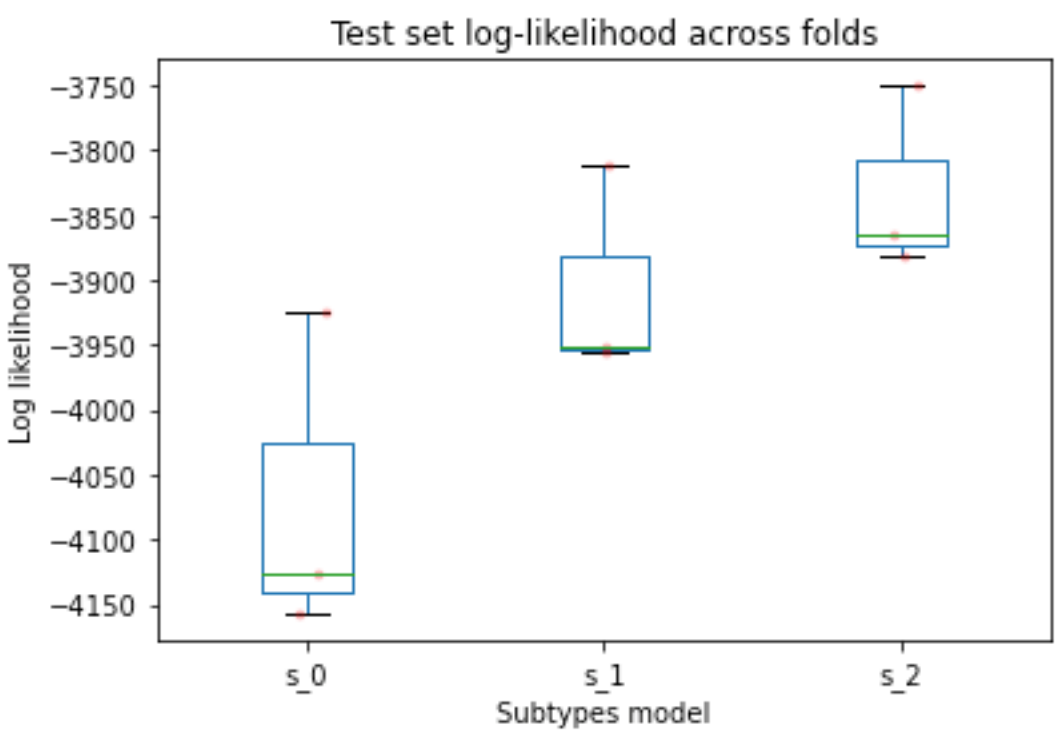

C

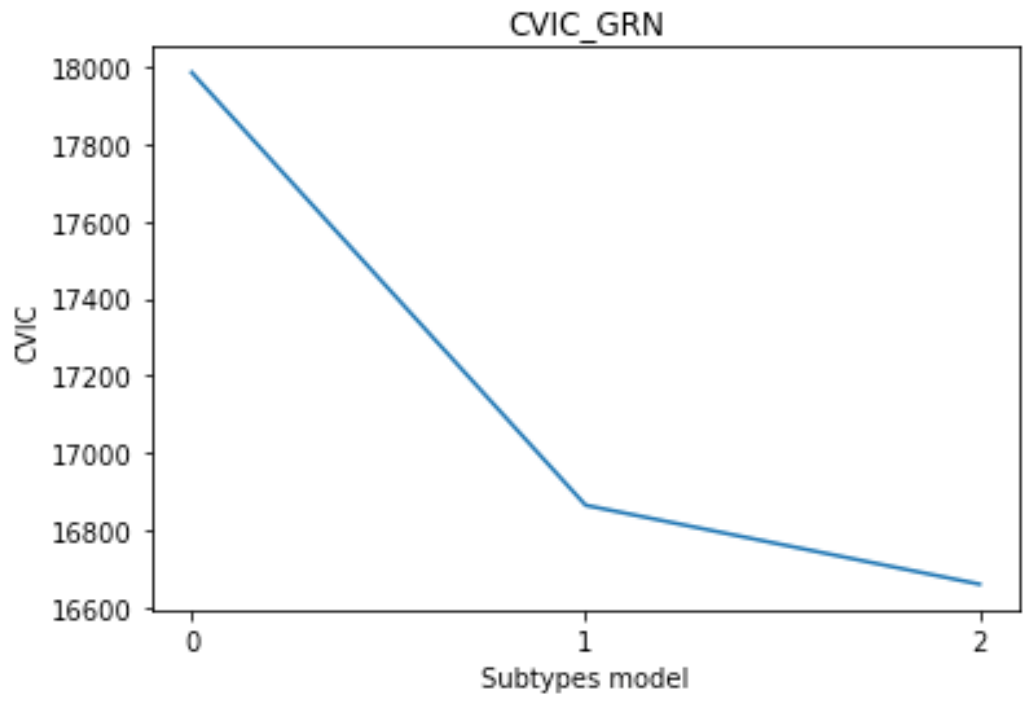
CVIC_GRN
CVIC
18000
17800
17600
17400
17200
17000
16800
16600
0
1
2
Subtypes model

D

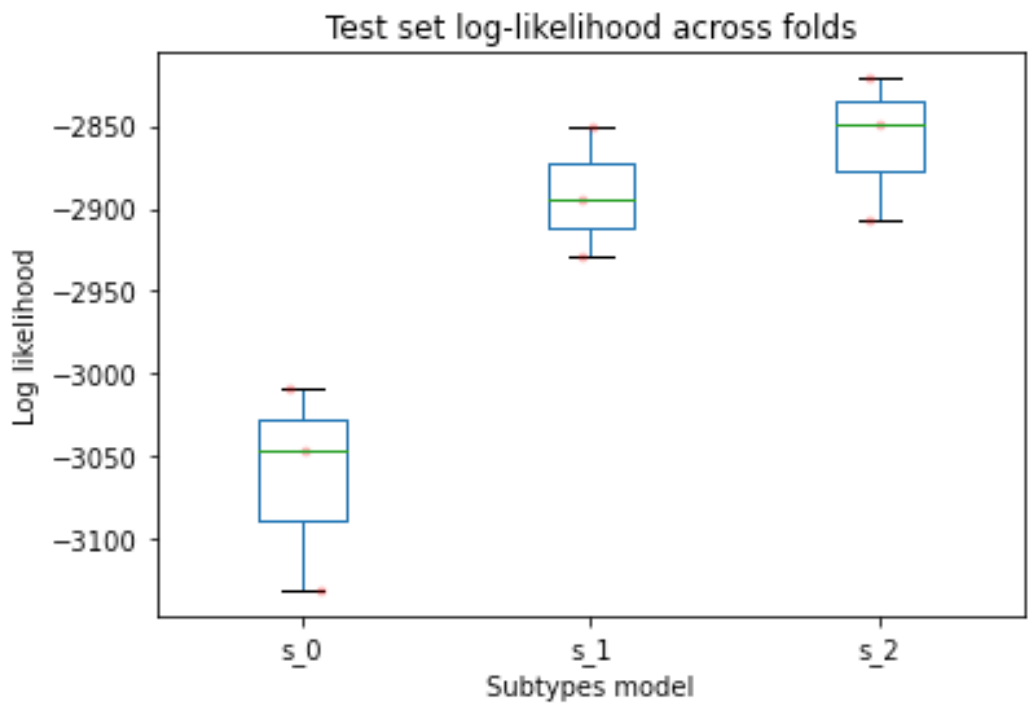
Test set log-likelihood across folds
Log likelihood
−2850
−2900
−2950
−3000
−3050
−3100
s_0
s_1
s_2
Subtypes model

E

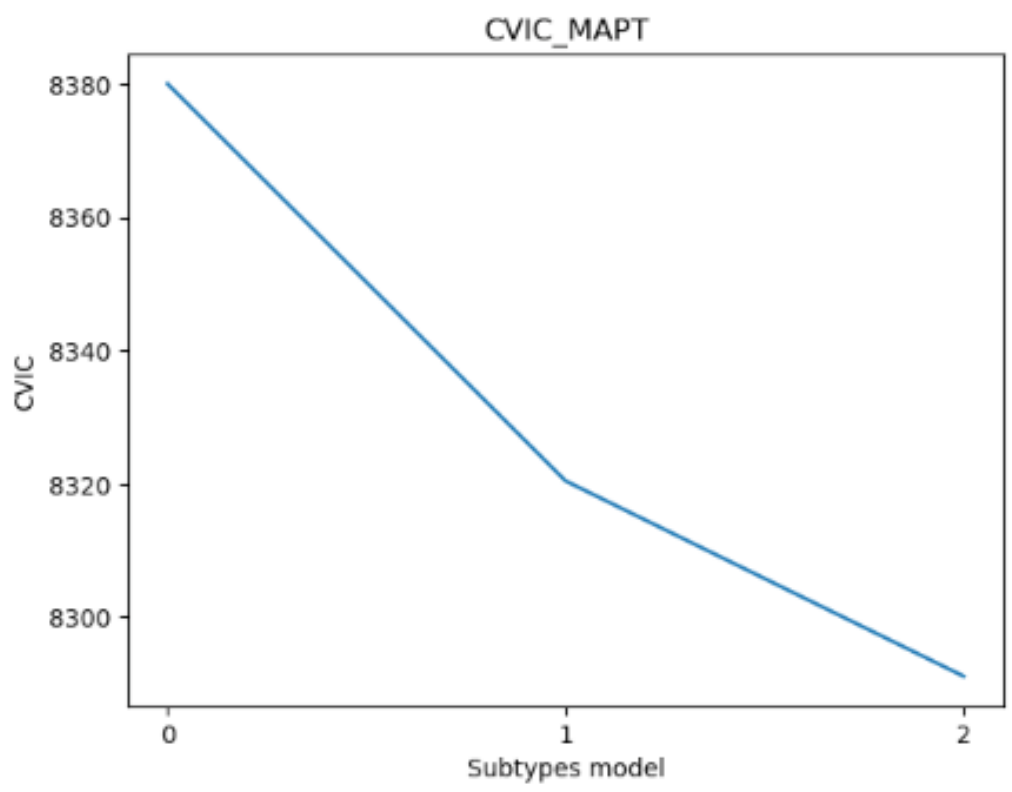
CVIC_MAPT
CVIC
8380
8360
8340
8320
8300
0
1
2
Subtypes model

F

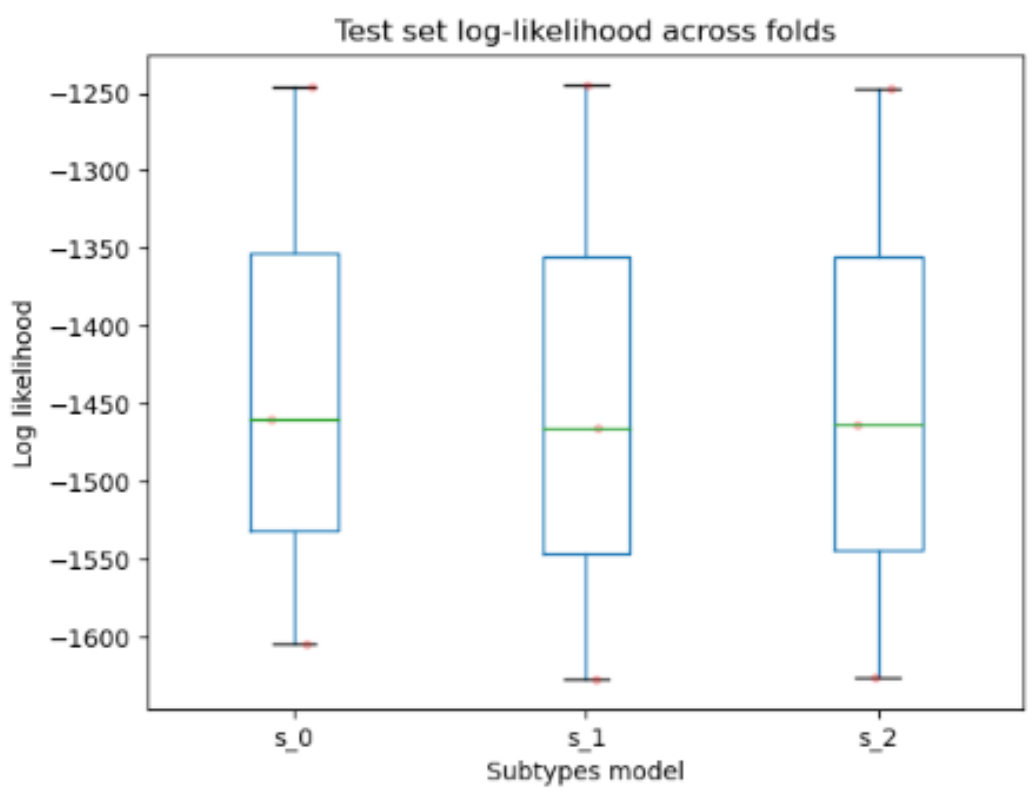
Test set log-likelihood across folds
Log likelihood
−1250
−1300
−1350
−1400
−1450
−1500
−1550
−1600
s_0
s_1
s_2
Subtypes model

Supplementary Figure 2 Cross-validation for four subtypes in *C9orf72* mutation carriers (N=280). (A) Each data-point represents the Cross-Validation Information Criterion (CVIC) for a particular number of subtypes, which are indexed from 0 in python, i.e. '0 Subtypes model' corresponds to a model with one subtype, '1 Subtypes model' corresponds to a model with two subtypes, '2 Subtypes model' corresponds to a model with three subtypes, and '3 Subtypes model' corresponds to a model with four subtypes. (B) Each bar represents the test set log-likelihood across the ten different folds for a particular number of subtypes which are indexed from 0 in python, i.e. 's_0 Subtypes model' corresponds to a model with one subtype, 's_1 Subtypes model' corresponds to a model with two subtypes, 's_2 Subtypes model' corresponds to a model with three subtypes, and 's_3 Subtypes model' corresponds to a model with four subtypes. This CVIC plot verified that a three-subtype model best describes the heterogeneity in symptom progression patterns for *C9orf72* mutation carriers. This is shown by the upward trend after three subtype models have been plotted.

A

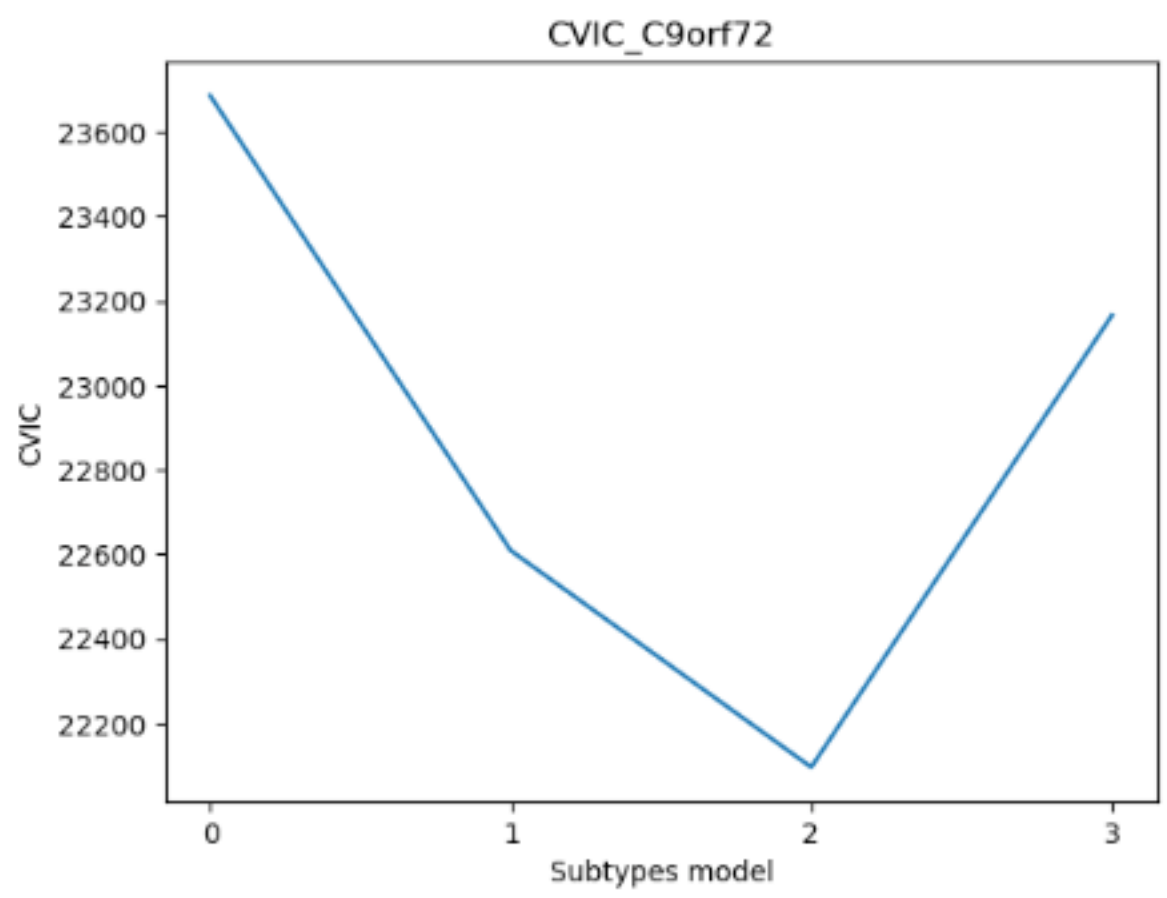

B

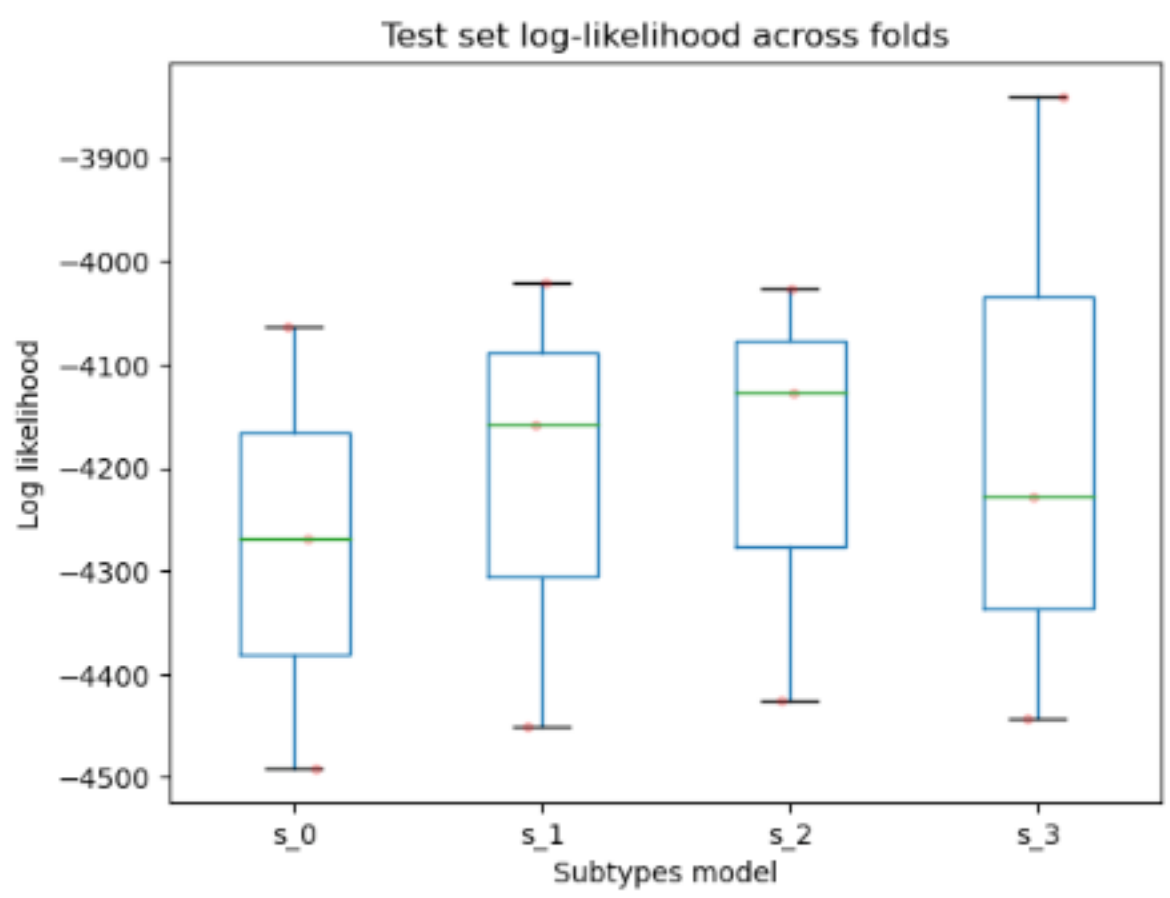

Supplementary Figure 3 *C9orf72* subtype assignment which shows the probability of belonging to the maximum likelihood (ml) subtype for individuals at each stage. For each subtype the plots are above the dotted line and increase with increasing SuStaIn stage, indicating the subtypes are well-differentiated. Data points for subtype 1: stage 1, n=16; 2, n=6; 3, n=7; 4, n=5; 5, n=5, 6, n=5; 7, n=6; 8, n=5; 9, n=5; 10, n=6; 11, n=5; 12, n=5; 13, n=5; 14, n=5; 15, n=2, subtype 2: stage 1, n=5; 2, n=6; 3, n=5; 4, n=5; 5, n=6; 6, n=6; 7, n=2, and subtype 3: stage 1, n=5; 2, n=5; 3, n=5; 4, n=5; 5, n=2.

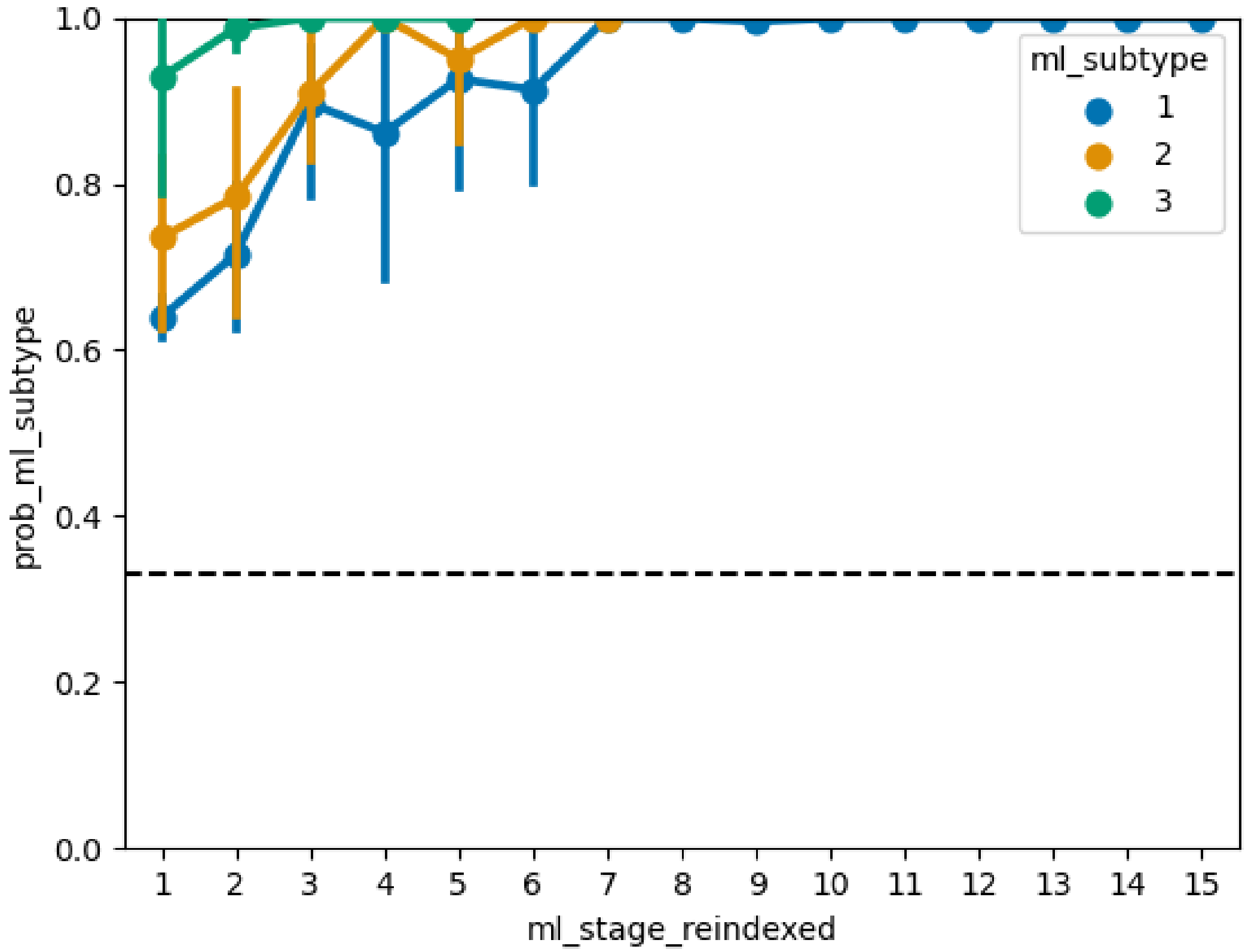

Supplementary Figure 4 *GRN* subtype assignment which shows the probability of belonging to the maximum likelihood (ml) subtype for individuals at each stage. For each subtype the plots are above the dotted line and increase with increasing SuStaIn stage, indicating the subtypes are well-differentiated. Data points for subtype 1: stage 1, n=7; 2, n=7; 3, n=5; 4, n=5; 5, n=6; 6, n=5; 7, n=5; 8, n=5; 9, n=5; 10, n=2, and subtype 2: stage 1, n=5; 2, n=6; 3, n=6; 4, n=6; 5, n=5; 6, n=5; 7, n=4.

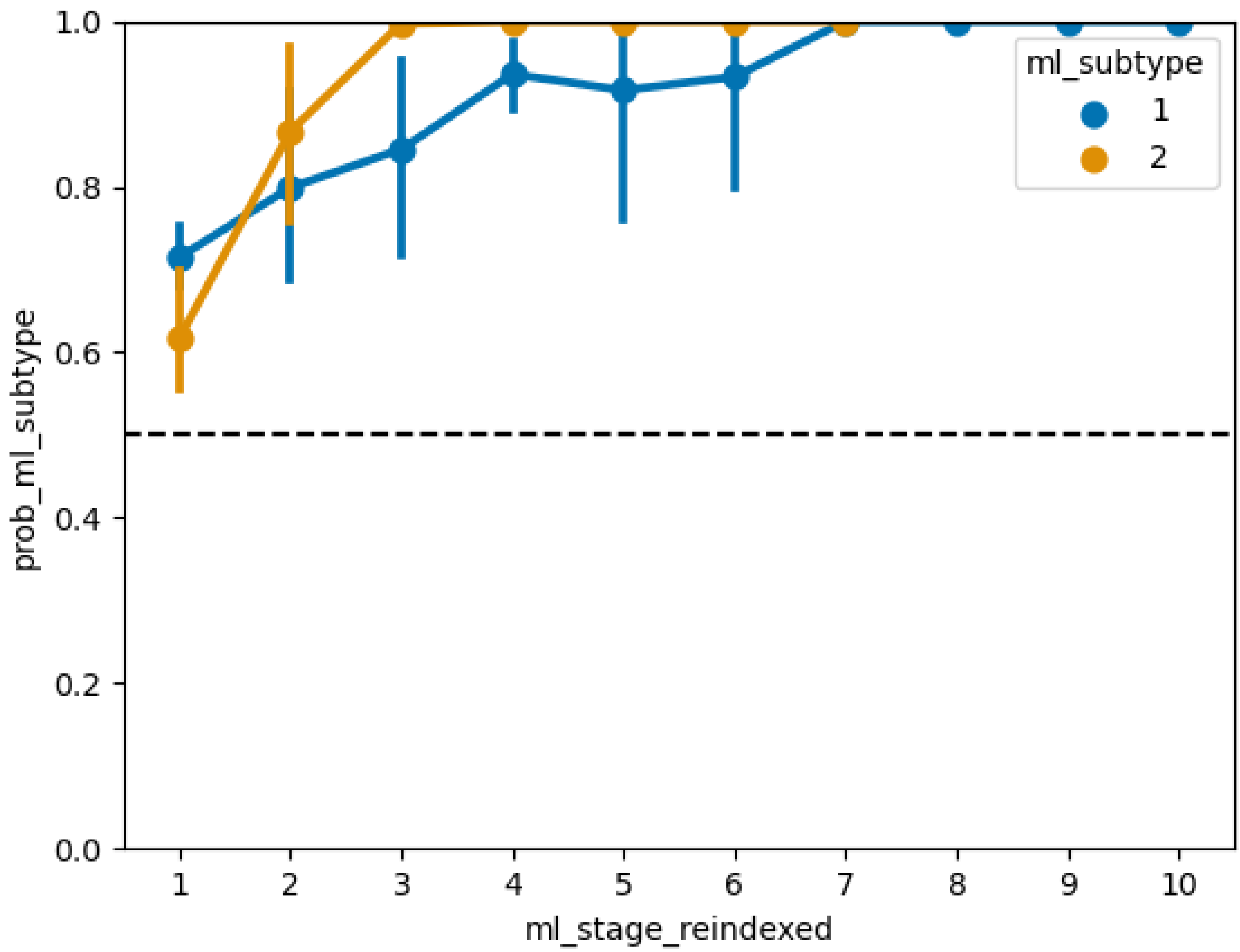

Supplementary Figure 5 Ordinal SuStaIn Plots for multiple subtypes in each mutation group as identified by cross-validation including (A) *C9orf72* subtype 1, bvFTD-like (N=88), (B) 2, bulbar ALS-like (N=35), (C) 3, limb ALS-like (N=22), (D) *GRN* subtype 1, bvFTD-parkinsonism-like (N=52), (E) 2, PPA-like (N=37), and (F) *MAPT*, semantic-behavioural-parkinsonism-like (N=49). Full outputs are shown, and each coloured square represents a change in a symptom severity score in the individuals within the group, as illustrated by the heatmap scale bar.

A

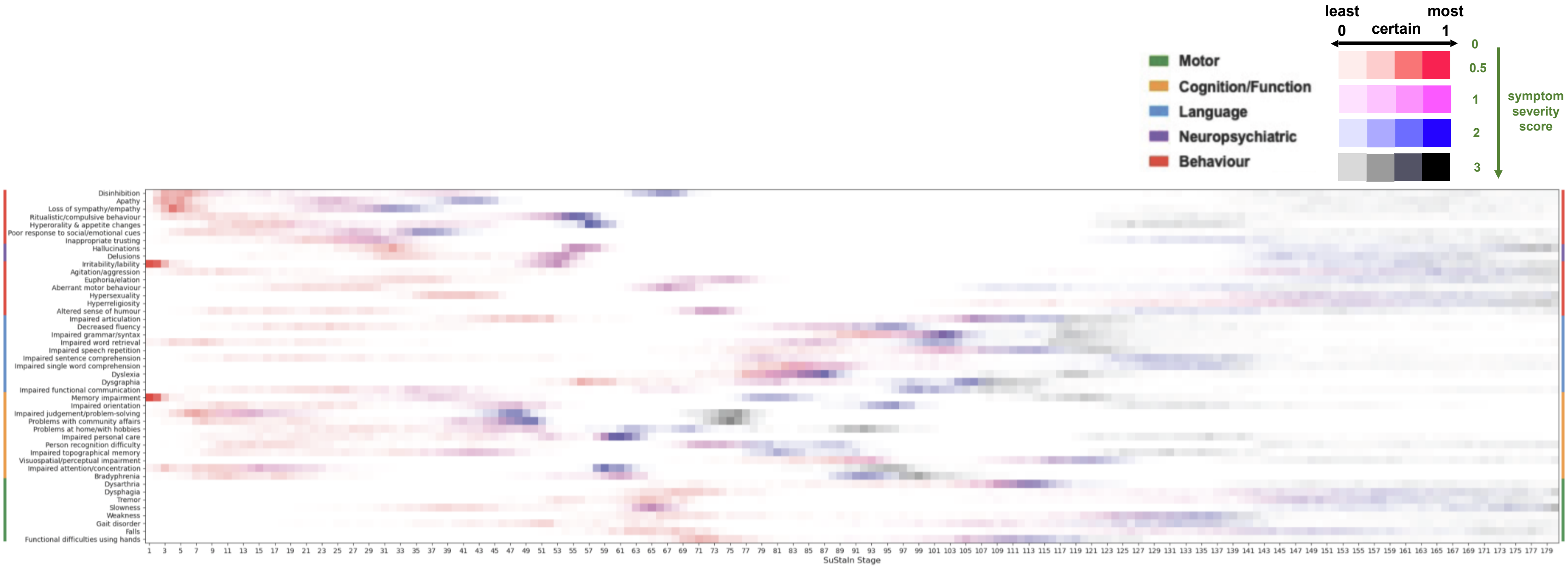

B

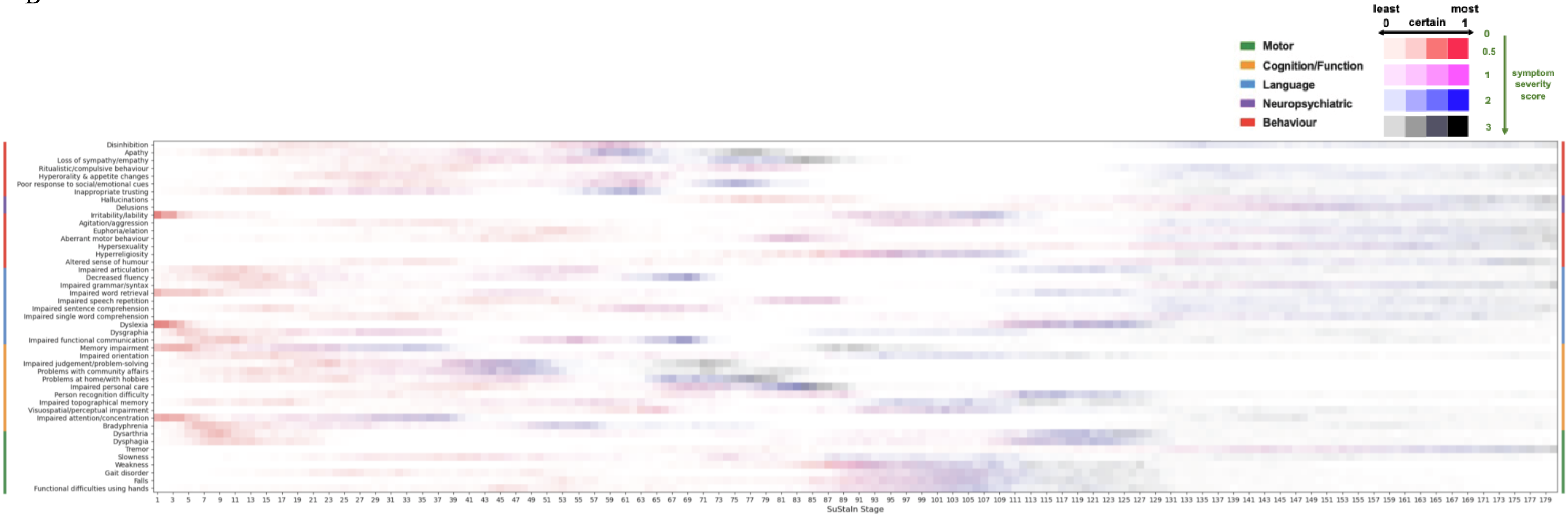

least
most
0
certain
1
Motor
Cognition/Function
Language
Neuropsychiatric
Behaviour
0
0.5
1
2
3
symptom
severity
score
Disinhibition
Apathy
Loss of sympathy/empathy
Ritualistic/compulsive behaviour
Hyperorality & appetite changes
Poor response to social/emotional cues
Inappropriate trusting
Hallucinations
Delusions
Irritability/lability
Agitation/aggression
Euphoria/elation
Aberrant motor behaviour
Hypersexuality
Hyperreligiosity
Altered sense of humour
Impaired articulation
Decreased fluency
Impaired grammar/syntax
Impaired word retrieval
Impaired speech repetition
Impaired sentence comprehension
Impaired single word comprehension
Dyslexia
Dysgraphia
Impaired functional communication
Memory impairment
Impaired orientation
Impaired judgement/problem-solving
Problems with community affairs
Problems at home/with hobbies
Impaired personal care
Person recognition difficulty
Impaired topographical memory
Visuospatial/perceptual impairment
Impaired attention/concentration
Bradyphrenia
Dysarthria
Dysphagia
Tremor
Slowness
Weakness
Gait disorder
Falls
Functional difficulties using hands
SuStaIn Stage

C

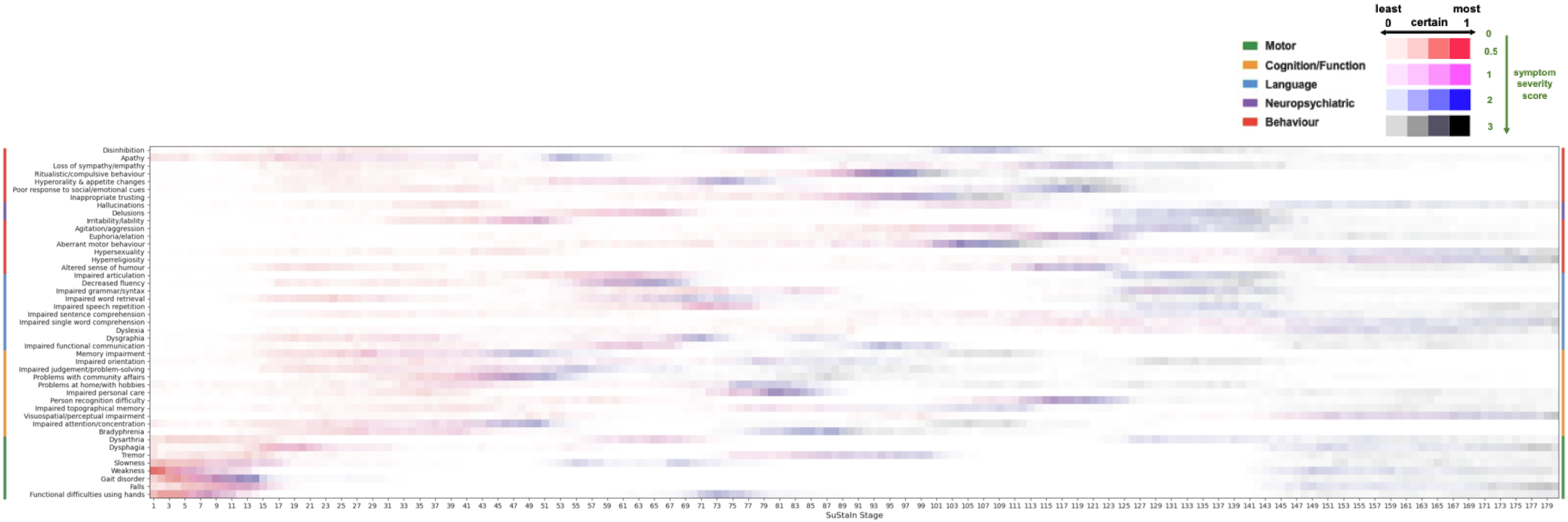

least
most
0
certain
1
Motor
Cognition/Function
Language
Neuropsychiatric
Behaviour
0
0.5
1
2
3
symptom severity score
Disinhibition
Apathy
Loss of sympathy/empathy
Ritualistic/compulsive behaviour
Hyperorality & appetite changes
Poor response to social/emotional cues
Inappropriate trusting
Hallucinations
Delusions
Irritability/lability
Agitation/aggression
Euphoria/elation
Aberrant motor behaviour
Hypersexuality
Hyperreligiosity
Altered sense of humour
Impaired articulation
Decreased fluency
Impaired grammar/syntax
Impaired word retrieval
Impaired speech repetition
Impaired sentence comprehension
Impaired single word comprehension
Dyslexia
Dysgraphia
Impaired functional communication
Memory impairment
Impaired orientation
Impaired judgement/problem-solving
Problems with community affairs
Problems at home/with hobbies
Impaired personal care
Person recognition difficulty
Impaired topographical memory
Visuospatial/perceptual impairment
Impaired attention/concentration
Bradyphrenia
Dysarthria
Dysphagia
Tremor
Slowness
Weakness
Gait disorder
Falls
Functional difficulties using hands
1 3 5 7 9 11 13 15 17 19 21 23 25 27 29 31 33 35 37 39 41 43 45 47 49 51 53 55 57 59 61 63 65 67 69 71 73 75 77 79 81 83 85 87 89 91 93 95 97 99 101 103 105 107 109 111 113 115 117 119 121 123 125 127 129 131 133 135 137 139 141 143 145 147 149 151 153 155 157 159 161 163 165 167 169 171 173 175 177 179
SuStaIn Stage

D

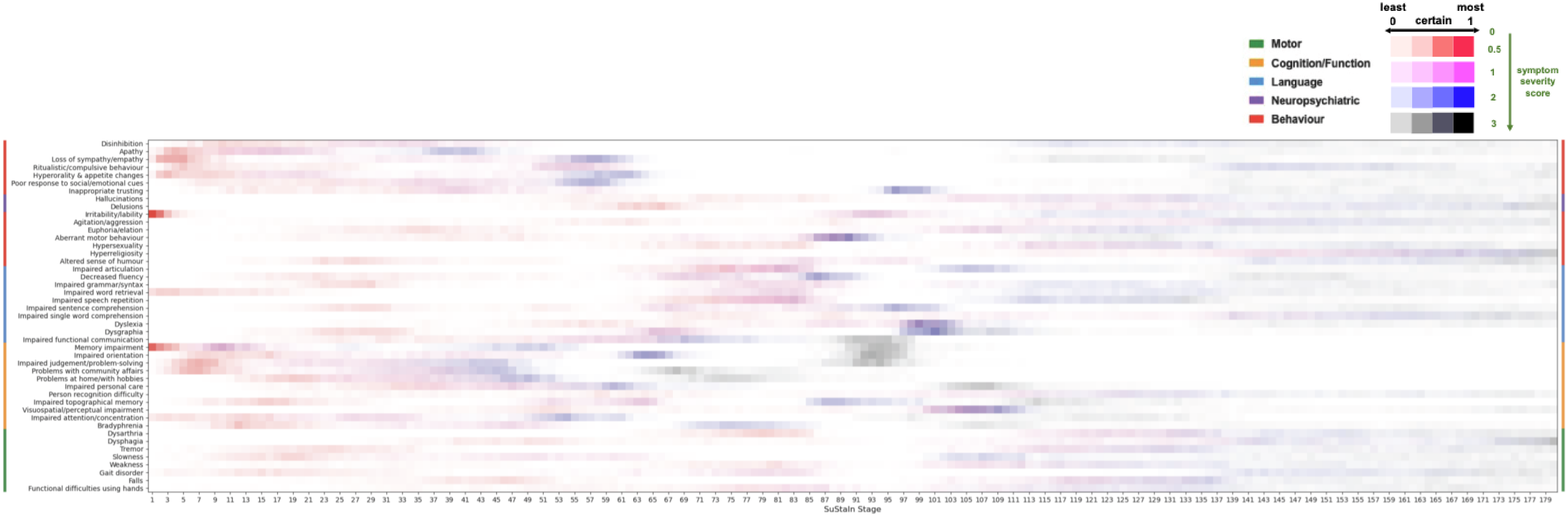
least
most
0
certain
1
Motor
Cognition/Function
Language
Neuropsychiatric
Behaviour
0
0.5
1
2
3
symptom severity score
Disinhibition
Apathy
Loss of sympathy/empathy
Ritualistic/compulsive behaviour
Hyperorality & appetite changes
Poor response to social/emotional cues
Inappropriate trusting
Hallucinations
Delusions
Irritability/lability
Agitation/aggression
Euphoria/elation
Aberrant motor behaviour
Hypersexuality
Hyperreligiosity
Altered sense of humour
Impaired articulation
Decreased fluency
Impaired grammar/syntax
Impaired word retrieval
Impaired speech repetition
Impaired sentence comprehension
Impaired single word comprehension
Dyslexia
Dysgraphia
Impaired functional communication
Memory impairment
Impaired orientation
Impaired judgement/problem-solving
Problems with community affairs
Problems at home/with hobbies
Impaired personal care
Person recognition difficulty
Impaired topographical memory
Visuospatial/perceptual impairment
Impaired attention/concentration
Bradyphrenia
Dysarthria
Dysphagia
Tremor
Slowness
Weakness
Gait disorder
Falls
Functional difficulties using hands
SuStaIn Stage

E

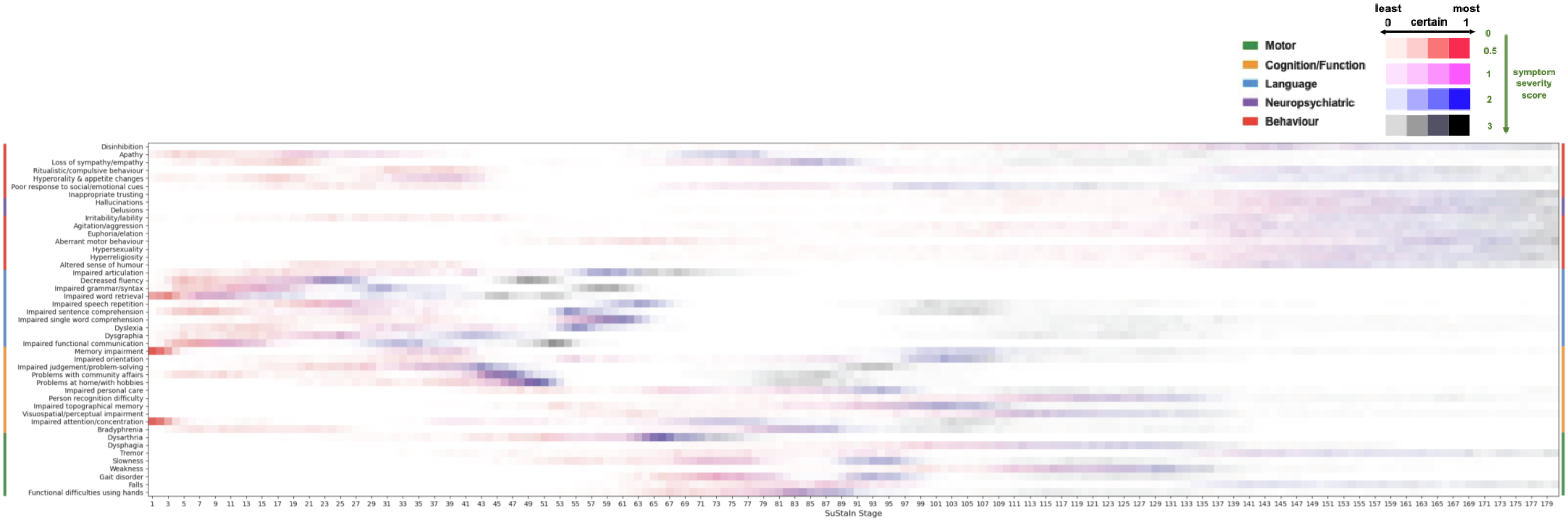
least
most
0
certain
1
Motor
Cognition/Function
Language
Neuropsychiatric
Behaviour
0
0.5
1
2
3
symptom severity score
Disinhibition
Apathy
Loss of sympathy/empathy
Ritualistic/compulsive behaviour
Hyperorality & appetite changes
Poor response to social/emotional cues
Inappropriate trusting
Hallucinations
Delusions
Irritability/lability
Agitation/aggression
Euphoria/elation
Aberrant motor behaviour
Hypersexuality
Hyperreligiosity
Altered sense of humour
Impaired articulation
Decreased fluency
Impaired grammar/syntax
Impaired word retrieval
Impaired speech repetition
Impaired sentence comprehension
Impaired single word comprehension
Dyslexia
Dysgraphia
Impaired functional communication
Memory impairment
Impaired orientation
Impaired judgement/problem-solving
Problems with community affairs
Problems at home/with hobbies
Impaired personal care
Person recognition difficulty
Impaired topographical memory
Visuospatial/perceptual impairment
Impaired attention/concentration
Bradyphrenia
Dysarthria
Dysphagia
Tremor
Slowness
Weakness
Gait disorder
Falls
Functional difficulties using hands
SuStaIn Stage

F

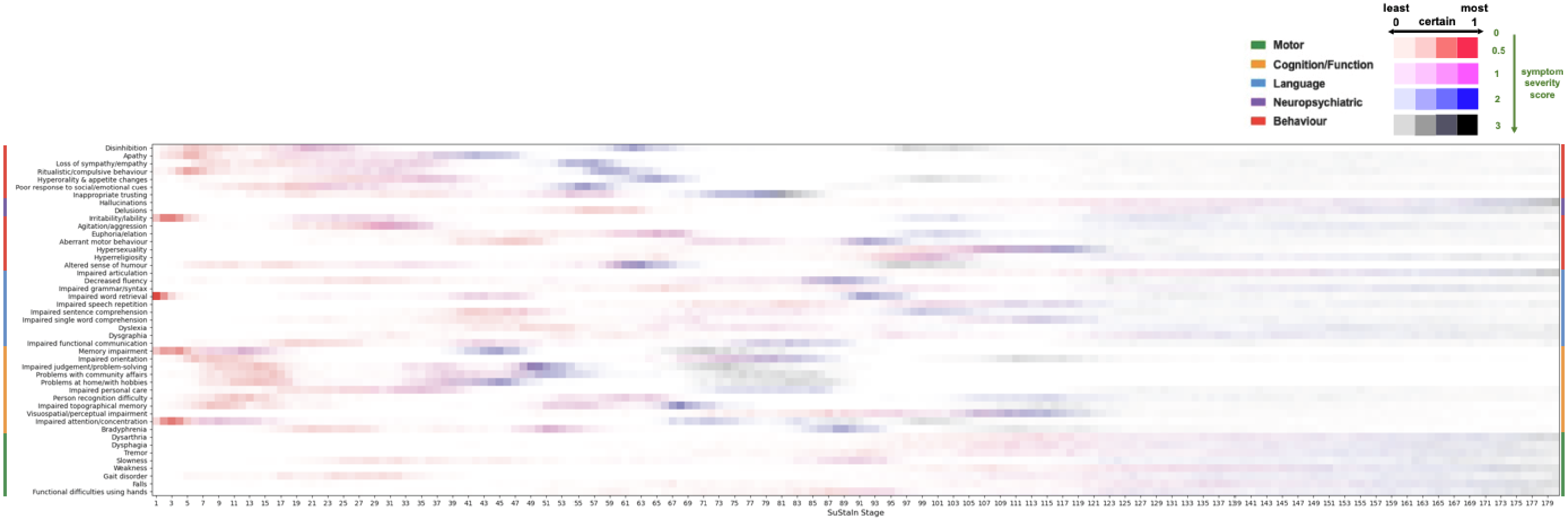
least
most
0
certain
1
Motor
Cognition/Function
Language
Neuropsychiatric
Behaviour
0
0.5
1
2
3
symptom
severity
score
Disinhibition
Apathy
Loss of sympathy/empathy
Ritualistic/compulsive behaviour
Hyperorality & appetite changes
Poor response to social/emotional cues
Inappropriate trusting
Hallucinations
Delusions
Irritability/lability
Agitation/aggression
Euphoria/elation
Aberrant motor behaviour
Hypersexuality
Hyperreligiosity
Altered sense of humour
Impaired articulation
Decreased fluency
Impaired grammar/syntax
Impaired word retrieval
Impaired speech repetition
Impaired sentence comprehension
Impaired single word comprehension
Dyslexia
Dysgraphia
Impaired functional communication
Memory impairment
Impaired orientation
Impaired judgement/problem-solving
Problems with community affairs
Problems at home/with hobbies
Impaired personal care
Person recognition difficulty
Impaired topographical memory
Visuospatial/perceptual impairment
Impaired attention/concentration
Bradyphrenia
Dysarthria
Dysphagia
Tremor
Slowness
Weakness
Gait disorder
Falls
Functional difficulties using hands
1 3 5 7 9 11 13 15 17 19 21 23 25 27 29 31 33 35 37 39 41 43 45 47 49 51 53 55 57 59 61 63 65 67 69 71 73 75 77 79 81 83 85 87 89 91 93 95 97 99 101 103 105 107 109 111 113 115 117 119 121 123 125 127 129 131 133 135 137 139 141 143 145 147 149 151 153 155 157 159 161 163 165 167 169 171 173 175 177 179
SuStaIn Stage

Supplementary Figure 6 Scatter plots of the correlation between SuStaIn Stage and expected years to onset (EYO) in each of the genetic subtypes. Using Spearman's rank correlation, each data-point represents an individual's Expected Years to Onset in relation to their SuStaIn stage. *C9orf72* Subtype 1 = bvFTD-like (N=88); *C9orf72* Subtype 2 = bulbar ALS-like (N=35); *C9orf72* Subtype 3 = limb ALS-like (N=22); *GRN* Subtype 1 = bvFTD-parkinsonian-like (N=52); *GRN* Subtype 2 = PPA-like (N=37); *MAPT* = semantic-behavioural-parkinsonian-like (N=49). SuStaIn Stage significantly positively correlated with EYO (all $p<0.001$ apart from *C9orf72* subtype 3 with $p=0.068$ and *GRN* subtype 1 with $p=0.004$). Correlation coefficients: *C9orf72* subtype 1 (*rho*=0.517), subtype 2 (*rho*=0.393), subtype 3 (*rho*=0.397); *GRN* subtype 1 (*rho*=0.395), subtype 2 (*rho*=0.522); *MAPT* (*rho*=0.728).

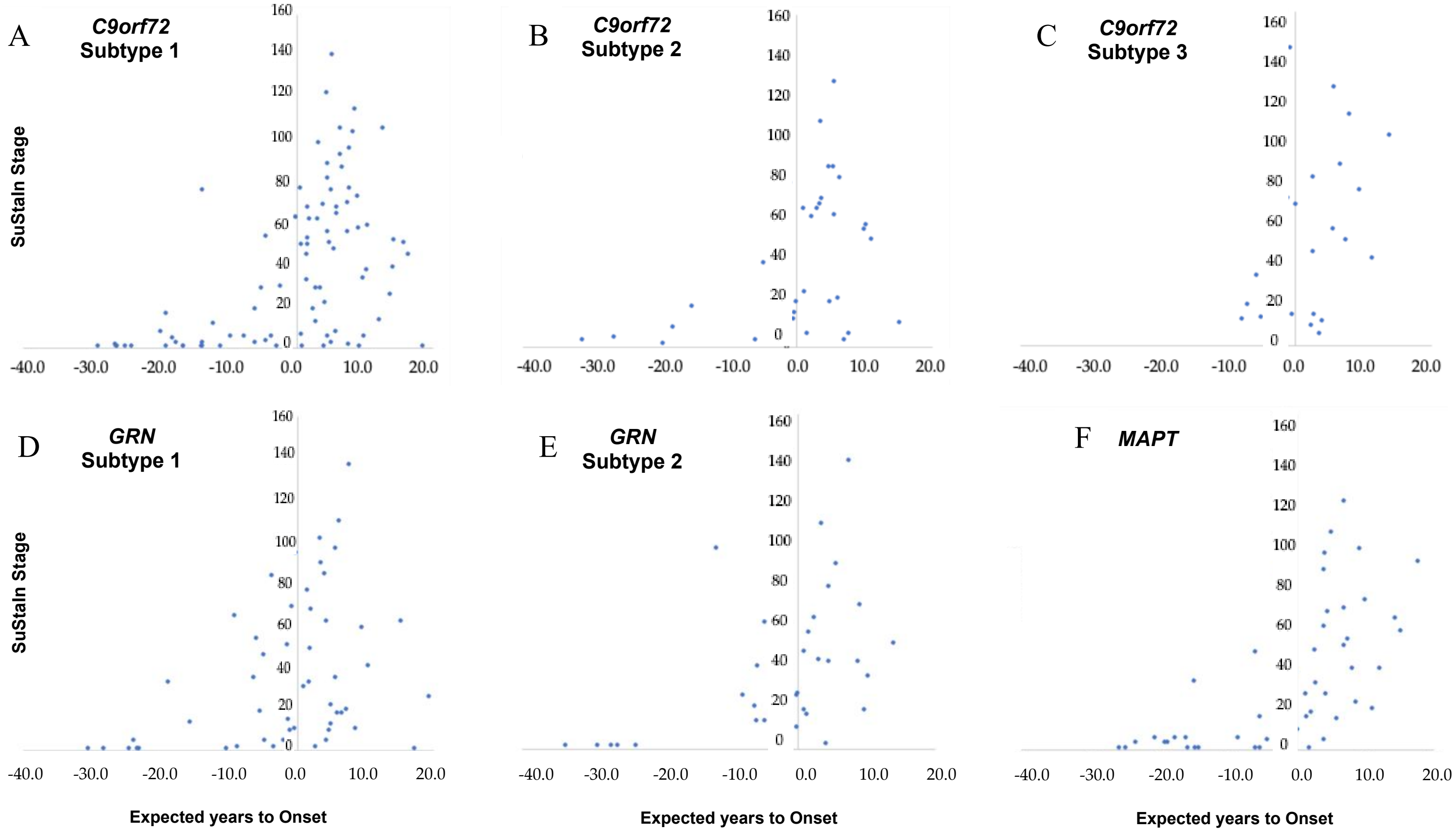
A
C9orf72
Subtype 1
B
C9orf72
Subtype 2
C
C9orf72
Subtype 3
D
GRN
Subtype 1
E
GRN
Subtype 2
F
MAPT
SuStaIn Stage
Expected years to Onset
0
20
40
60
80
100
120
140
160
-40.0
-30.0
-20.0
-10.0
0.0
10.0
20.0

Supplementary Appendix

Supplementary Table 7 shows the list of 45 symptoms within the GENFI symptom scales.

Domain	Symptom
**Behaviour**	Disinhibition
	Apathy
	Loss of sympathy/empathy
	Ritualistic/compulsive behaviour
	Hyperorality and appetite changes
	Poor response to social/emotional cues
	Inappropriate trusting behaviour
	Irritability/lability
	Agitation/aggression
	Euphoria/elation
	Aberrant motor behaviour
	Hypersexuality
	Hyperreligiosity
	Altered sense of humour
**Neuropsychiatric**	Hallucinations
	Delusions
**Language**	Impaired articulation
	Decreased fluency
	Impaired grammar/syntax
	Impaired word retrieval
	Impaired speech repetition
	Impaired sentence comprehension
	Impaired single word comprehension
	Dyslexia (impaired reading)
	Dysgraphia (impaired writing)
	Impaired functional communication
**Cognition and Function**	Memory impairment
	Impaired orientation
	Impaired judgement/problem-solving
	Problems with community affairs
	Problems at home or with hobbies
	Impaired personal care
	Person recognition difficulty
	Impaired topographical memory
	Visuospatial/perceptual impairment
	Impaired attention/concentration
	Bradyphrenia (slowness of thought)
**Motor**	Dysarthria
	Dysphagia
	Tremor
	Slowness
	Weakness
	Gait disorder
	Falls
	Functional difficulties using hands

Single progression patterns

Demographic and clinical score data for the single genetic symptom progression profiles and subtype 0, which represents those with few/no symptoms are shown below. The subtype 0 group were significantly younger with more EYO than the single symptom progression groups. Subtype 0 also had more educational attainment, with significance compared with the *GRN* symptom progression profile (p=0.005). Clinical severity scores were significantly less severe in subtype 0 compared with the single symptom progression profile mutation carriers, apart from ALSFRS-R in the *MAPT* group (p=0.894).

Supplementary Table 8 **shows demographic and clinical score data for the single genetic symptom progression profiles and subtype 0.** Data are shown as mean (standard deviation). Bold items are significantly different to subtype 0; [a]significantly younger/less impaired/more EYO compared to *GRN* mutation carriers; [b]significantly younger/less impaired/more EYO compared to *C9orf72* mutation carriers. MMSE was performed on 342 participants in the subtype 0 group, 131 *C9orf72*, 84 *GRN* and 48 *MAPT* mutation carriers. FRS was performed on 268 subtype 0, 114 *C9orf72*, 67 *GRN* and 35 *MAPT* mutation carriers.

**Subtype:**	**0**	***C9orf72***	***GRN***	***MAPT***
**Number of participants**	350	134	90	49
**% Male**	38.9	61.2	44.4	51.0
**Age in years**	44.5 (12.0)	**59.2 (11.6)**	**59.4 (10.6)**	**50.2 (13.2)[ab]**
**Education in years**	14.6 (3.4)	13.1 (3.4)	**12.5 (3.6)**	13.9 (3.2)
**Expected years to onset**	-14.0 (12.2)	**0.58 (9.9)**	-1.51 (10.2)[b]	**-1.76 (11.4)[ab]**
**Mini-Mental State Examination**	29.3 (1.2)	**24.6 (6.1)[a]**	**21.8 (8.5)**	**25.5 (6.3)**
**CDR® plus NACC FTLD Global Score**	0.1 (0.2)	**1.6 (1.0)**	**1.5 (1.0)**	**1.4 (1.0)**
**CDR® plus NACC FTLD Sum of Boxes**	0.1 (0.2)	**8.3 (6.6)**	**7.2 (6.5)**	**6.9 (6.3)[a]**
**CDR® plus NACC FTLD-NM Global Score**	0.1 (0.2)	**1.7 (1.0)**	**1.5 (1.0)**	**1.4 (1.0)**
**CDR® plus NACC FTLD-NM Sum of Boxes**	0.1 (0.3)	**9.3 (7.2)**	**7.8 (7.0)[b]**	**7.2 (6.5)[a]**
**FTD Rating Scale**	96.0 (8.2)	**47.1 (31.8)**	**55.6 (31.6)[b]**	**58.0 (31.1)**
**Progressive Aphasia Severity Scale Sum of Boxes**	0.7 (0.4)	**3.0 (4.8)**	**5.5 (7.5)**	**2.6 (3.7)**
**ALS Functional Rating Scale- Revised**	47.5 (1.9)	**34.7 (15.7)**	**33.4 (17.9)**	46.9 (3.0)[ab]
**Cambridge Behavioural Inventory-Revised**	5.1 (8.1)	**55.9 (34.4)**	**46.6 (35.0)[b]**	**44.6 (36.5)**

C9orf72

The PVD inferred by Ordinal SuStaIn for a single *C9orf72* symptom progression profile showed early behaviour (irritability/lability, disinhibition, apathy), cognition/function impairment (impaired memory, judgement and problem-solving, and attention/concentration), followed by motor symptoms including slowness, weakness, gait disorder, falls and functional difficulties using hands, with dysarthria and dysphagia appearing later. There was also word retrieval impairment early on within the language domain. By stage 7 a cluster of behaviour symptoms forms, with neuropsychiatric features occurring later, increasing in severity shortly after, as shown by the magenta in

the figure below. Hyperreligiosity occurs soon after neuropsychiatric symptoms, along with progression of altered sense of humour. A cluster forms in the cognition/function domain centred on executive function tasks (impaired judgement and problem-solving, problems with community affairs, problems at home or with hobbies) with these symptoms becoming severe (in black) at the earliest stage in this group, at around stage 78. A cluster of motor symptoms progressing occurs after this point with both bulbar and limb involvement.

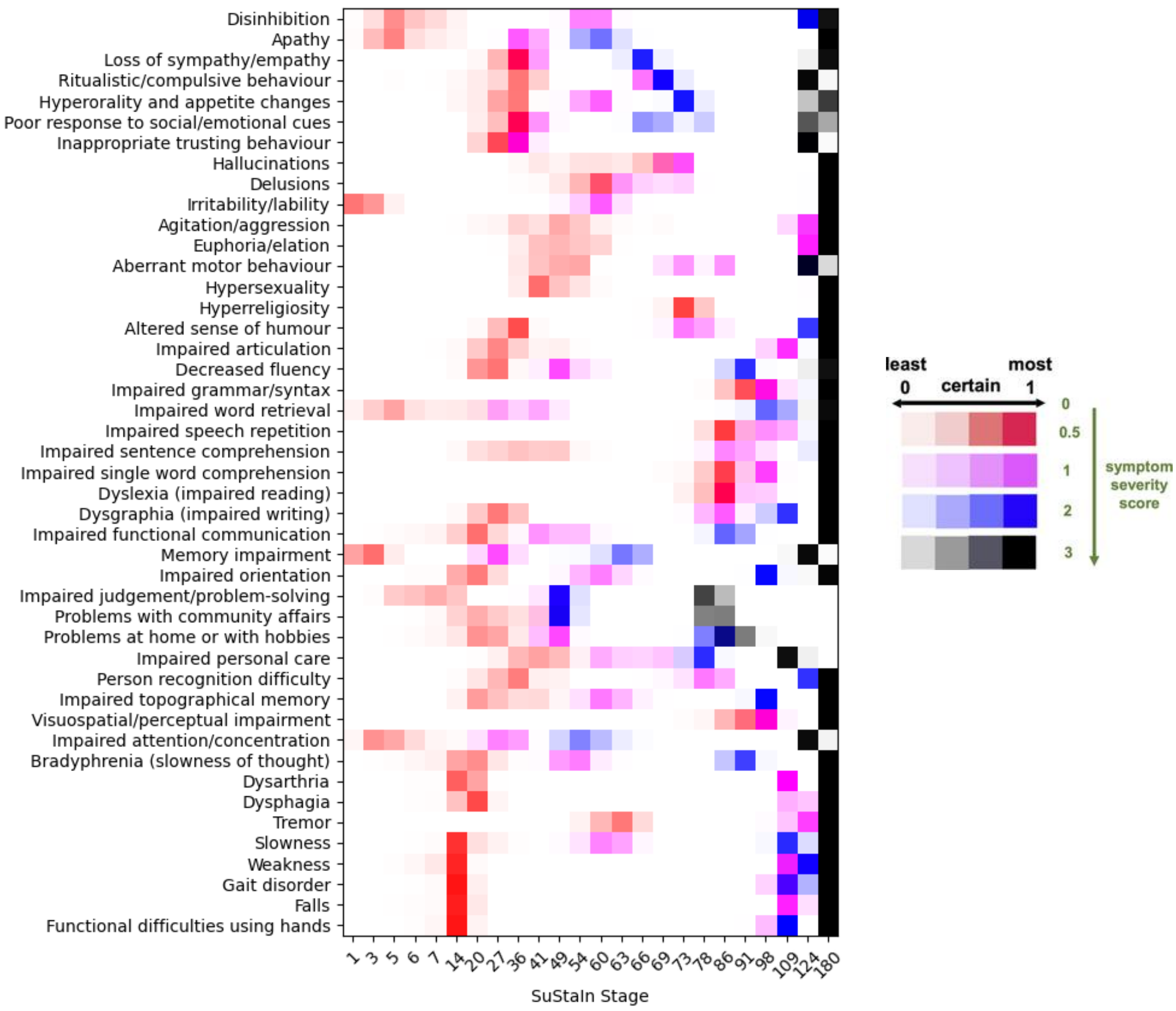

Supplementary Figure 7 shows a single *C9orf72* symptom progression pattern on an Ordinal SuStaIn Positional Variance Diagram (N = 134). Uncertainty in the SuStaIn progression patterns for each symptom severity score in this group are shown, where each square is shaded according to the probability a particular score is reached at a particular SuStaIn stage. The range includes: 0 = white, indicating no change; a score-change from 0 to 0.5 = red; from 0.5 to 1 = magenta; from 1 to 2 = blue; and from 2 to 3 = black. The lighter the shading the more uncertain the position of this score transition in the disease progression pattern.

GRN

The PVD for a single *GRN* symptom progression profile showed early cognition/function (impaired memory, attention/concentration and judgement/problem-solving, and problems with community affairs), behaviour (irritability/lability, apathy, loss of sympathy/empathy, hyperorality and appetite changes), and language (impaired word retrieval, sentence comprehension, grammar/syntax and functional communication, decreased fluency, dyslexia and dysgraphia) symptoms, with early deterioration in the language domain with impaired functional communication as the first symptom to become severely affected by stage 72 as shown in the figure below. A cluster of behaviour and cognition/function domain symptoms occur prior to significant deterioration in the language domain, with isolated dysarthria in the motor domain that coincides with increased severity of the language symptoms.

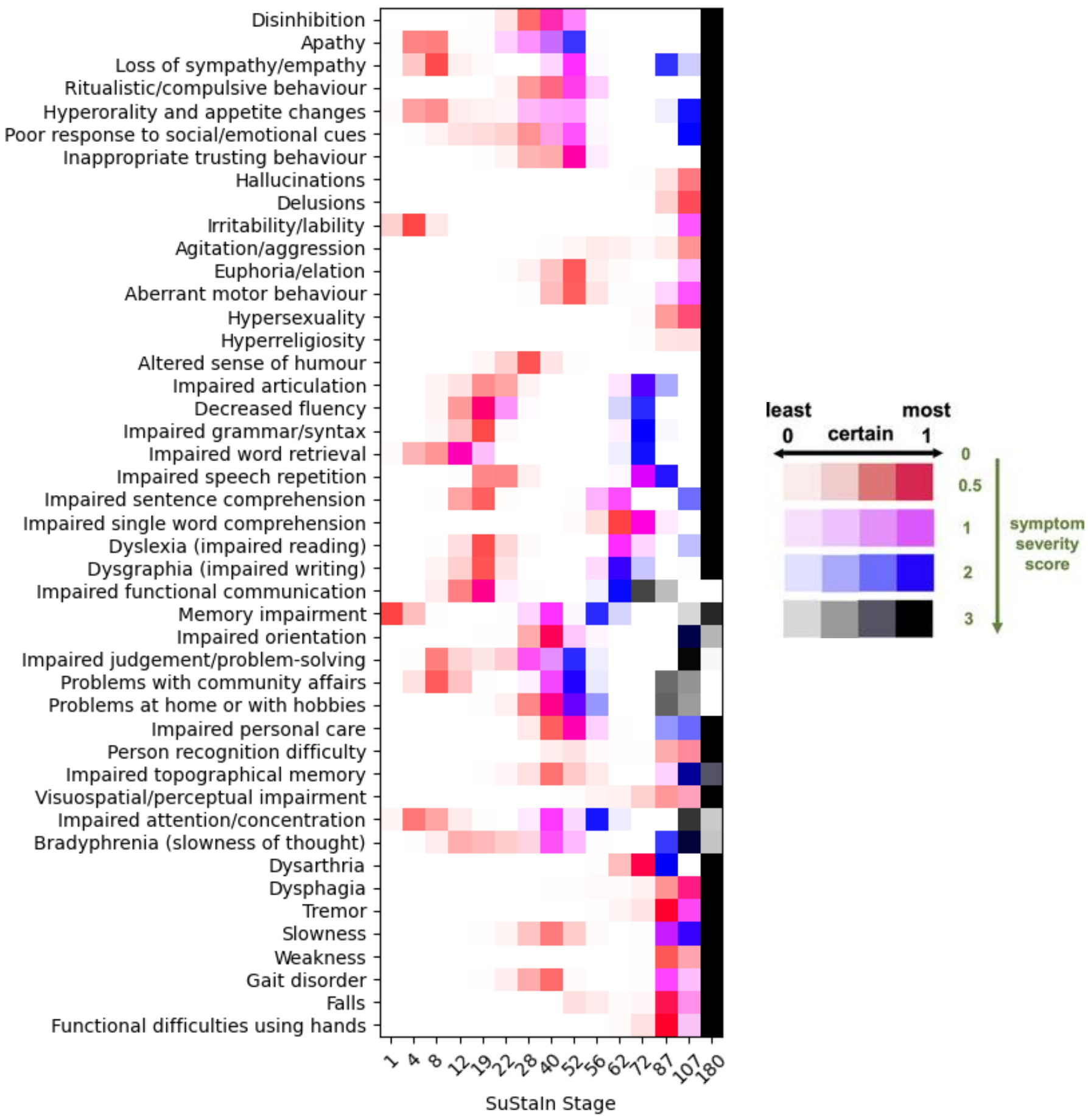

Supplementary Figure 8 shows a single *GRN* symptom progression pattern on an Ordinal SuStaIn Positional Variance Diagram (N = 90). Uncertainty in the SuStaIn progression patterns for each symptom severity score in this group are shown, where each square is shaded according to the probability a particular score is reached at a particular SuStaIn stage. The range includes: 0 = white, indicating no change; a score-change from 0 to 0.5 = red; from 0.5 to 1 = magenta; from 1 to 2 = blue; and from 2 to 3 = black. The lighter the shading the more uncertain the position of this score transition in the disease progression pattern.

MAPT

The PVD for a single *MAPT* symptom progression profile showed early impaired word retrieval, cognition/function (impaired memory, attention/concentration, orientation, topographical memory, judgement and problem-solving, person recognition difficulty, problems with community affairs, problems at home or with hobbies), and behaviour (irritability/lability, disinhibition, apathy, ritualistic/compulsive behaviour, altered sense of humour) symptoms, with early worsening of memory impairment and attention/concentration. Delusions occurred around the stages when behaviour symptoms worsen with a notable lack of hallucinations. Impaired word retrieval (a feature of semantic impairment) worsens at around stage 40 when impaired sentence comprehension, also a semantic deficit, emerges. Motor involvement, with initial slowness and gait disorder, along with bradyphrenia from the cognition/function domain, develops at the latter stages with functional difficulties using hands by stage 83 followed by widespread motor deterioration as shown in the below figure.

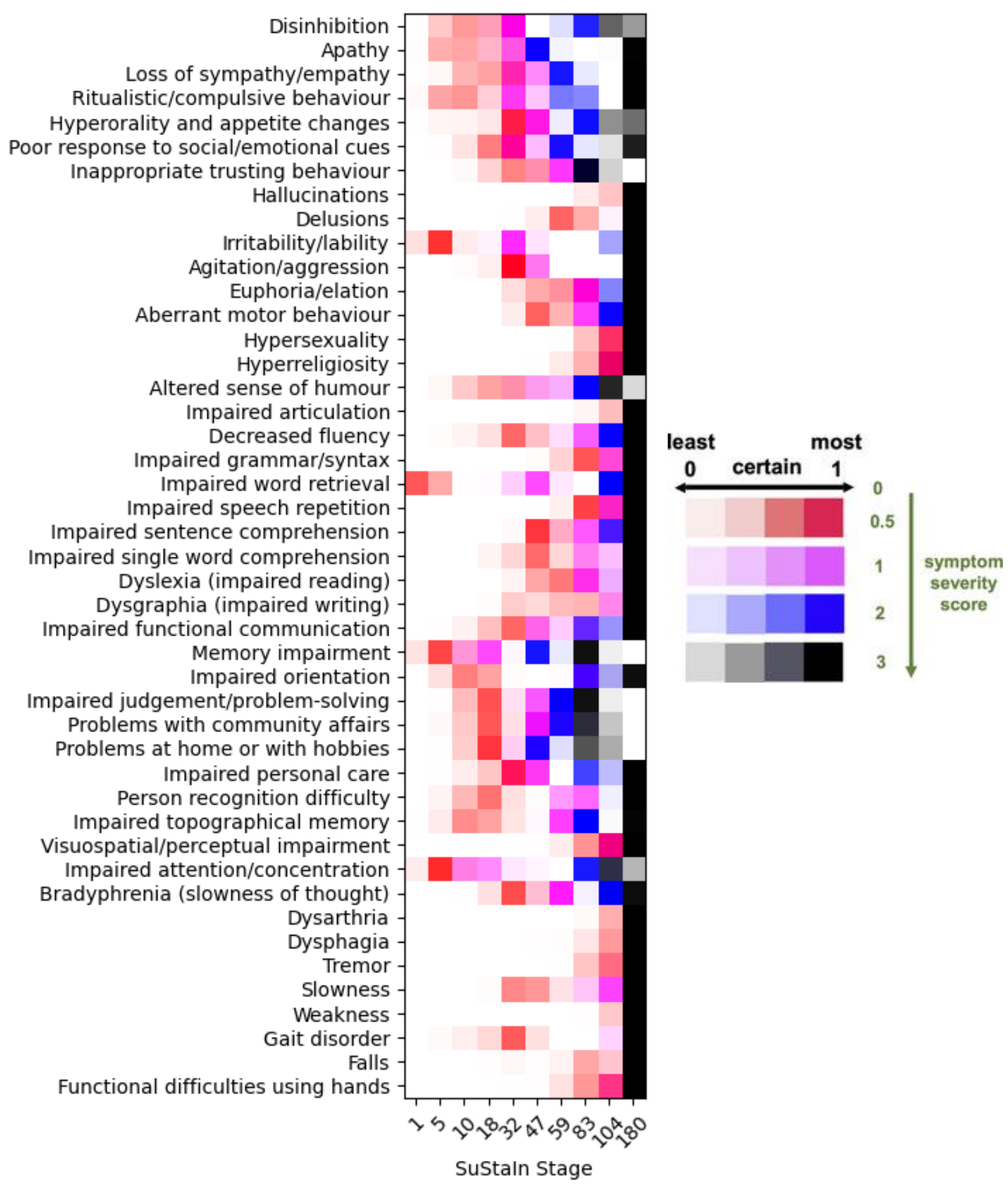

Supplementary Figure 9 shows a single *MAPT* symptom progression pattern on an Ordinal SuStaIn Positional Variance Diagram (N = 49). Uncertainty in the SuStaIn progression patterns for each symptom severity score in this group are shown, where each square is shaded according to the probability a particular score is reached at a particular SuStaIn stage. The range includes: 0 = white, indicating no change; a score-change from 0 to 0.5 = red; from 0.5 to 1 = magenta; from 1 to 2 = blue; and from 2 to 3 = black. The lighter the shading the more uncertain the position of this score transition in the disease progression pattern.

Early memory impairment and irritability/lability was a consistent feature across all mutation group progression patterns. All other symptoms varied in presence, certainty, and stage of presence in the disease process for each mutation group subtype.

Probability comparisons between single mutation subtypes

Mutation group probability comparisons detailed in the table below, based on the output from running SuStaIn singularly, aimed to show how much overlap if any there is between the genetic groups. The probability comparisons showed 60-80% of each of the mutation groups best align with the symptom progression pattern for their own mutation

group. 61.3% of the *C9orf72* mutation carriers best aligned with the symptom progression pattern learnt from their own mutation group, while 20.7% and 18.0% of the *C9orf72* mutation carriers best aligned with the symptom progression pattern learnt from *GRN* and *MAPT* mutation carriers, respectively. 60.2% of the *GRN* mutation carriers best aligned with the symptom progression pattern learnt from their own mutation group, while 24.3% and 15.5% best aligned with the symptom progression pattern learnt from the *MAPT* and *C9orf72* groups respectively. 77.8% of the *MAPT* mutation carriers best aligned with the symptom progression pattern learnt from their own mutation group, while 13.0% and 9.3% best aligned with the symptom progression pattern learnt from the *C9orf72* and *GRN* mutation carriers, respectively.

Supplementary Table 9 **shows mutation group probability comparisons using the SuStaIn model.** Subtype 0 represents a group where individuals had few or no symptoms and so were not analysable. Bold, italicised values represent those where the symptoms and their ordering within a single symptom progression profile are accounted for by its own mutation group.

	**Symptom progression profile (n):**				**Total with symptoms (n)**	**% symptoms accounted for by:**		
	**0**	***C9orf72***	***GRN***	***MAPT***		***C9orf72***	***GRN***	***MAPT***
C9orf72	130	**92**	31	27	150	***61.3***	20.7	18.0
GRN	139	16	**62**	25	103	15.5	***60.2***	24.3
MAPT	47	7	5	**42**	78	13.0	9.3	***77.8***

Appendix – List of GENFI Consortium Members

Rhian Convery	Department of Neurodegenerative Disease, Dementia Research Centre, UCL Queen Square Institute of Neurology, London, UK
Martina Bocchetta	Department of Neurodegenerative Disease, Dementia Research Centre, UCL Queen Square Institute of Neurology, London, UK
David Cash	Department of Neurodegenerative Disease, Dementia Research Centre, UCL Queen Square Institute of Neurology, London, UK
Sophie Goldsmith	Department of Neurodegenerative Disease, Dementia Research Centre, UCL Queen Square Institute of Neurology, London, UK
David L. Thomas	Neuroimaging Analysis Centre, Department of Brain Repair and Rehabilitation, UCL Institute of Neurology, Queen Square, London, UK
Thomas Cope	Cambridge University Hospitals NHS Trust, University of Cambridge, Cambridge, UK
Timothy Rittman	Department of Clinical Neurosciences, University of Cambridge, Cambridge, UK
Antonella Alberici	Centre for Neurodegenerative Disorders, Department of Clinical and Experimental Sciences, University of Brescia, Brescia, Italy
Enrico Premi	Stroke Unit, ASST Brescia Hospital, Brescia, Italy
Roberto Gasparotti	Neuroradiology Unit, University of Brescia, Brescia, Italy
Emanuele Buratti	ICGEB Trieste, Italy
Valentina Cantoni	Centre for Neurodegenerative Disorders, Department of Clinical and Experimental Sciences, University of Brescia, Brescia, Italy
Andrea Arighi	Fondazione IRCCS Ca' Granda Ospedale Maggiore Policlinico, Neurodegenerative Diseases Unit, Milan, Italy
Chiara Fenoglio	University of Milan, Centro Dino Ferrari, Milan, Italy
Vittoria Borracci	Fondazione IRCCS Ca' Granda Ospedale Maggiore Policlinico, Neurodegenerative Diseases Unit, Milan, Italy
Maria Serpente	Fondazione IRCCS Ca' Granda Ospedale Maggiore Policlinico, Neurodegenerative Diseases Unit, Milan, Italy
Tiziana Carandini	Fondazione IRCCS Ca' Granda Ospedale Maggiore Policlinico, Neurodegenerative Diseases Unit, Milan, Italy
Emanuela Rotondo	Fondazione IRCCS Ca' Granda Ospedale Maggiore Policlinico, Neurodegenerative Diseases Unit, Milan, Italy

Giacomina Rossi	Fondazione IRCCS Istituto Neurologico Carlo Besta, Milano, Italy
Giorgio Giaccone	Fondazione IRCCS Istituto Neurologico Carlo Besta, Milano, Italy
Giuseppe Di Fede	Fondazione IRCCS Istituto Neurologico Carlo Besta, Milano, Italy
Paola Caroppo	Fondazione IRCCS Istituto Neurologico Carlo Besta, Milano, Italy
Sara Prioni	Fondazione IRCCS Istituto Neurologico Carlo Besta, Milano, Italy
Veronica Redaelli	Fondazione IRCCS Istituto Neurologico Carlo Besta, Milano, Italy
David Tang-Wai	The University Health Network, Krembil Research Institute, Toronto, Canada
Ekaterina Rogaeva	Tanz Centre for Research in Neurodegenerative Diseases, University of Toronto, Toronto, Canada
Miguel Castelo-Branco	Faculty of Medicine, ICNAS, CIBIT, University of Coimbra, Coimbra, Portugal.
Morris Freedman	Baycrest Health Sciences, Rotman Research Institute, University of Toronto, Toronto, Canada
Ron Keren	The University Health Network, Toronto Rehabilitation Institute, Toronto, Canada
Sandra Black	Sunnybrook Health Sciences Centre, Sunnybrook Research Institute, University of Toronto, Toronto, Canada
Sara Mitchell	Sunnybrook Health Sciences Centre, Sunnybrook Research Institute, University of Toronto, Toronto, Canada
Christen Shoesmith	Department of Clinical Neurological Sciences, University of Western Ontario, London, Ontario, Canada
Robart Bartha	Department of Medical Biophysics, The University of Western Ontario, London, Ontario, Canada; Centre for Functional and Metabolic Mapping, Robarts Research Institute, The University of Western Ontario, London, Ontario, Canada
Rosa Rademakers	Center for Molecular Neurology, University of Antwerp
Jackie Poos	Department of Neurology, Erasmus Medical Center, Rotterdam, Netherlands
Janne M. Papma	Department of Neurology, Erasmus Medical Center, Rotterdam, Netherlands
Lucia Giannini	Department of Neurology, Erasmus Medical Center, Rotterdam, Netherlands
Liset de Boer	Department of Neurology, Erasmus Medical Center, Rotterdam, Netherlands
Julie de Houwer	Department of Neurology, Erasmus Medical Center, Rotterdam, Netherlands
Rick van Minkelen	Department of Clinical Genetics, Erasmus Medical Center, Rotterdam, Netherlands
Yolande Pijnenburg	Amsterdam University Medical Centre, Amsterdam VUmc, Amsterdam, Netherlands
Benedetta Nacmias	Department of Neuroscience, Psychology, Drug Research and Child Health, University of Florence, Florence, Italy

Camilla Ferrari	Department of Neuroscience, Psychology, Drug Research and Child Health, University of Florence, Florence, Italy
Cristina Polito	Department of Biomedical, Experimental and Clinical Sciences "Mario Serio", Nuclear Medicine Unit, University of Florence, Florence, Italy
Gemma Lombardi	Department of Neuroscience, Psychology, Drug Research and Child Health, University of Florence, Florence, Italy
Valentina Bessi	Department of Neuroscience, Psychology, Drug Research and Child Health, University of Florence, Florence, Italy
Mattias Nilsson	Department of Clinical Neuroscience, Karolinska Institutet, Stockholm, Sweden
Henrik Viklund	Karolinska University Hospital Huddinge
Melissa Taheri Rydell	Department of Neurobiology, Care Sciences and Society; Center for Alzheimer Research, Division of Neurogeriatrics, , Bioclinicum, Karolinska Institutet, Solna, Sweden; Unit for Hereditary Dementias, Theme inflammation and Aging, Karolinska University Hospital, Solna, Sweden
Vesna Jelic	Department of Neurobiology, Care Sciences and Society; Division of Clinical Geriatrics, Karolinska Institutet, Stockholm, Sweden; Cognitive clinic, Theme inflammation and Aging, Karolinska University Hospital, Solna, Sweden
Linn Öijerstedt	Department of Neurobiology, Care Sciences and Society; Center for Alzheimer Research, Division of Neurogeriatrics, , Bioclinicum, Karolinska Institutet, Solna, Sweden; Unit for Hereditary Dementias, Theme inflammation and Aging, Karolinska University Hospital, Solna, Sweden
Tobias Langheinrich	Division of Neuroscience and Experimental Psychology, Wolfson Molecular Imaging Centre, University of Manchester, Manchester, UK; Manchester Centre for Clinical Neurosciences, Department of Neurology, Salford Royal NHS Foundation Trust, Manchester, UK
Albert Lladó	Alzheimer's disease and Other Cognitive Disorders Unit, Neurology Service, Hospital Clínic, Barcelona, Spain
Anna Antonell	Alzheimer's disease and Other Cognitive Disorders Unit, Neurology Service, Hospital Clínic, Barcelona, Spain
Jaume Olives	Alzheimer's disease and Other Cognitive Disorders Unit, Neurology Service, Hospital Clínic, Barcelona, Spain
Mircea Balasa	Alzheimer's disease and Other Cognitive Disorders Unit, Neurology Service, Hospital Clínic, Barcelona, Spain
Nuria Bargalló	Imaging Diagnostic Center, Hospital Clínic, Barcelona, Spain

Sergi Borrego-Ecija	Alzheimer's disease and Other Cognitive Disorders Unit, Neurology Service, Hospital Clínic, Barcelona, Spain
Ana Verdelho	Department of Neurosciences and Mental Health, Centro Hospitalar Lisboa Norte - Hospital de Santa Maria & Faculty of Medicine, University of Lisbon, Lisbon, Portugal
Carolina Maruta	Laboratory of Language Research, Centro de Estudos Egas Moniz, Faculty of Medicine, University of Lisbon, Lisbon, Portugal
Tiago Coelho	Faculty of Medicine, University of Lisbon, Lisbon, Portugal
Gabriel Miltenberger	Faculty of Medicine, University of Lisbon, Lisbon, Portugal
Frederico Simões do Couto	Faculdade de Medicina, Universidade Católica Portuguesa
Alazne Gabilondo	Cognitive Disorders Unit, Department of Neurology, Donostia University Hospital, San Sebastian, Gipuzkoa, Spain; Neuroscience Area, Biodonostia Health Research Insitute, San Sebastian, Gipuzkoa, Spain
Ioana Croitoru	Neuroscience Area, Biodonostia Health Research Insitute, San Sebastian, Gipuzkoa, Spain
Mikel Tainta	Neuroscience Area, Biodonostia Health Research Insitute, San Sebastian, Gipuzkoa, Spain
Myriam Barandiaran	Cognitive Disorders Unit, Department of Neurology, Donostia University Hospital, San Sebastian, Gipuzkoa, Spain; Neuroscience Area, Biodonostia Health Research Insitute, San Sebastian, Gipuzkoa, Spain
Patricia Alves	Neuroscience Area, Biodonostia Health Research Insitute, San Sebastian, Gipuzkoa, Spain; Department of Educational Psychology and Psychobiology, Faculty of Education, International University of La Rioja, Logroño, Spain
Benjamin Bender	Department of Diagnostic and Interventional Neuroradiology, University of Tübingen, Tübingen, Germany
David Mengel	Department of Neurodegenerative Diseases, Hertie-Institute for Clinical Brain Research and Center of Neurology, University of Tübingen, Tübingen, Germany; Center for Neurodegenerative Diseases (DZNE), Tübingen, Germany
Lisa Graf	Department of Neurodegenerative Diseases, Hertie-Institute for Clinical Brain Research and Center of Neurology, University of Tübingen, Tübingen, Germany
Annick Vogels	Department of Human Genetics, KU Leuven, Leuven, Belgium
Mathieu Vandenbulcke	Geriatric Psychiatry Service, University Hospitals Leuven, Belgium; Neuropsychiatry, Department of Neurosciences, KU Leuven, Leuven, Belgium

Philip Van Damme	Neurology Service, University Hospitals Leuven, Belgium; Laboratory for Neurobiology, VIB-KU Leuven Centre for Brain Research, Leuven, Belgium
Rose Bruffaerts	Department of Biomedical Sciences, University of Antwerp, Antwerp, Belgium; Biomedical Research Institute, Hasselt University, 3500 Hasselt, Belgium
Koen Poesen	Laboratory for Molecular Neurobiomarker Research, KU Leuven, Leuven, Belgium
Pedro Rosa-Neto	Translational Neuroimaging Laboratory, McGill Centre for Studies in Aging, McGill University, Montreal, Québec, Canada
Maxime Montembault	Douglas Research Centre, Department of Psychiatry, McGill University, Montreal, Québec, Canada
Agnès Camuzat	Sorbonne Université, Paris Brain Institute – Institut du Cerveau – ICM, Inserm U1127, CNRS UMR 7225, AP-HP - Hôpital Pitié-Salpêtrière, Paris, France
Alexis Brice	Sorbonne Université, Paris Brain Institute – Institut du Cerveau – ICM, Inserm U1127, CNRS UMR 7225, AP-HP - Hôpital Pitié-Salpêtrière, Paris, France; Reference Network for Rare Neurological Diseases (ERN-RND)
Anne Bertrand	Sorbonne Université, Paris Brain Institute – Institut du Cerveau – ICM, Inserm U1127, CNRS UMR 7225, AP-HP - Hôpital Pitié-Salpêtrière, Paris, France; Inria, Aramis project-team, F-75013, Paris, France; Centre pour l'Acquisition et le Traitement des Images, Institut du Cerveau et la Moelle, Paris, France
Aurélie Funkiewiez	Sorbonne Université, Paris Brain Institute – Institut du Cerveau – ICM, Inserm U1127, CNRS UMR 7225, AP-HP - Hôpital Pitié-Salpêtrière, Paris, France; Centre de référence des démences rares ou précoces, IM2A, Département de Neurologie, AP-HP - Hôpital Pitié-Salpêtrière, Paris, France
Daisy Rinaldi	Sorbonne Université, Paris Brain Institute – Institut du Cerveau – ICM, Inserm U1127, CNRS UMR 7225, AP-HP - Hôpital Pitié-Salpêtrière, Paris, France; Centre de référence des démences rares ou précoces, IM2A, Département de Neurologie, AP-HP - Hôpital Pitié-Salpêtrière, Paris, France; Département de Neurologie, AP-HP - Hôpital Pitié-Salpêtrière, Paris, France
Dario Saracino	Sorbonne Université, Paris Brain Institute – Institut du Cerveau – ICM, Inserm U1127, CNRS UMR 7225, AP-HP - Hôpital Pitié-Salpêtrière, Paris, France; Centre de référence des démences rares ou précoces, IM2A, Département de Neurologie, AP-HP - Hôpital Pitié-Salpêtrière, Paris, France; Inria, Aramis project-team, F-75013, Paris, France
Olivier Colliot	Sorbonne Université, Paris Brain Institute – Institut du Cerveau – ICM, Inserm U1127, CNRS UMR 7225, AP-HP - Hôpital Pitié-Salpêtrière, Paris, France;

	Centre pour l'Acquisition et le Traitement des Images, Institut du Cerveau et la Moelle, Paris, France; Inria, Aramis project-team, F-75013, Paris, France
Sabrina Sayah	Sorbonne Université, Paris Brain Institute – Institut du Cerveau – ICM, Inserm U1127, CNRS UMR 7225, AP-HP - Hôpital Pitié-Salpêtrière, Paris, France
Catharina Prix	Neurologische Klinik, Ludwig-Maximilians-Universität München, Munich, Germany
Elisabeth Wlasich	Neurologische Klinik, Ludwig-Maximilians-Universität München, Munich, Germany
Olivia Wagemann	Neurologische Klinik, Ludwig-Maximilians-Universität München, Munich, Germany
Sonja Schönecker	Neurologische Klinik, Ludwig-Maximilians-Universität München, Munich, Germany
Alexander Maximilian Bernhardt	Neurologische Klinik, Ludwig-Maximilians-Universität München, Munich, Germany
Anna Stockbauer	Neurologische Klinik, Ludwig-Maximilians-Universität München, Munich, Germany
Jolina Lombardi	Department of Neurology, University of Ulm, Ulm
Sarah Anderl-Straub	Department of Neurology, University of Ulm, Ulm, Germany
Adeline Rollin	CHU, CNR-MAJ, Labex Distalz, LiCEND Lille, France
Gregory Kuchcinski	Univ Lille, France; Inserm 1172, Lille, France; CHU, CNR-MAJ, Labex Distalz, LiCEND Lille, France
Maxime Bertoux	Inserm 1172, Lille, France; CHU, CNR-MAJ, Labex Distalz, LiCEND Lille, France
Thibaud Lebouvier	Univ Lille, France; Inserm 1172, Lille, France; CHU, CNR-MAJ, Labex Distalz, LiCEND Lille, France
Vincent Deramecourt	Univ Lille, France; Inserm 1172, Lille, France; CHU, CNR-MAJ, Labex Distalz, LiCEND Lille, France
João Durães	Neurology Department, Centro Hospitalar e Universitario de Coimbra, Coimbra, Portugal
Marisa Lima	Neurology Department, Centro Hospitalar e Universitario de Coimbra, Coimbra, Portugal
Maria João Leitão	Centre of Neurosciences and Cell Biology, Universidade de Coimbra, Coimbra, Portugal
Maria Rosario Almeida	Faculty of Medicine, University of Coimbra, Coimbra, Portugal

Miguel Tábuas-Pereira	Neurology Department, Centro Hospitalar e Universitario de Coimbra, Coimbra, Portugal; Faculty of Medicine, University of Coimbra, Coimbra, Portugal.
Sónia Afonso	Instituto Ciencias Nucleares Aplicadas a Saude, Universidade de Coimbra, Coimbra, Portugal
João Lemos	Faculty of Medicine, University of Coimbra, Coimbra, Portugal
